# Supplementary figures and images for: The O-GlcNAc transferase OGT is a conserved and essential regulator of the cellular and organismal response to hypertonic stress
Source: PLoS Genet. 2020 Oct 2;16(10):e1008821. doi: 10.1371/journal.pgen.1008821 (PMC7556452; doi:10.1371/journal.pgen.1008821)

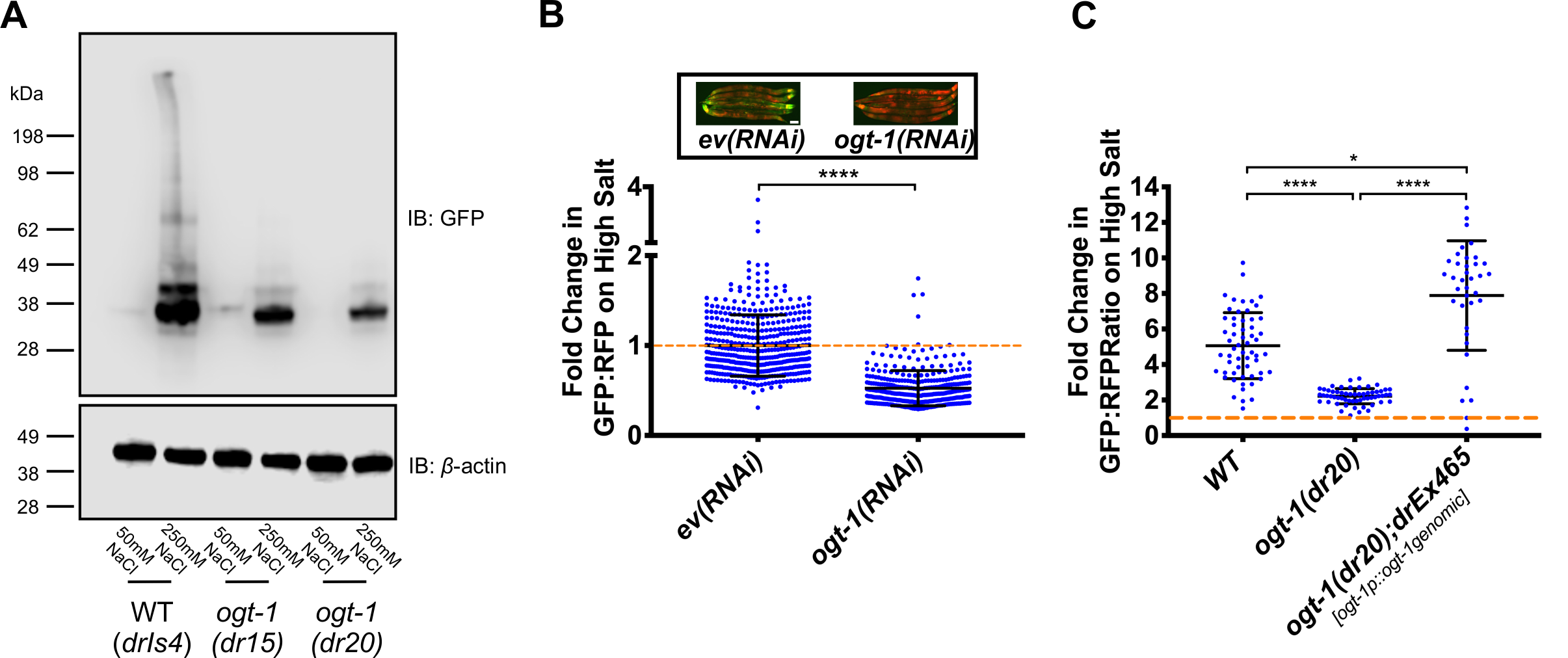

Supplement: S1 Fig — (A) Immunoblot of GFP and β-actin in lysates from WT and ogt-1 mutant animals expressing drIs4 exposed to 50 or 250 mM NaCl NGM plates for 18 hours. (B) COPAS Biosort quantification of GFP and RFP signal in day 2 adult WT animals expressing drIs4 exposed to 50 or 250 mM NaCl NGM plates for 18 hours. Animals were grown on ev(RNAi) or ogt-1(RNAi) plates for multiple generations. Data are represented as fold induction of normalized GFP/RFP ratio on 250 mM NaCl RNAi plates relative to on 50 mM NaCl RNAi plates. Each point represents the quantified signal from a single animal. Data are expressed as mean ± S.D. ****—p<0.0001 (Mann-Whitney test). N ≥ 334 for each group. Inset: Wide-field fluorescence microscopy of day 2 adult animals expressing drIs4 exposed to 250 mM NaCl NGM plates for 18 hours. Animals were grown on ev(RNAi) or ogt-1(RNAi) plates for multiple generations. Images depict merged GFP and RFP channels. Scale bar = 100 microns. (C) COPAS Biosort quantification of GFP and RFP signal in day 2 adult animals expressing drIs4 exposed to 50 or 250 mM NaCl NGM plates for 18 hours in the indicated genetic background. drEx465 is an extrachromosomal array expressing a 10.3 Kb ogt-1 genomic DNA fragment containing ~2Kb upstream and ~1Kb downstream of the ogt-1 coding sequence. Data are represented as fold induction of normalized GFP/RFP ratio on 250 mM NaCl NGM plates relative to on 50 mM NaCl NGM plates. Each point represents the quantified signal from a single animal. Data are expressed as mean ± S.D. ****—p<0.0001, *—p<0.05 (Kruskal-Wallis test with post hoc Dunn’s test). N ≥ 37 for each group. (TIF) [file pgen.1008821.s001.tif]

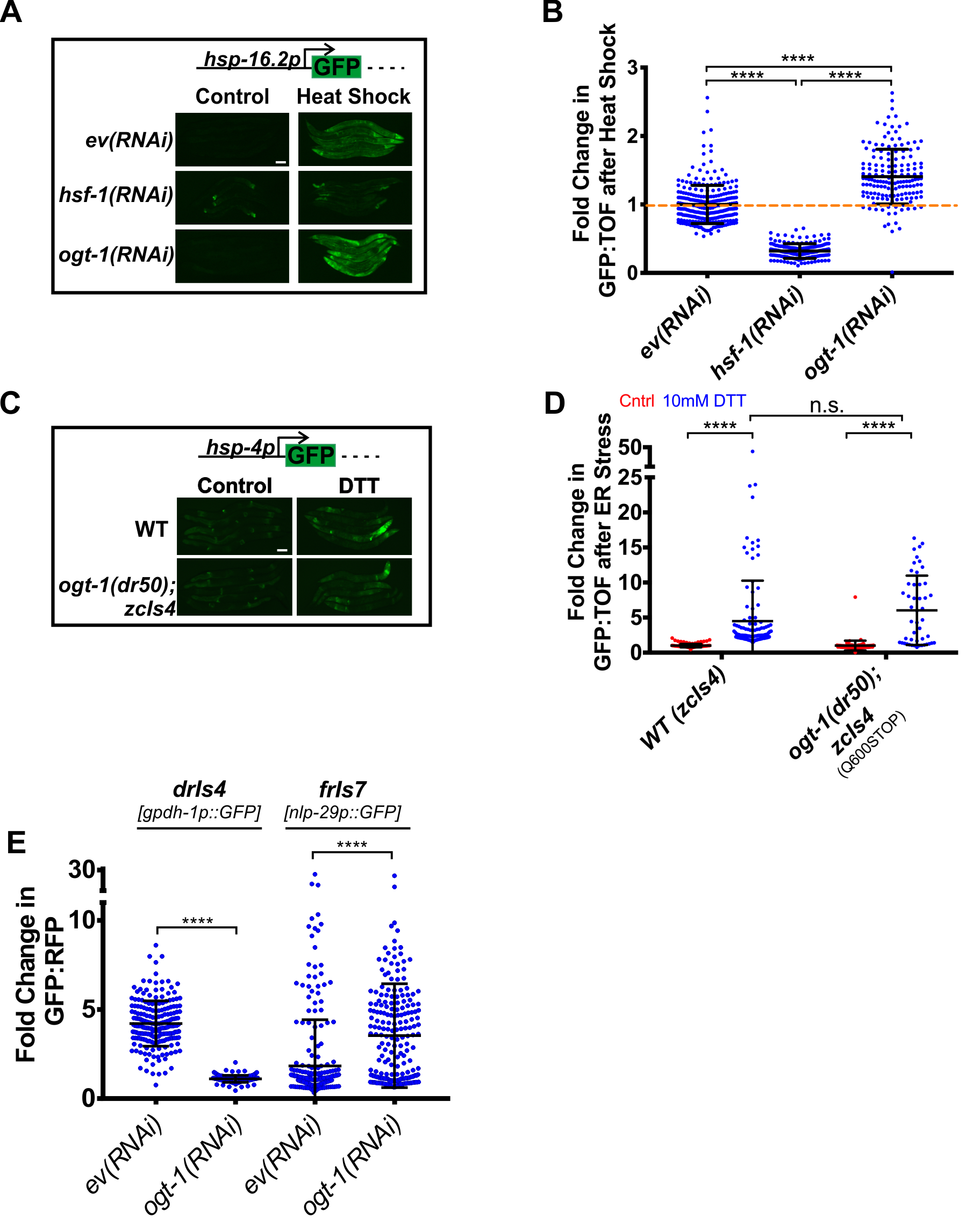

Supplement: S2 Fig — (A) Wide-field fluorescence microscopy of day 2 adult animals expressing hsp-16.2p::GFP (gpIs1) grown on ev(RNAi), ogt-1(RNAi), or hsf-1(RNAi) plates and exposed to control or heat shock conditions (35°C for 3 hours, 18 hour recovery at 20°C). Images depict the GFP channel, since there is not a normalizing RFP reporter in these strains. Scale bar = 100 microns. (B) COPAS Biosort quantification of GFP and TOF signal from animals in (A). Data are represented as fold induction of normalized GFP/TOF ratio of animals exposed to heat shock conditions relative to animals exposed to control conditions. Each point represents the quantified signal from a single animal. Data are expressed as mean ± S.D. ****—p<0.0001 (Kruskal-Wallis test with post hoc Dunn’s test). N ≥ 158 for each group. (C) Wide-field fluorescence microscopy of day 2 adult animals expressing hsp-4p::GFP (zcIs4) exposed to DTT plates for 18 hours. The ogt-1(dr50) allele carries the same homozygous Q600STOP mutation as the ogt-1(dr20) allele and was introduced using CRISPR/Cas9. Images depict the GFP channel. Scale bar = 100 microns. (D) COPAS Biosort quantification of GFP and TOF signal from animals in (C). Data are represented as fold induction of normalized GFP/TOF ratio of animals exposed to DTT plates relative to animals exposed to control plates. Each point represents the quantified signal from a single animal. Data are expressed as mean ± S.D. ****—p<0.0001, n.s = nonsignificant (Kruskal-Wallis test with post hoc Dunn’s test). N ≥ 48 for each group. (E) COPAS Biosort quantification of GFP and RFP signal from day 2 animals expressing gpdh-1p::GFP (drIs4) or nlp-29::GFP (frIs7) exposed to 250 mM NaCl for 18 and 24 hours respectively. Data are represented as fold induction of normalized GFP/RFP ratio of animals exposed to 250 mM NaCl plates relative to animals exposed to 50 mM NaCl plates. Each point represents the quantified signal from a single animal. Data are expressed as mean ± S.D. ****—p<0.0001 (Kru [file pgen.1008821.s002.tif]

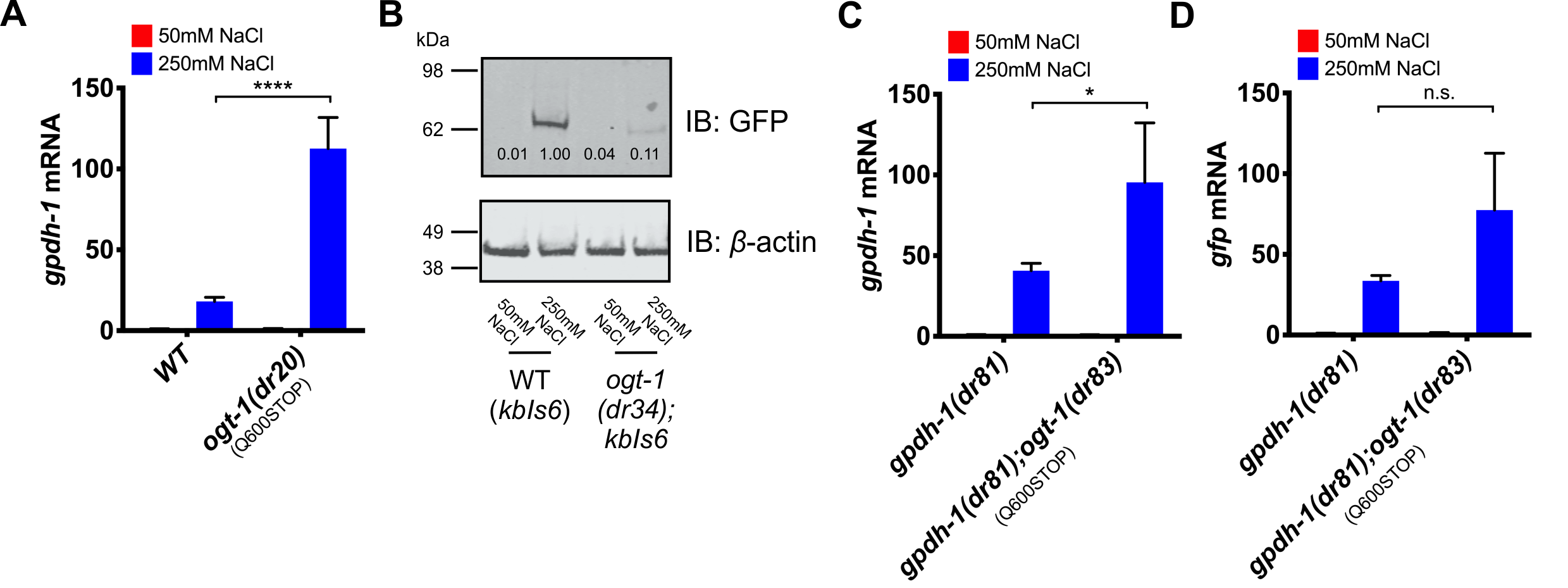

Supplement: S3 Fig — (A) qPCR of gpdh-1 mRNA in day 2 adult animals expressing drIs4 exposed to 50 or 250 mM NaCl NGM plates for 3 hours. Strains include WT and ogt-1(dr20). Data are represented as fold induction of RNA on 250 mM NaCl relative to 50 mM NaCl. Data are expressed as mean ± S.D. ****—p<0.0001 (Student’s two-tailed t-test). N = 3 biological replicates of 35 animals for each group. (B) Immunoblot of GFP and β-actin in lysates from WT and ogt-1(dr34) animals expressing the kbIs6 translational fusion exposed to 50 or 250 mM NaCl NGM plates for 18 hours. The ogt-1(dr34) allele carries the same homozygous Q600STOP mutation as the ogt-1(dr20) allele and was introduced using CRISPR/Cas9. Numbers under the GFP bands represent GFP signal normalized to β-actin signal for each sample, with the WT 250 mM NaCl sample set to 1. (C) qPCR of gpdh-1 mRNA from day 2 adult animals exposed to 50 or 250 mM NaCl NGM plates for 24 hours. The animals express a CRISPR/Cas9 edited knock-in of GFP into the endogenous gpdh-1 gene (gpdh-1(dr81)). ogt-1 carries the dr83 allele, which is the same homozygous Q600STOP mutation as the ogt-1(dr20) allele and was introduced using CRISPR/Cas9. Data are represented as fold induction of RNA on 250 mM NaCl relative to 50 mM NaCl. Data are expressed as mean ± S.D. *—p<0.05 (Student’s two-tailed t-test). N ≥ 3 biological replicates of 35 animals for each group. (D) qPCR of gfp mRNA from day 2 animals) exposed to 50 or 250 mM NaCl NGM plates for 24 hours. The strains are the same as in (C). Data are represented as fold induction of RNA on 250 mM NaCl relative to 50 mM NaCl. Data are expressed as mean ± S.D. n.s. = nonsignificant (Student’s two-tailed t-test). N ≥ 3 biological replicates of 35 animals for each group. (TIF) [file pgen.1008821.s003.tif]

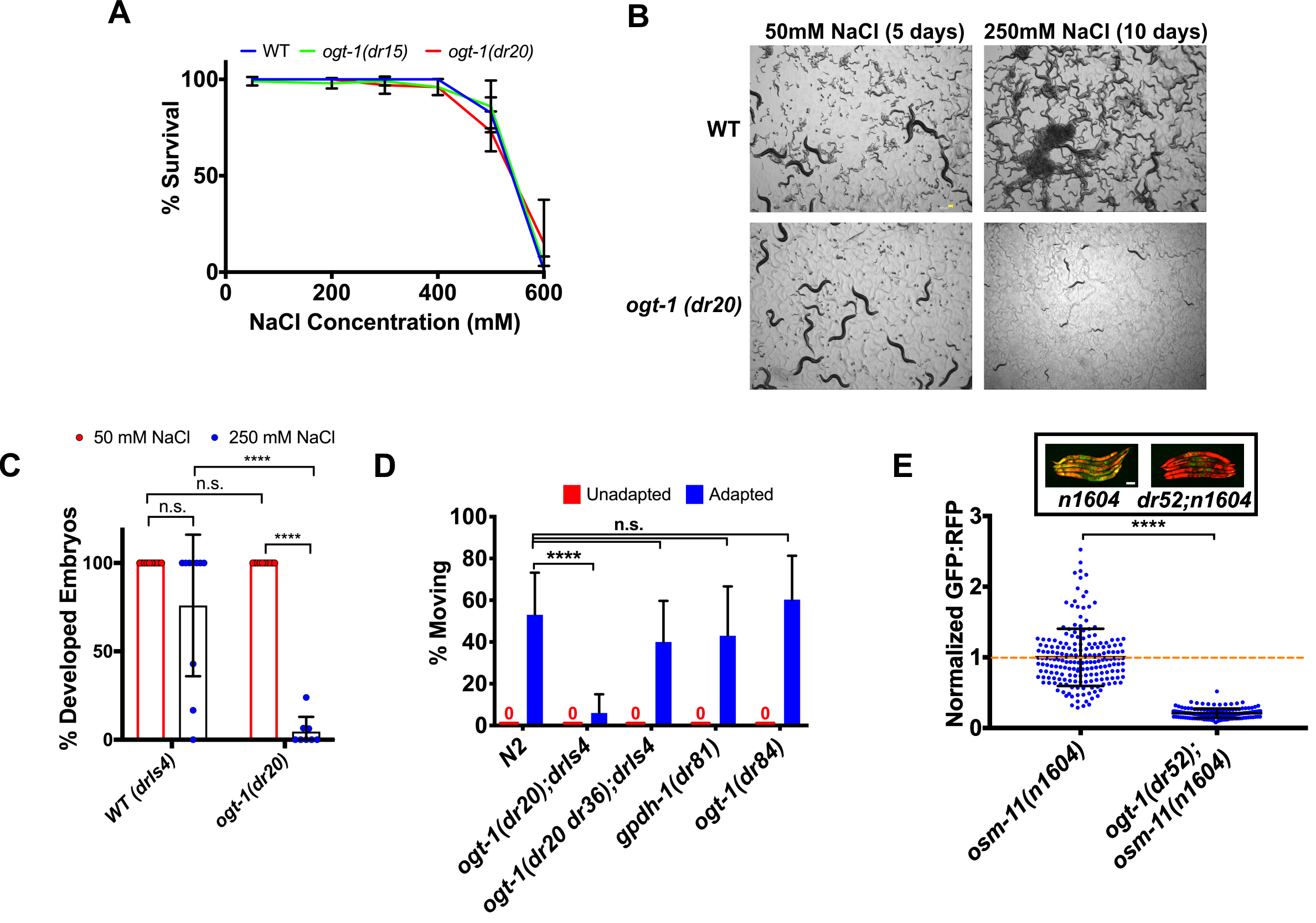

Supplement: S4 Fig — (A) Percent survival of day 2 adult animals expressing drIs4 exposed to 100–600 mM NaCl NGM plates for 24 hours. Strains include WT, ogt-1(dr15), and ogt-1(dr20). Data are expressed as mean ± S.D. N = 5 replicates of 20 animals for each salt concentration. (B) Brightfield microscopy images of animals grown on 50 mM or 250 mM NaCl for 5 and 10 days respectively. Strains include WT (drIs4) and ogt-1(dr20);drIs4. Scale bar = 100 microns (C) Percent of progeny that developed into L4s. L4 animals were placed on 50 or 250 mM NaCl and the total number of eggs laid and progeny that developed into L4s were counted each day as described in the ‘Methods’. Data are expressed as mean ± S.D. ****—p<0.0001, **—p<0.01 (One-way ANOVA with post hoc Tukey’s test). N = 10 independent broods for each strain. (D) Percent of moving unadapted and adapted day 3 adult animals after exposure to 600 mM NaCl NGM plates for 24 hours. ogt-1(dr20 dr36) is a strain in which the dr20 mutation was converted back to WT using CRISPR/Cas9 genome editing. The gpdh-1(dr81) allele is a CRISPR/Cas9 edited C-terminal knock-in of GFP into the endogenous gpdh-1. The ogt-1(dr84) allele is a CRISPR/Cas9 edited C-terminal knock-in of GFP into the endogenous ogt-1. Data are expressed as mean ± S.D. ****—p<0.0001, n.s. = nonsignificant (One-way ANOVA with post hoc Dunnett’s test). N = 5 replicates of 20 animals for each strain. (E) COPAS Biosort quantification of GFP and RFP signal in day 2 adult drIs4 animals drIs4 exposed to 50 mM NaCl NGM plates. Data are represented as the fold induction of normalized GFP/RFP ratio on 50 mM NaCl NGM plates, with osm-11(n1604) set to 1. The ogt-1(dr52) allele carries the same homozygous Q600STOP mutation as the ogt-1(dr20) allele, which was introduced using CRISPR/Cas9. Each point represents the quantified signal from a single animal. Data are expressed as mean ± S.D. ****—p<0.0001 (Mann-Whitney test). N ≥ 163 for each group. Inset: Wide-field fluorescence microscopy of day 2 ad [file pgen.1008821.s004.tif]

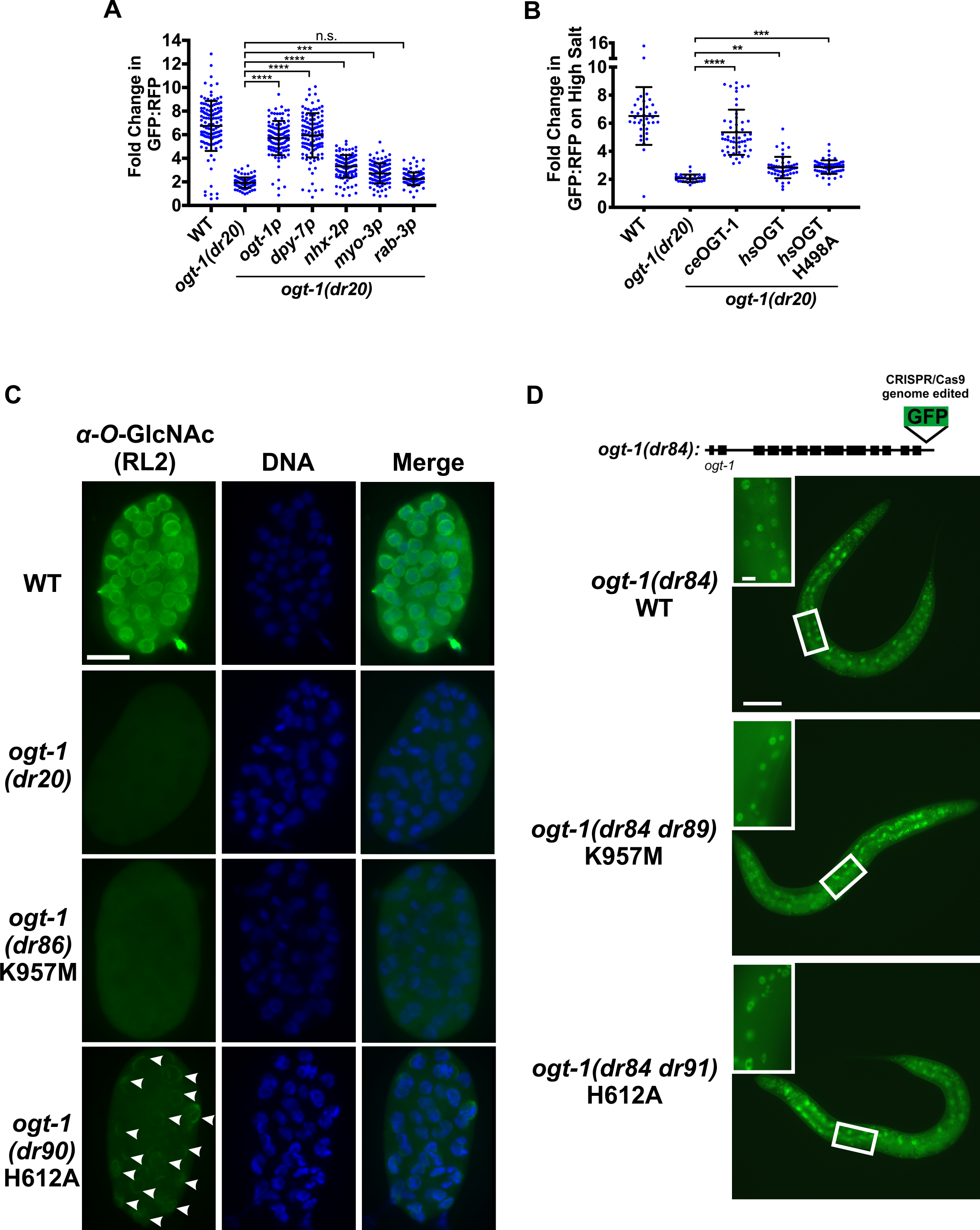

Supplement: S5 Fig — (A) COPAS Biosort quantification of GFP and RFP signal in day 2 adult animals expressing drIs4 exposed to 50 or 250 mM NaCl NGM plates for 18 hours. Data are represented as the fold induction of normalized GFP/RFP ratio on 250 mM NaCl NGM plates relative to on 50 mM NaCl NGM plates. Each point represents the quantified signal from a single animal. Data are expressed as mean ± S.D. ***—p<0.001, ****—p<0.0001, n.s. = nonsignificant (Kruskal-Wallis test with post hoc Dunn’s test). N ≥ 37 for each group. These data were used to calculate the ‘Degree of Rescue’ in Fig 5B. (B) COPAS Biosort quantification of GFP and RFP signal in day 2 adult animals expressing drIs4 exposed to 50 or 250 mM NaCl NGM plates for 18 hours. Data are represented as the fold induction of normalized GFP/RFP ratio on 50 and 250 mM NaCl NGM plates relative to on 50 mM NaCl NGM plates. Each point represents the quantified signal from a single animal. Data are expressed as mean ± S.D. ****—p<0.0001, ***—p <0.001, **—p<0.01 (Kruskal-Wallis test with post hoc Dunn’s test). N ≥ 40 for each group. These data were used to calculate the ‘Degree of Rescue’ in Fig 5D. (C) Wide-field fluorescence microscopy of fixed and stained embryos. RL2 was used to stain for nuclear pore O-GlcNAc modifications and Hoechst 33258 was used to visualize the DNA. Images are exposure matched. White arrowheads indicate RL2 staining in OGT-1H612A embryos. Scale bar = 10 microns. (D) Wide-field fluorescence microscopy of day 1 adult animals expressing endogenously CRISPR/Cas9 GFP tagged OGT-1 exposed to 50 mM NaCl NGM plates. Scale bar = 100 microns. Images are exposure matched. Inset: Zoomed in images of the boxed area. Scale bar = 10 microns. (TIF) [file pgen.1008821.s005.tif]

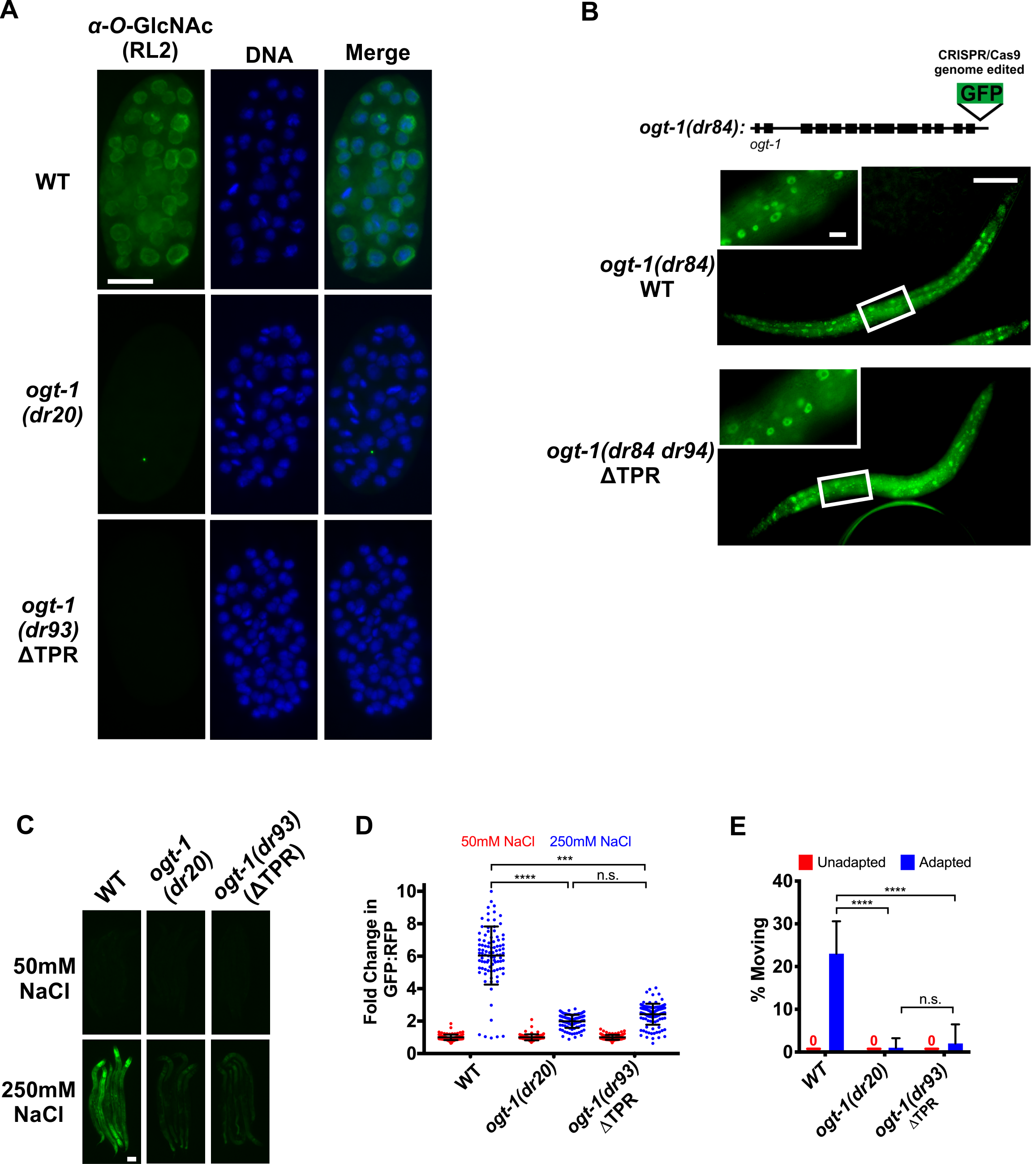

Supplement: S6 Fig — (A) Wide-field fluorescence microscopy of fixed and stained embryos. RL2 was used to stain for nuclear pore O-GlcNAc modifications and Hoechst 33258 was used to visualize the DNA. Images are exposure matched. Scale bar = 10 microns. (B) Wide-field fluorescence microscopy of day 1 adult animals expressing endogenously CRISPR/Cas9 GFP tagged OGT-1 exposed to 50 mM NaCl NGM plates. Scale bar = 100 microns. Images are exposure matched. Inset: Zoomed in images of the boxed area. Scale bar = 10 microns. (C) Wide-field fluorescence microscopy of day 2 adult animals expressing drIs4 exposed to 50 or 250 mM NaCl NGM plates for 18 hours. Images depict the GFP channel only for clarity. The RFP signal was unaffected in these rescue strains (not shown). Scale bar = 100 microns. (D) COPAS Biosort quantification of GFP and RFP signal in day 2 adult animals expressing drIs4 exposed to 50 or 250 mM NaCl NGM plates for 18 hours. Data are represented as the fold induction of normalized GFP/RFP ratio on 50 and 250 mM NaCl NGM plates relative to on 50 mM NaCl NGM plates. Each point represents the quantified signal from a single animal. Data are expressed as mean ± S.D. ****—p<0.0001, ***—p<0.001, n.s. = nonsignificant (Kruskal-Wallis test with post hoc Dunn’s test). N ≥ 92 for each group. (E) Percent of moving unadapted and adapted day 3 adult animals expressing drIs4 exposed to 600 mM NaCl NGM plates for 24 hours. Data are expressed as mean ± S.D. ****—p<0.0001, n.s. = nonsignificant (One-way ANOVA with post hoc Tukey’s test). N = 5 replicates of 20 animals for each strain. (TIF) [file pgen.1008821.s006.tif]

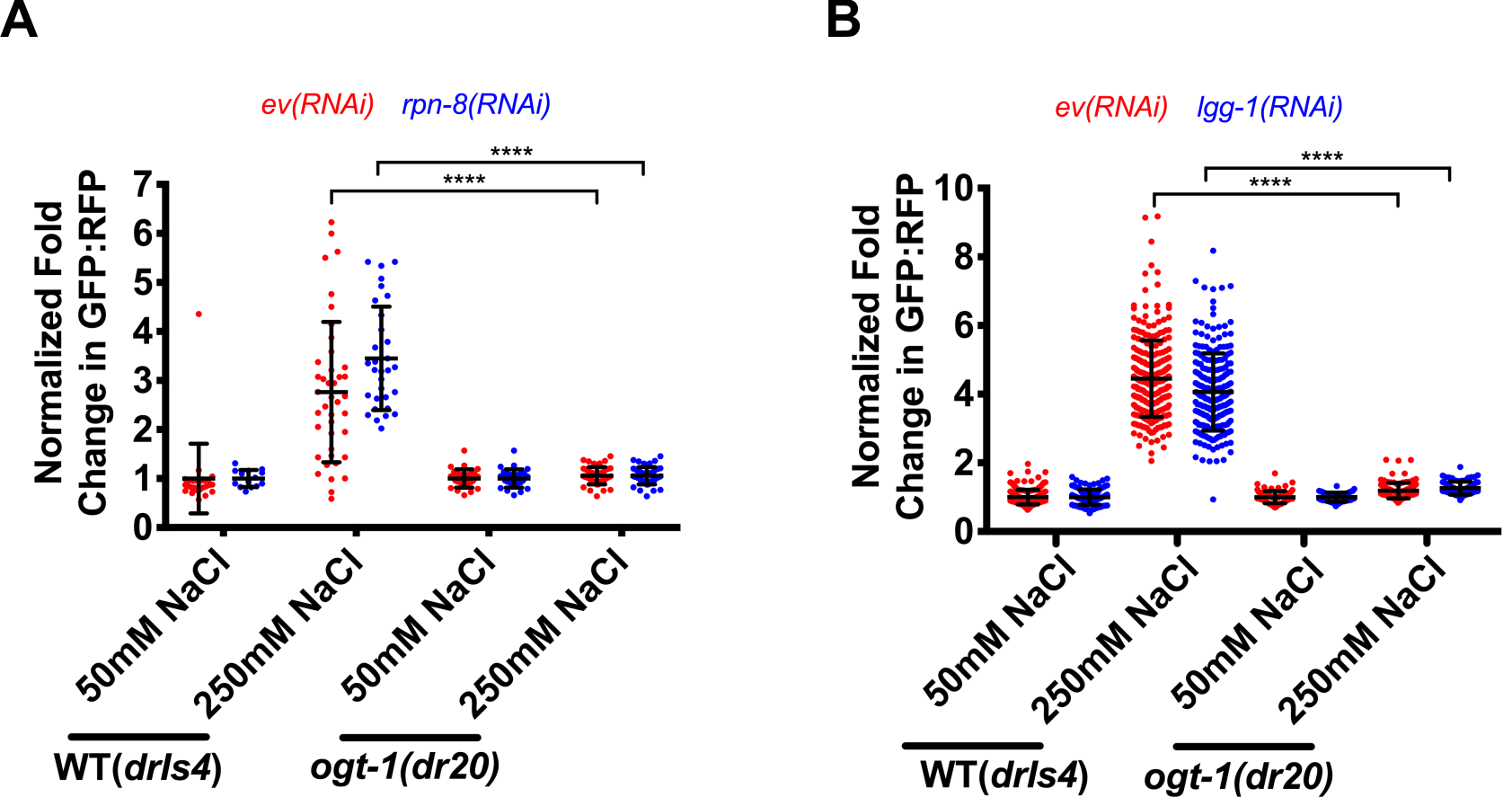

Supplement: S7 Fig — (A) COPAS Biosort quantification of GFP and RFP signal in day 2 adult animals expressing drIs4 exposed to 50 or 250 mM NaCl NGM plates for 18 hours. Animals were placed on empty vector(RNAi) (ev(RNAi)) or rpn-8(RNAi) plates as L1s. Data are represented as normalized fold induction of normalized GFP/RFP ratio on 250 mM NaCl RNAi plates relative to on 50 mM NaCl RNAi plates. Each point represents the quantified signal from a single animal. Data are expressed as mean ± S.D. ****—p<0.0001 (Kruskal-Wallis test with post hoc Dunn’s test). N ≥ 14 for each group. (B) COPAS Biosort quantification of GFP and RFP signal in day 2 adult animals expressing drIs4 exposed to 50 or 250 mM NaCl NGM plates for 18 hours. Animals were placed on empty vector(RNAi) (ev(RNAi)) or lgg-1(RNAi) plates as L1s. Data are represented as normalized fold induction of normalized GFP/RFP ratio on 250 mM NaCl RNAi plates relative to on 50 mM NaCl RNAi plates. Each point represents the quantified signal from a single animal. Data are expressed as mean ± S.D. ****—p<0.0001 (Kruskal-Wallis test with post hoc Dunn’s test). N ≥ 47 for each group. (TIF) [file pgen.1008821.s007.tif]
