# Supplementary material for: The O-GlcNAc transferase OGT is a conserved and essential regulator of the cellular and organismal response to hypertonic stress
Source: PLoS Genet. 2020 Oct 2;16(10):e1008821. doi: 10.1371/journal.pgen.1008821 (PMC7556452; doi:10.1371/journal.pgen.1008821)
Supplement: S1 Table — (PDF) [file pgen.1008821.s008.pdf]

Table S1 - *ogt-1(dr15)* and *ogt-1(dr20)* genetics are consistent with recessive and sin

Strain	Outcross	Number of Nio males from a cross of *ogt-1* x WT	Number of Nio among self progeny of *ogt-1*/+
ogt-1(dr15)	#1	0/30 (0%)	8/27 (30%)
	#2	0/20 (0%)	7/34 (21%)
	#3	0/15 (0%)	13/65 (20%)
ogt-1(dr20)	#1	0/20 (0%)	3/17 (18%)
	#2	*	7/34 (21%)
	#3	0/20 (0%)	17/70 (24%)

* Data not collected

gle gene alleles.
