# Supplementary material for: The O-GlcNAc transferase OGT is a conserved and essential regulator of the cellular and organismal response to hypertonic stress
Source: PLoS Genet. 2020 Oct 2;16(10):e1008821. doi: 10.1371/journal.pgen.1008821 (PMC7556452; doi:10.1371/journal.pgen.1008821)
Supplement: S2 Table — (PDF) [file pgen.1008821.s009.pdf]

Table S2 - All *ogt-1* alleles fail to complement for the Nio phenotype

		Mutant A			
		dr15	*dr20*	*ok430*	*ok1474*
Mutant B	*dr15*	8/18 (44%)	13/31 (42%)	6/12 (67%)	8/20 (40%)
	dr20		11/28 (39%)	9/32 (28%)	12/33 (36%)
	ok430			3/10 (30%)	13/27 (48%)
	ok1474				14/22 (64%)

Number of of animals with a Nio phenotype (no gfp induction on 250 mM NaCl) in the cross progeny of a ogt-1/+ male to an ogt-1/ogt-1 hermaphrodite
Complementation = 0%
Non-compementation = ~50%
