# Supplementary material for: The O-GlcNAc transferase OGT is a conserved and essential regulator of the cellular and organismal response to hypertonic stress
Source: PLoS Genet. 2020 Oct 2;16(10):e1008821. doi: 10.1371/journal.pgen.1008821 (PMC7556452; doi:10.1371/journal.pgen.1008821)
Supplement: S3 Table — (PDF) [file pgen.1008821.s010.pdf]

Table S3 - Backcrossing does not substantially reduce the number of SNPs and INDELS in th[illegible]

Strain	Number of unique non-synonymous SNPs	Number of unique non-synonymous INDELs
ogt-1(dr15)	377	72
ogt-1(dr20)	467	77
ogt-1(dr20) no backcrossing	596	85

e *ogt-1(dr20)* strain.
