# Supplementary material for: The O-GlcNAc transferase OGT is a conserved and essential regulator of the cellular and organismal response to hypertonic stress
Source: PLoS Genet. 2020 Oct 2;16(10):e1008821. doi: 10.1371/journal.pgen.1008821 (PMC7556452; doi:10.1371/journal.pgen.1008821)
Supplement: S4 Table — (PDF) [file pgen.1008821.s011.pdf]

Table S4 - Strain used in this study

Strain	Genotype	Origin
OG119	*drIs4* [*gpdh-1p::GFP; col-12p::dsRed2*]	This study
VP223	*unc-119(ed3);kbIs6* [*gpdh-1p::gpdh-1-GFP*]	This study
OG971	*ogt-1(dr15);drIs4*	This study
OG969	*ogt-1(dr20);drIs4*	This study
RB653	*ogt-1(ok430)*	*C. elegans* Gene Knockout Consortium (Oklahoma Medical Research Foundation, Oklahoma City*)*
OG1034	*ogt-1(ok430);drIs4*	This study
RB1342	*ogt-1(ok1474)*	*C. elegans* Gene Knockout Consortium (Oklahoma Medical Research Foundation, Oklahoma City*)*
OG1035	*ogt-1(ok1474);drIs4*	This study
OG1066	*ogt-1(dr20 dr36);drIs4*	This study
OG1064	*ogt-1(dr34);unc-119(ed3);kbIs6*	This study
OG1115	*gpdh-1(dr81)* [*gpdh1::gfp]*	This study
OG1123	*gpdh-1(dr81);ogt-1(dr84)*	This study
N2 Bristol	WT	*Caenorhabditis* Genetics Center
RB1373	*gpdh-1(ok1558)*	*C. elegans* Gene Knockout Consortium (Oklahoma Medical Research Foundation, Oklahoma City*)*
OG1048	*osm-8(dr9);unc-4(e120);drIs4*	This study
OG1049	*osm-8(dr9); unc-4(e120);ogt-1(dr20);drIs4*	This study
OG1111	*ogt-1(dr20);drIs4;drEx468* [*ogt-1p::ogt-1cDNA::ogt-1 3'utr; rol-6(su1006)*]	This study
OG1119	*ogt-1(dr20);drIs4;drEx469* [*dpy-7p::ogt-1cDNA::ogt-1 3'utr; rol-6(su1006)*]	This study
OG1120	*ogt-1(dr20);drIs4;drEx470* [*nhx-2p::ogt-1cDNA::ogt-1 3'utr; rol-6(su1006)*]	This study
OG1121	*ogt-1(dr20);drIs4;drEx471* [*myo-2p::ogt-1cDNA::ogt-1 3'utr; rol-6(su1006)*]	This study
OG1122	*ogt-1(dr20);drIs4;drEx472* [*rab-3p::ogt-1cDNA::ogt-1 3'utr; rol-6(su1006)*]	This study
OG1125	*ogt-1(dr20);drIs4;drEx473* [*ogt1p::humanOGTisoform1cDNA::ogt-1 3'utr; rol-6(su1006)*]	This study

OG1126	*ogt-1(dr20);drIs4;drEx474* [*ogt1p::humanOGTisoform1H498AcDNA::ogt-1 3'utr; rol-6(su1006)*]	This study
OG1046	*ogt-1(dr20);drIs4;drEx465* [*ogt-1p::ogt-1genomic*]	This study
TJ375	*gpIs1* [*hsp16.2p::GFP*]	Link, C.D., Cypser, J.R., Johnson, C.J., and Johnson, T.E. (1999). Direct observation of stress response in Caenorhabditis elegans using a reporter transgene. Cell Stress Chaperones 4, 235-242.
SJ4005	*zcIs4* [*hsp4::GFP*] V	Calfon, M., Zeng, H., Urano, F., Till, J.H., Hubbard, S.R., Harding, H.P., Clark, S.G., and Ron, D. (2002). IRE1 couples endoplasmic reticulum load to secretory capacity by processing the XBP-1 mRNA. Nature 415, 92-96.
OG1081	*ogt-1(dr50);zcIs4*	This study
MT3643	*osm-11(n1604)*	Culotti, J.G., and Russell, R.L. (1978). Osmotic avoidance defective mutants of the nematode Caenorhabditis elegans. Genetics 90, 243-256.
OG1083	*ogt-1(dr52);osm-11(n1604)*	This study
OG1135	*ogt-1(dr86);drIs4*	This study
OG1140	*ogt-1(dr90);drIs4*	This study
OG1124	*ogt-1(dr84)* [*ogt-1::GFP*]	This study
OG1139	*ogt-1(dr84 dr89)*	This study
OG1141	*ogt-1(dr84 dr91)*	This study
OG1156	*ogt-1(dr93);drIs4*	This study
OG1157	*ogt-1(dr84 dr94)*	This study

IG274	*frIs7 [nlp-29p::GFP + col-12p::dsRed].*	Pujol, N., et al., Distinct innate immune responses to infection and wounding in the C. elegans epidermis. Curr Biol, 2008. 18(7): p. 481-9.
