# Supplementary material for: The O-GlcNAc transferase OGT is a conserved and essential regulator of the cellular and organismal response to hypertonic stress
Source: PLoS Genet. 2020 Oct 2;16(10):e1008821. doi: 10.1371/journal.pgen.1008821 (PMC7556452; doi:10.1371/journal.pgen.1008821)
Supplement: S5 Table — (PDF) [file pgen.1008821.s012.pdf]

Table S5 - DNA oligos used in this study

Primer name	sequence (5' – 3')	target
OG1565	ACGGAAATTGGTACAAATTG TGG	*ogt-1(dr20)*
OG1566	TCCCGGATGCATACTGTAATC	*ogt-1(dr20)*
OG1567	CCAAGCATTTGAACATGAACC	*ogt-1(dr20)*
OG1568	GACAGCTCTCAAGCTGAAACCAG	*ogt-1(dr20)*
OG1569	GATCAGCGTGGATGAGTGGTGTC	*ogt-1(dr20)*
OG1570	TATGATAAACGAGTACGGAAATTGGTACA AATTGT CGAAGATC AGCTTTGCAAGAAACGTCTTCCATCGGTT CATCCA	*ogt-1(dr20)*
OG1571	TATGATAAACGAGTACGGAAATTGGTACA AATTGT CGAAGATT AGCTTTGCAAGAAACGTCTTCCATCGGTT CATCCA	*ogt-1*
OG1544	ACTATCACTTA ATCATACTC TGG	*gpdh-1*
OG1546	ATGAGTAAAGGAGAAGAACT	pPD95.75
OG1547	TTTGTATAGTTCATCCATGC	pPD95.75
OG1548	CTGGAGATCAATGAAATCTCGGAAAAGTT CCCAATCTTCGCGTCAGTGCATAAGGTGT TCACCGGGCACCACGGAGAGCAGGAGCT CTATGATTCCCTGAGAAACCATCCGGAAT ACGACATGAGTAAAGGAGAAGAACT	pPD95.75

OG1549	AAAATTGTACAATATACATACATAAATACCAGTAAAAAAGATTCAAAAACACGTGTTTTTTCTTTATATCCGCTAGATCCATTTCCAGCTCGTCAATAAAACACATAAAACTATCACTTATTTGTATAGTTCATCCATGC	pPD95.75
OG1708	TCAGTGCATAAGGTGTTCACCG	*gpdh-1*
OG1709	CTTTATATCCGCTAGATCCAT	*gpdh-1*
OG1710	CTCGGAAAAGTTCCCAATCTTC	*gpdh-1*
OG1711	CCAGTAAAAAAGATTCAAAAACACG	*gpdh-1*
OG1664	GCTTGTGAATAGATTTTCGA AGG	*ogt-1*
OG1665	ATATCACTAATAATACGGAAACGCCACACGGCTTGATGAGTAAAGGAGAAGAACTTTTC	pPD95.75
OG1666	GAAAATTATACAAAATATACAATTTTTTAAAAATCGTTCGAAAATCTATTCATTTGTATAGTTCATCCATGCCATG	pPD95.75
OG1667	CTCACAGAGGTTCAACATTTTTCC	*ogt-1*
OG1668	GAGCACTGAGGAGTAATACG	*ogt-1*
OG1669	TTTCAGCTTGGCGAACATGCGT	*ogt-1*
OG1670	GGTTTCCAATATGGAAAATC	*ogt-1*
OG1746	CTTGCCAGCATACACAAGGATGC	*ogt-1*
OG1747	ATATGGCAGAAGCTATTCAAAG	*ogt-1*
OG1748	CCAAGCATTTGAACATGAACCTG	*ogt-1*
OG1749	TCGATCAGCGTGGATGAGTGGTG	*ogt-1*

OG1770	GGATAAAGCATACTGTGATG TGG	*ogt-1*
OG1771	ATTGTGGAAGATCAGCTTTGCAAGAAACG TCTTCCCTCAGTTCATCCAGCTCATTCTA TGCTCTACCCGCTTTCACATGCGGCTCG GATTGCAATTGCTGCAAA	*ogt-1*
OG1774	CAGAAGTAGTAAGTGGACCCAAC	*ogt-1*
OG1775	CCAATCAAGCAAATGATCATG	*ogt-1*
OG1776	AAGCCAAAGAATTGATTTCGG	*ogt-1*
OG1777	CCTCTCTCTACACAATACTTTCG	*ogt-1*
OG1772	ATCCACATGTCGAGAGTCGA TGG	*ogt-1*

OG1773	atgctattgtgttttgcaatttcaatcagctttacATGattgatc catctaccttagatatgtggattaaaattctcgagaatgttccg aaatcaattctttg	*ogt-1*
OG1807	tacagaaaaagtcactgaat tgg	*ogt-1*
OG1808	gtcatcaaataatttgtgat tgg	*ogt-1*
OG1806	gaacaattgaaaatataaattggtttacagaaaaa aatgattatgataaacgagtacggaaattggtaca	*ogt-1*
OG1814	actctgaaccgttagtcctc	*ogt-1*
OG1815	ccatcattcacagaaagtgc	*ogt-1*
OG1816	ctttgaaccgccatagaaca	*ogt-1*
OG1817	tccattgcatggaatctgcg	*ogt-1*
OG1818	agctgaaaccagatttcccg	*ogt-1*
OG1487	AAAGTGGGGGGAGTTGAGAGT	*ogt-1*
OG1488	AAAATTTGTGTAGTGTTTT	*ogt-1*
OG1649	TCTAAATACACTCACGTGACGCGTGGATC CC	pPD61.125
OG1650	CTCCCCCCACTTTGGCTAGCCATGGAAC CGG	pPD61.125
OG1651	CCATGGCTAGCCAAAGTGGGGGGAGTTG AG	*ogt-1* promoter
OG1652	ATTGGGCTTCTCCATCGTCTAATCCATTC GATATAATTC	*ogt-1* promoter
OG1653	GGATTAGACGATGGAGAAGCCCAATTAC TTTCAGTC	*ogt-1* cDNA
OG1654	GAAAATCTATTCACAAGCCGTGTGGCGTT TC	*ogt-1* cDNA

OG1655	CCACACGGCTTGTGAATAGATTTTCGAAGGATTTTTAAAAAATTG	*ogt-1* 3’utr
OG1656	CACGCGTCACGTGAGTGTATTTAGACGAGATTC	*ogt-1* 3’utr
OG1680	CAGATAAATGGAGAAGCCCAATTACTTTCAGTCG	*ogt-1p::ogt-1 cDNA::ogt-1 3’utr*
OG1681	TTGGTAGGGAGGCTAGCCATGGAACCGG	*ogt-1p::ogt-1 cDNA::ogt-1 3’utr*
OG1682	ATGGCTAGCCTCCCTACCAATTGAAAATTC	*dpy-7* promoter
OG1683	TGGGCTTCTCCATTTATCTGGAACAAAATGTAAG	*dpy-7* promoter
OG1684	TTAAATCATGGAGAAGCCCAATTACTTTCAGTCG	*ogt-1p::ogt-1 cDNA::ogt-1 3’utr*
OG1685	TTTATTCAACGGCTAGCCATGGAACCGG	*ogt-1p::ogt-1 cDNA::ogt-1 3’utr*
OG1686	ATGGCTAGCCGTTGAATAAACGCTTAGTC	*nhx-2* promoter
OG1687	TGGGCTTCTCCATGATTTAATCACTGAAAATTATTTC	*nhx-2* promoter
OG1692	CCATCTAGAAATGGAGAAGCCCAATTACTTTCAG	*ogt-1p::ogt-1 cDNA::ogt-1 3’utr*
OG1693	cggccagaccGGCTAGCCATGGAACCGG	*ogt-1p::ogt-1 cDNA::ogt-1 3’utr*
OG1694	atggctagccGGTCTGGCCGCAAAAAGG	*myo-3p*
OG1695	GCTTCTCCATTTCTAGATGGATCTAGTGGTCG	*myo-3p*
OG1696	GAAGAAGACCATGGAGAAGCCCAATTACTTTCAG	*ogt-1p::ogt-1 cDNA::ogt-1 3’utr*

OG1697	AAGATGCACTGGCTAGCCATGGAACCGG	*ogt-1p::ogt-1 cDNA::ogt-1 3'utr*
OG1698	ATGGCTAGCCAGTGCATCTTCTTTTGAGAATTC	*rab-3p*
OG1699	GCTTCTCCATGGTCTTCTTCGTTTCCGC	*rab-3p*
OG1700	TTAGACGATGGCGTCTTCCGTGGGCAAC	human *ogt* isoform 1
OG1701	AATCTATTCATGCTGACTCAGTGACTTCAACAG	human *ogt* isoform1
OG1702	TGAGTCAGCATGAATAGATTTTCGAAGGATTTTTAAAAAATTG	*ogt-1p::ogt-1 cDNA::ogt-1 3'utr*
OG1703	CGGAAGACGCCATCGTCTAATCCATTCG	*ogt-1p::ogt-1 cDNA::ogt-1 3'utr*
OG535	CCCAATCCAAGAGAGGTATCCTT	*act-2*
OG536	GAAGCTCGTTGTAGAAAGTGTGATG	*act-2*
OG592	TGCAGAGATTCCAGGAAACCAGG	*gpdh-1*
OG593	CCCTTTTGTAGCTTGCCACGGAG	*gpdh-1*
OG876	CCATTGAAGAGGTAGAAATGC	*hmit-1.1*
OG877	TGTACTTCATTGTGTTGTCC	*hmit-1.1*
OG878	GAGGATATGGAAGAGGATATGG	*nlp-29*
OG879	GTATCCTCCGTACATTCCAC	*nlp-29*
OG1342	TACCTGTTCCATGGCCAACACTTGTC	*GFP*
OG1343	CTTTCCTGTACATAACCTTCGGGC	*GFP*

Purpose
guide RNA for CRISPR/Cas9 conversion of the *dr20* SNP back to WT and the addition of the *dr20* SNP
inner nested *dr20* genotyping forward primer
inner nested *dr20* genotyping reverse primer
outer nested *dr20* genotyping outer nested forward primer
outer nested *dr20* genotyping reverse primer
ssODN repair template for CRISPR/Cas9 conversion of the *dr20* SNP back to WT
ssODN repair template for CRISPR/Cas9 addition of the *dr20* SNP
guide RNA for CRISPR/Cas9 C-terminal addition of GFP
amplification of GFP for CRISPR/Cas9 C-terminal addition of GFP to *gpdh-1*
amplification of GFP for CRISPR/Cas9 C-terminal addition of GFP to *gpdh-1*
forward primer used to amplify GFP for CRISPR/Cas9 C-terminal *gpdh-1* insertion of GFP, includes 120 bp homology overhangs to site of insertion

reverse primer used to amplify GFP for CRISPR/Cas9 C-terminal *gpdh-1* insertion of GFP, includes 120 bp homology overhangs to site of insertion
inner nested *gpdh-1::GFP* genotyping forward primer
inner nested *gpdh-1::GFP* genotyping reverse primer
outer nested *gpdh-1::GFP* genotyping forward primer
outer nested *gpdh-1::GFP* genotyping reverse primer
guide RNA for CRISPR/Cas9 C-terminal addition of GFP
5' Sp9-modified forward primer used to amplify GFP for CRISPR/Cas9 C-terminal *ogt-1* insertion of GFP, includes 35 bp homology overhangs to site of insertion
5' Sp9-modified reverseprimer used to amplify GFP for CRISPR/Cas9 C-terminal *ogt-1* insertion of GFP, includes 35 bp homology overhangs to site of insertion
outer nested *ogt-1::GFP* genotyping forward primer
outer nested *ogt-1::GFP* genotyping reverse primer
inner nested *ogt-1::GFP* genotyping forward primer
inner nested *ogt-1::GFP* genotyping reverse primer
outer nested OGT-1 H612A genotyping forward primer
inner nested OGT-1 H612A genotyping forward primer
inner nested OGT-1 H612A genotyping reverse primer
outer nested OGT-1 H612A genotyping reverse primer

guide RNA for CRISPR/Cas9 conversion of the OGT-1 H612A SNP
ssODN repair template for CRISPR/Cas9 conversion of the OGT-1 H612A SNP
outer nested OGT-1 K957M genotyping forward primer
inner nested OGT-1 K957M genotyping forward primer
inner nested OGT-1 K957M genotyping reverse primer
outer nested OGT-1 K957M genotyping reverse primer
guide RNA for CRISPR/Cas9 conversion of the OGT-1 K957M SNP

ssODN repair template for CRISPR/Cas9 conversion of the OGT-1 K957M SNP
guide RNA #1 for CRISPR/Cas9 deletion of the TPR domain
guide RNA #2 for CRISPR/Cas9 deletion of the TPR domain
ssODN repair template for CRISPR/Cas9 deletion of the TPR domain
inner nested OGT-1 ΔTPR genotyping forward primer
inner nested OGT-1 ΔTPR genotyping reverse primer
outer nested OGT-1 ΔTPR genotyping forward primer
outer nested OGT-1 ΔTPR genotyping reverse primer
internal deletion OGT-1 ΔTPR genotyping forward primer
forward primer for genomic *ogt-1*
reverse primer for genomic *ogt-1*
forward primer for Gibson assembly
Gibson assembly reverse primer
Gibson assembly forward primer for *ogt-1* promoter
Gibson assembly reverse primer for *ogt-1* promoter
Gibson assembly forward primer for *ogt-1* cDNA
Gibson assembly reverse primer for *ogt-1* cDNA

Gibson assembly forward primer for *ogt-1* 3’utr
Gibson assembly reverse primer for *ogt-1* 3’utr
Gibson assembly forward primer for *dpy-7p* backbone
Gibson assembly reverse primer for *dpy-7p* backbone
Gibson assembly forward primer for *dpy-7p*
Gibson assembly reverse primer for *dpy-7p*
Gibson assembly forward primer for *nhx-2p* backbone
Gibson assembly reverse primer for *nhx-2p* backbone
Gibson assembly forward primer for *nhx-2p*
Gibson assembly reverse primer for *nhx-2p*
Gibson assembly forward primer for *myo-3p* backbone
Gibson assembly reverse primer for *myo-3p* backbone
Gibson assembly forward primer for *myo-3p*
Gibson assembly reverse primer for *myo-3p*
Gibson assembly forward primer for *rab-3p* backbone

Gibson assembly reverse primer for *rab-3p* backbone
Gibson assembly forward primer for *rab-3p*
Gibson assembly reverse primer for *rab-3p*
Gibson assembly forward primer for human *ogt-1*
Gibson assembly reverse primer for human *ogt-1*
Gibson assembly forward primer for human *ogt* backbone
Gibson assembly reverse primer for human *ogt* backbone
qPCR forward primer
qPCR reverse primer
qPCR forward primer
qPCR reverse primer
qPCR forward primer
qPCR reverse primer
qPCR forward primer
qPCR reverse primer
qPCR forward primer
qPCR reverse primer
