# Supplementary material for: The O-GlcNAc transferase OGT is a conserved and essential regulator of the cellular and organismal response to hypertonic stress
Source: PLoS Genet. 2020 Oct 2;16(10):e1008821. doi: 10.1371/journal.pgen.1008821 (PMC7556452; doi:10.1371/journal.pgen.1008821)
Supplement: S6 Table — (PDF) [file pgen.1008821.s013.pdf]

Table S6 - Bacterial strains used in this study

**Strain name**	**Genotype**	**Origin**
E. coli OP50		Caenorhabditis Genetics Center
E, coli HT115		Caenorhabditis Genetics Center
