# Supplementary material for: The O-GlcNAc transferase OGT is a conserved and essential regulator of the cellular and organismal response to hypertonic stress
Source: PLoS Genet. 2020 Oct 2;16(10):e1008821. doi: 10.1371/journal.pgen.1008821 (PMC7556452; doi:10.1371/journal.pgen.1008821)
Supplement: S7 Table — (PDF) [file pgen.1008821.s014.pdf]

Table S7 - Chemicals, antibodies, peptides, and recombinant proteins

Reagent	Source	Catalog #
Anti-GFP primary antibody produced in mouse (clones 7.1 and 13.1)	Roche	11814460001
Monoclonal Anti-*ß* -Actin primary antibody produced in mouse (clone AC-15)	Sigma Life Sciences	A1978
Mouse Anti-O-Linked N-Acetylglucosamine (O-GlcNAc) monoclonal antibody (RL2)	Thermo Fisher Scientific Invitrogen	MA1-072
Anti-mouse horseradish peroxidase (HRP) – linked secondary antibody	Cell Signaling Technologies	7076S
Goat anti-Mouse IgG (H+L) Cross-Absorbed Secondary Antibody, DyLight 800	Thermo Fisher Scientific	SA5-10176
Goat anti-Mouse IgG, IgM (H+L) Secondary Antibody, Alexa Fluor 488	Thermo Fisher Scientific	A-10680
MboI Restriction Endonuclease	New England BioLabs	R0147S
DdeI Restriction Endonuclease	New England BioLabs	R0152S
Alt-R S.p. Cas9 Nuclease V3	Integrated DNA Technologies (IDT)	1081058
