# Supplementary material for: The O-GlcNAc transferase OGT is a conserved and essential regulator of the cellular and organismal response to hypertonic stress
Source: PLoS Genet. 2020 Oct 2;16(10):e1008821. doi: 10.1371/journal.pgen.1008821 (PMC7556452; doi:10.1371/journal.pgen.1008821)
Supplement: S8 Table — (PDF) [file pgen.1008821.s015.pdf]

**Table S8 - Critical commercial assays**

| Assay | Source | Catalog # |
|---|---|---|
| RNeasy Mini Kit | Qiagen | 74106 |
| SuperScript VILO Master Mix | Thermo Fisher Scientific | 11755050 |
| SYBER Green PCR Master Mix | Thermo Fisher Scientific | 4344463 |
| NuPAGE LDS Sample Buffer (4X) | Thermo Fisher Scientific | NP0007 |
| NuPAGE Sample Reducing Agent (10X) | Thermo Fisher Scientific | NP0009 |
| Blot 8% Bis-Tris Plus | Thermo Fisher Scientific | NW00082BOX |
| Blot 4-12% Bis-Tris Plus | Thermo Fisher Scientific | NW04122BOX |
| iBlot 2 NC Regular Stacks | Thermo Fisher Scientific | IB23001 |
| iBind Cards | Thermo Fisher Scientific | SLF1010 |
| iBind Solution Kit | Thermo Fisher Scientific | SLF1020 |
| iBind Flex Fluorescent Detection (FD) Solution Kit | Thermo Fisher Scientific | SLF2019 |
| Gentra Puregene Tissue Kit | Qiagen | 158667 |
