# Supplementary material for: The O-GlcNAc transferase OGT is a conserved and essential regulator of the cellular and organismal response to hypertonic stress
Source: PLoS Genet. 2020 Oct 2;16(10):e1008821. doi: 10.1371/journal.pgen.1008821 (PMC7556452; doi:10.1371/journal.pgen.1008821)
Supplement: S9 Table — (PDF) [file pgen.1008821.s016.pdf]

50 mM NaCl		250 mM NaCl	
RFP	GFP	RFP	GFP
390	45	340	542
282	43	469	719
304	34	334	597
453	68	340	745
477	63	317	502
395	55	334	613
372	47	266	514
414	47	417	591
312	37	317	562
422	44	368	517
428	58	592	1009
330	41	431	434
345	44	313	554
436	60	396	657
308	46	380	713
398	51	304	601
275	35	339	603
378	59	347	544
369	50	333	504
321	32	336	578
301	46	285	383
376	42	346	557
391	54	353	726
403	45	291	364
332	42	316	469
385	52	337	606
426	62	310	532
434	54	289	488
323	54	302	543
396	57	262	568
339	54	259	446
307	45	339	493
274	32	278	577
351	42	345	394
466	52	351	593
365	57	387	633
336	48	405	621
355	51	350	535
438	60	351	502
325	46	317	504
370	36	312	533

345	44	292	553
309	45	424	642
336	45	268	467
381	48	358	562
342	44	329	553
307	38	376	548
357	42	295	504
398	49	309	565
273	38	245	473
500	47	292	530
305	41	376	563
350	41	286	614
315	45	369	561
234	35	335	559
339	39	388	473
434	60	275	606
413	47	326	543
364	45	297	573
340	39	335	49
318	52	240	348
330	47	272	375
457	48	322	556
233	24	407	607
324	38	247	503
306	33	292	559
386	36	292	553
339	42	290	466
338	51	292	478
256	43	352	527
218	38	337	582
289	34	268	548
366	45	352	551
298	50	336	581
455	58	314	518
313	54	333	459
329	49	360	545
281	37	305	499
363	49	375	608
311	45	254	521
262	42	344	532
287	39	317	432
329	42	270	559
390	44	234	458

268	39	337	445
228	29	275	512
316	56	215	477
381	50	276	521
255	47	236	501
347	40	296	604
261	42	350	518
276	38	350	486
308	42	286	412
374	53	189	46
355	39	257	526
293	49	301	523
389	58	334	643
368	43	260	536
281	46	343	547
287	49	257	485
298	41	289	523
312	40	358	659
327	50	311	439
393	47	299	501
293	40	276	505
381	50	265	499
306	56	290	415
278	35	309	489
251	29	336	494
383	51	317	488
419	58	245	406
306	41	233	460
291	42	331	511
379	51	278	497
283	43	329	551
310	40	269	423
257	41	306	594
332	42	262	502
221	30	385	559
336	55	313	554
324	49	288	405
320	47	263	442
369	45	259	614
313	36	239	533
377	41	279	567
314	38	264	226
336	47	217	221

376	40	264	497
350	36	178	49
310	34	383	532
312	46	265	479
292	40	308	485
343	42	316	499
245	31	274	440
280	44	337	478
329	41	307	514
241	32	264	477
282	36	328	577
295	41	228	453
377	42	308	544
274	29	347	487
298	44	284	491
168	26	223	449
258	42	277	435
285	41	350	487
280	42	392	563
376	45	312	461
280	34	321	478
357	46	274	502
228	41	233	483
357	46	247	488
310	38	278	498
372	43	279	536
305	33	295	477
324	36	346	474
266	39	324	564
359	46	163	22
426	43	323	422
326	37	316	544
336	44	266	491
281	46	402	617
392	50	305	484
276	29	274	432
297	32	295	495
441	58	288	484
251	37	265	353
372	42	383	38
240	34	303	516
268	43	363	544
328	47	289	519

280	45	294	528
322	44	344	498
305	38	248	541
297	45	295	591
350	45	372	518
355	40	289	294
338	39	324	574
292	53	386	430
317	35	256	376
265	37	277	383
261	41	297	530
403	54	262	480
269	31	242	437
299	43	275	469
233	38	316	530
261	34	261	466
292	40	241	579
292	35	371	546
286	37	302	503
255	38	305	477
250	37	256	466
311	49	257	489
299	38	288	534
353	44	235	386
314	31	270	434
404	45	332	538
409	45	253	551
375	37	270	486
348	42	284	500
257	23	280	468
342	51	246	472
295	31	306	582
282	39	341	526
286	36	325	490
330	38	342	519
320	39	228	463
339	40	292	479
334	40	280	450
276	39	225	410
284	33	256	475
271	40	220	458
275	27	254	301
350	46	271	469

329	43	357	556
336	41	276	431
335	44	261	419
361	46	273	308
316	37	219	394
289	40	203	413
252	35	248	477
255	30	369	531
241	35	315	512
392	42	268	284
400	42	207	314
324	42	323	520
307	41	268	442
209	21	204	499
271	33	233	390
370	51	283	401
268	40	241	298
348	50	286	529
297	35	214	438
289	34	287	462
268	32	250	517
243	35	309	486
202	28	290	474
225	34	318	470
233	27	311	523
377	40	326	558
335	39	216	441
319	43	291	443
313	36	291	466
346	52	225	258
298	38	244	438
270	36	269	431
217	38	255	467
280	46	307	568
380	49	287	486
346	42	261	356
285	38	283	357
285	30	303	520
349	44	311	486
326	41	416	692
239	30	289	516
294	36	290	538
324	39	247	450

343	34	361	618
242	39	243	454
292	41	262	517
261	30	261	474
304	40	305	492
331	39	240	253
337	40	312	476
244	33	333	517
231	28	120	269
261	35	231	470
331	52	127	388
416	42	235	419
262	37	280	452
285	33	306	482
420	51	346	431
262	28	291	532
220	20	368	663
200	29	118	161
266	30	172	42
280	34	350	503
236	26		
414	64		
257	36		
325	31		
345	35		
304	34		
377	48		
186	23		
246	26		
375	50		
167	22		
413	51		
376	50		
344	35		
175	29		
327	40		
251	32		
402	47		
309	33		
427	57		
151	18		
267	39		
332	47		

218	27
259	35
