# Supplementary material for: The O-GlcNAc transferase OGT is a conserved and essential regulator of the cellular and organismal response to hypertonic stress
Source: PLoS Genet. 2020 Oct 2;16(10):e1008821. doi: 10.1371/journal.pgen.1008821 (PMC7556452; doi:10.1371/journal.pgen.1008821)
Supplement: S10 Table — (PDF) [file pgen.1008821.s017.pdf]

50mM NaCl	250mM NaCl
0.88117411	12.17402506
1.164483044	11.70765374
0.854120519	13.65028397
1.146369262	16.73366913
1.008639547	12.09367454
1.063357787	14.01612072
0.964869859	14.75690577
0.866984511	10.82343835
0.905651169	13.53913364
0.796258438	10.72893332
1.034899188	13.01617207
0.948819799	7.689998962
0.973974089	13.51696686
1.050941599	12.67021561
1.140567355	14.32912776
0.978590343	15.09783623
0.971961746	13.5841177
1.191993902	11.97245592
1.034802478	11.558464
0.76130515	13.13718703
1.167092177	10.2628442
0.853051532	12.29399177
1.054704561	15.70636686
0.852749139	9.55261372
0.966106554	11.33442732
1.031469608	13.73271936
1.111465309	13.10580676
0.950206183	12.89542919
1.276747627	13.73114358
1.09924245	16.55620771
1.216488152	13.15070139
1.11940685	11.10608628
0.89189399	15.85056834
0.913810188	8.72149525
0.852179826	12.90212956
1.192602768	12.49126917
1.09097747	11.70982484
1.097123822	11.67345892
1.046142779	10.92220749
1.080906908	12.14185651
0.743044114	13.04627224
0.973974089	14.46292392

1.112161498	11.56333195
1.022791378	13.30748264
0.962121863	11.98856247
0.982517721	12.83639448
0.945276895	11.13029142
0.898452034	13.04735089
0.940214251	13.96380548
1.063003688	14.74378123
0.717863175	13.86139182
1.026591914	11.43495268
0.894601525	16.3951789
1.09097747	11.6104838
1.142262735	12.74326817
0.878574776	9.30986186
1.055784648	16.82882337
0.869083747	12.72026185
0.944115118	14.73370583
0.875990733	1.117030663
1.248791821	11.07342132
1.087671477	10.52873477
0.802119102	13.18659724
0.786627532	11.38959034
0.895679034	15.55195008
0.823581031	14.61984534
0.712244358	14.46292392
0.946157451	12.27161554
1.152304605	12.50140621
1.282750853	11.43356786
1.331192692	13.18884929
0.898452034	15.61563274
0.938956019	11.95426165
1.281349377	13.20537312
0.973487588	12.59835766
1.317538286	10.52645829
1.137402043	11.56133068
1.005562863	12.49437476
1.030868518	12.38186696
1.105009334	15.66454658
1.224226626	11.8104654
1.037759056	10.40730558
0.974916037	15.81109199
0.861592463	14.94732379
1.111331527	10.08425762

0.971352747	14.21841182
1.353364456	16.94313382
1.002210274	14.4159233
1.407574853	16.21211011
0.880327641	15.58328629
1.22891715	11.30252658
1.0514493	10.604301
1.041387585	11.00132525
1.082226313	1.858702356
0.83897704	15.63026865
1.277151099	13.26933062
1.138655148	14.70206464
0.892348419	15.7436441
1.250159236	12.17887094
1.303851122	14.41193972
1.050706489	13.82030628
0.979082344	14.05776276
1.167712888	10.77997995
0.913311927	12.79618055
1.042572326	13.97320781
1.002210274	14.38031812
1.397592053	10.92858465
0.96147295	12.08548828
0.882344328	11.22797646
1.016916336	11.75640074
1.057128527	12.65533865
1.023237038	15.07702769
1.10222466	11.78980788
1.027648962	13.65291589
1.160368263	12.78996991
0.985399005	12.00886352
1.218328925	14.82445856
0.966106554	14.63242301
1.036675424	11.08829828
1.250078351	13.51696686
1.154954543	10.73930947
1.121661211	12.83454103
0.93132223	18.10432882
0.878358857	17.0311169
0.83053192	15.52003433
0.924203844	6.537599837
1.068248772	7.777613573
0.812430031	14.37693415

0.785503778	2.102276809
0.837589154	10.60783315
1.125944696	13.80395266
1.046142779	12.02554711
0.935123545	12.05944399
0.96629433	12.26354236
1.200075217	10.83207897
0.95170375	12.7861138
1.014020553	13.7983855
0.974916037	13.43432317
1.06139164	15.17319981
0.850788796	13.48844871
0.808278928	10.71798903
1.127587452	13.20313226
1.181892259	15.37642236
1.243206884	11.99287507
1.098633452	10.62612055
1.145526343	10.96821992
0.913983784	11.28392402
0.927330849	11.37199568
0.984018894	13.99158696
1.373291815	15.83087908
0.984018894	15.08817424
0.936129055	13.68038654
0.882753275	14.67149629
0.826281297	12.34838566
0.848538032	10.4620325
1.119687403	13.2937625
0.978536895	1.03073945
0.770854973	9.97754627
0.866758174	13.146969
1.00006268	14.0965773
1.250159236	11.72122311
0.974087026	12.11879235
0.802421835	12.04056886
0.822824758	12.81436248
1.004391956	12.83413773
1.12574966	10.17285029
0.862224129	0.757702368
1.081885991	13.00531558
1.225314248	11.44474436
1.09430362	13.71460604
1.227349653	13.71514533

1.043543667	11.05566122
0.951475432	16.65940192
1.157097316	15.29957218
0.981879723	10.63409759
0.860489272	7.768967586
0.88117411	13.52946751
1.386139182	8.50736317
0.84318448	11.21661211
1.066276093	10.55924403
1.199657218	13.62803506
1.023298967	13.99116144
0.880082197	13.7904962
1.098274978	13.02428739
1.245493592	12.80862789
0.994837693	13.63512837
1.046142779	18.34743437
0.915374932	11.23912638
0.987983093	12.71964129
1.138039243	11.94352056
1.130252658	13.90143948
1.203232386	14.53080108
0.970568585	14.15997841
0.951901022	12.54391967
0.753955767	12.27551686
0.850638374	12.37536491
0.840239371	16.63201621
0.753501772	13.74631612
0.921687862	13.44514487
0.683452812	12.76443639
1.138827359	14.65280309
0.80251563	14.52497455
1.05615904	11.77999719
0.961280847	11.51400837
0.87939396	11.58924312
0.930740154	15.50814903
0.901102335	12.52755978
0.914591891	12.27349653
1.079119019	13.91602372
0.887379562	14.16992221
1.127209194	15.89851712
0.749799061	9.049958773
1.003699272	13.2165278
0.998128323	11.89379359

0.931876589	11.92564864
1.003047942	12.25991156
0.973115638	8.615924632
0.89418723	13.73934183
1.057002393	15.53702396
1.06067254	14.68860392
0.898452034	10.98960232
1.109084979	12.41289921
0.818233102	8.09277317
0.80186844	11.58438878
0.989961037	12.29460678
1.019904019	12.59509064
0.76733822	18.6803152
0.929947585	12.78269739
1.052645829	10.82110868
1.139827207	9.443066396
1.097247455	14.12548801
0.899964579	15.63054636
0.898452034	12.29345344
0.911861766	15.79298985
1.099956708	12.01134418
1.058572198	12.48228705
1.154011723	11.28715684
0.884955973	12.84266404
0.810275044	13.0716503
0.889065221	15.59188634
1.029417612	11.62584582
0.878358857	12.22944504
1.147733523	8.756912489
0.973825527	13.70875788
1.018245638	12.23598151
1.337327221	13.98590333
1.25462409	14.12940202
0.984750716	12.9320744
0.927015538	10.41653584
1.018245638	9.633755111
0.803878135	13.10613198
0.962811062	11.93410081
0.96046176	12.70359342
0.95859945	13.63533087
0.935123545	14.16765914
0.919249535	13.91327542
0.757004775	13.07359705

1.230730782	14.26800987
1.072296349	15.0696468
0.877797964	13.86920783
1.004847669	12.31910297
0.899809212	8.050504578
0.906450123	11.6510799
1.032851621	11.85659899
0.925677853	17.11925479
1.024097625	15.53816396
1.199745616	23.33145518
0.771027346	13.61632731
1.078485361	12.32804541
0.884265949	12.02927445
0.927330849	9.512945161
0.816151084	13.96151236
0.69425839	13.75876749
1.107342132	10.41975939
0.861298002	1.864810326
0.927330849	10.97523334
0.841347032	
1.180574653	
1.069752227	
0.728437264	
0.774752116	
0.854120519	
0.972330052	
0.944340713	
0.807145933	
1.018245638	
1.00605108	
0.943048321	
1.015537538	
0.777004302	
1.265533865	
0.93417031	
0.973621327	
0.892864646	
0.815585099	
1.019437963	
0.910352061	
1.115493817	
1.081119239	
0.945847439	

1.032005714
