# Supplementary material for: The O-GlcNAc transferase OGT is a conserved and essential regulator of the cellular and organismal response to hypertonic stress
Source: PLoS Genet. 2020 Oct 2;16(10):e1008821. doi: 10.1371/journal.pgen.1008821 (PMC7556452; doi:10.1371/journal.pgen.1008821)
Supplement: S11 Table — (PDF) [file pgen.1008821.s018.pdf]

50 mM NaCl		250 mM NaCl	
TOF	GFP	TOF	GFP
983	152	995	230
952	102	971	368
927	80	967	199
923	159	961	550
902	69	958	168
900	92	942	427
874	62	941	254
859	89	920	130
856	68	909	110
855	65	907	504
853	62	904	277
844	87	903	149
839	96	902	294
834	72	895	173
833	64	889	218
832	78	883	168
831	73	881	215
831	120	880	140
830	84	874	207
830	144	873	162
829	69	871	188
828	115	867	316
824	54	867	261
821	55	867	255
821	93	857	109
820	88	851	180
816	87	847	186
816	75	844	120
812	77	842	151
809	72	841	220
808	88	839	253
804	106	837	172
797	54	832	167
797	60	831	100
795	47	829	185
795	71	827	151
794	69	827	429
791	73	827	820
791	82	823	596
791	141	823	128
789	72	823	125

786	61	822	771
786	86	821	378
784	78	820	142
783	75	820	157
782	76	818	113
781	69	815	165
781	57	814	132
779	55	813	146
778	82	813	114
778	55	813	302
778	82	813	108
777	57	813	651
775	77	812	372
775	81	810	283
774	67	810	292
773	81	809	99
773	73	808	139
772	68	807	305
772	64	807	125
772	77	806	360
769	84	806	167
769	103	805	125
768	104	803	247
766	81	803	118
766	67	803	126
765	77	802	106
764	81	802	559
764	72	802	132
764	66	801	123
763	50	800	203
762	82	798	107
762	72	798	207
760	67	797	392
760	110	797	146
760	79	795	89
760	82	795	298
758	111	794	110
758	70	789	128
758	74	787	123
756	89	787	89
756	60	786	350
755	70	786	160
754	87	786	215

753	50	786	121
753	89	785	156
752	83	785	121
752	78	785	444
752	71	784	153
750	68	783	336
750	62	783	237
750	74	783	275
750	78	783	103
748	61	783	175
748	83	783	615
746	69	783	683
746	76	781	224
746	66	781	130
745	60	781	134
745	116	780	104
745	84	779	129
744	78	778	174
744	83	778	730
743	73	777	92
743	66	777	128
743	51	776	126
742	58	775	148
741	55	774	104
741	58	774	210
740	52	773	187
740	81	773	406
740	86	773	437
739	70	772	199
737	86	772	222
737	89	772	129
737	65	772	302
736	57	772	132
735	70	771	461
735	75	769	400
735	59	769	309
735	72	768	792
735	67	768	129
735	71	767	121
734	148	767	263
734	69	767	116
733	58	765	162
733	66	765	155

733	60	765	173
732	57	765	161
731	58	763	215
730	82	763	116
730	62	763	237
730	73	762	428
730	58	762	103
730	73	762	138
729	63	761	504
729	63	761	113
729	50	761	175
729	113	761	467
728	47	761	316
726	58	761	240
726	63	761	156
726	71	761	284
726	79	760	143
725	72	760	173
724	62	759	103
724	122	758	119
724	60	758	205
724	51	758	152
723	72	758	298
723	71	757	192
723	81	756	296
721	58	756	119
720	74	755	583
719	61	754	318
719	92	754	120
717	87	754	240
717	63	754	147
716	47	753	111
716	73	753	218
716	59	752	116
715	73	751	161
715	74	751	248
715	47	751	260
712	49	751	146
711	60	751	218
711	61	750	143
710	78	750	213
710	60	749	458
710	75	749	90

709	55	749	113
709	59	748	126
708	58	748	187
707	68	748	362
706	47	747	494
706	120	747	120
706	57	747	122
705	72	747	128
705	72	747	199
704	89	746	106
704	67	746	56
704	74	746	115
702	74	746	73
702	51	745	104
701	50	745	166
701	53	744	131
701	48	744	504
701	78	744	123
700	69	742	119
700	64	742	227
700	73	741	234
700	52	740	205
699	57	740	118
699	64	740	67
698	60	739	166
698	74	739	132
698	77	738	270
698	68	738	227
697	61	738	162
696	96	738	146
696	70	738	185
696	59	737	312
696	60	737	143
696	57	736	130
696	75	736	530
695	68	735	282
694	73	735	114
694	60	735	1025
694	71	733	567
691	72	733	614
689	55	733	314
687	66	733	298
687	68	733	343

687	68	733	355
686	77	732	351
686	53	732	117
685	68	731	117
684	56	731	97
684	52	731	395
683	70	728	806
682	49	728	630
681	66	728	93
680	72	728	250
679	59	727	249
679	40	726	604
679	72	726	150
677	63	724	173
677	57	724	218
676	55	724	200
675	70	724	82
675	69	723	111
674	85	723	124
674	72	720	137
673	68	720	119
673	65	720	82
672	57	720	95
672	58	719	444
671	72	718	178
671	69	718	233
670	58	718	154
669	74	718	132
667	85	718	216
667	74	717	143
665	58	717	112
664	87	717	94
664	71	717	134
663	64	717	94
657	71	716	180
657	57	716	226
656	44	715	412
655	96	715	109
655	50	715	280
653	48	714	155
651	81	714	194
648	59	713	251
648	47	713	120

647	238	713	310
647	57	713	156
643	60	713	165
640	59	713	129
626	56	712	137
623	4	712	418
622	47	712	97
611	49	712	298
599	96	712	582
598	56	712	142
597	108	711	124
594	37	711	115
583	44	711	132
582	99	711	155
570	62	710	62
566	91	710	118
539	73	710	114
524	94	710	133
520	90	710	347
515	97	710	260
		710	168
		709	287
		709	98
		707	178
		707	427
		707	138
		706	102
		706	151
		706	333
		705	124
		705	529
		705	130
		705	220
		704	111
		704	254
		704	349
		704	162
		703	100
		703	134
		702	267
		701	118
		701	115
		701	454

700	288
700	105
700	116
700	481
700	195
699	158
699	204
699	232
699	124
699	182
698	225
698	114
698	107
697	94
697	544
697	86
697	105
697	110
696	91
696	182
696	92
696	195
696	166
696	141
695	122
695	90
695	484
695	848
695	108
694	114
694	275
693	113
693	171
693	143
693	123
693	115
692	130
692	126
692	219
692	90
692	210
691	121
691	458

690	131
690	325
690	111
690	360
690	233
690	126
690	98
690	116
689	237
689	112
688	109
688	412
688	354
688	313
688	143
687	109
686	300
686	307
684	118
684	115
684	251
682	123
682	424
680	147
680	111
680	298
679	149
679	139
679	437
678	338
678	109
677	191
677	116
675	327
675	118
675	202
674	137
673	90
673	193
673	121
672	251
672	103
671	78

671	184
670	108
670	120
670	132
670	393
669	308
669	139
668	114
668	174
668	106
667	88
667	239
666	74
666	136
666	194
665	332
665	105
665	86
665	281
665	102
664	82
663	114
661	216
661	109
661	102
660	232
659	193
659	129
658	191
658	93
658	115
657	113
657	107
657	130
656	185
656	96
655	170
655	396
655	390
653	202
653	93
653	108
653	244

652	287
652	72
652	147
650	184
650	369
648	106
647	49
645	135
645	85
644	113
643	220
639	143
638	104
638	59
635	80
635	106
634	439
634	219
630	736
625	189
622	657
621	91
619	59
619	122
618	60
617	308
613	594
613	340
604	271
601	116
597	157
593	99
592	129
590	548
590	34
580	54
575	88
571	182
540	364
538	389
533	497
532	48
530	110

507	247
500	68
