# Supplementary material for: The O-GlcNAc transferase OGT is a conserved and essential regulator of the cellular and organismal response to hypertonic stress
Source: PLoS Genet. 2020 Oct 2;16(10):e1008821. doi: 10.1371/journal.pgen.1008821 (PMC7556452; doi:10.1371/journal.pgen.1008821)
Supplement: S12 Table — (PDF) [file pgen.1008821.s019.pdf]

50mM NaCl	250mMNaCl
1.555178895	2.324850544
1.077589887	3.811701407
0.86796165	2.069745209
1.732549721	5.756116637
0.769365729	1.763737936
1.028100574	4.558975475
0.713461497	2.714778349
1.042046565	1.421169272
0.798960726	1.217079887
0.76460569	5.588735203
0.731026199	3.081779928
1.036733399	1.659544116
1.150799212	3.278167018
0.868273866	1.944076504
0.772725521	2.466294965
0.942891151	1.913545802
0.883511323	2.454442346
1.452347381	1.600057711
1.017868038	2.382040803
1.744916637	1.866341248
0.83711446	2.17085081
1.39687578	3.665711389
0.659108378	3.027692001
0.673767124	2.958089886
1.139278955	1.279192663
1.079342066	2.127321987
1.072307584	2.208613984
0.92440309	1.429977102
0.953728981	1.803661933
0.895105568	2.630976498
1.095371899	3.032835424
1.325989546	2.066775347
0.681437018	2.018754132
0.757152242	1.210289484
0.594594672	2.24443732
0.898217483	1.836376479
0.874014971	5.217255028
0.928189519	9.972375579
1.042623844	7.283442705
1.792804414	1.564229306
0.917795189	1.527567681
0.780544329	9.433499791

1.100439546	4.630617688
1.000619181	1.741665606
0.963362607	1.925644368
0.977455789	1.389362022
0.888563236	2.036182118
0.734030499	1.630946856
0.710093464	1.80614492
1.060045579	1.41027754
0.711006181	3.735998395
1.060045579	1.336052406
0.737809292	8.053427005
0.999261848	4.607625725
1.051171555	3.513918628
0.870610951	3.625668691
1.053891274	1.230770155
0.949803247	1.730189703
0.885894277	3.801163832
0.833782849	1.557854029
1.00314499	4.49218613
1.098609196	2.083875233
1.347104133	1.561724474
1.361953885	3.093653657
1.063522134	1.477939804
0.879703494	1.578139113
1.012324094	1.329296253
1.066306223	7.010156656
0.947827754	1.655350051
0.868842108	1.544410974
0.659076384	2.55209205
1.082303754	1.348562783
0.950315491	2.608901832
0.886648521	4.946727981
1.455691602	1.842403789
1.045451241	1.125934591
1.085151922	3.769983236
1.472800954	1.393357201
0.928793394	1.631635892
0.981867303	1.571884613
1.184018518	1.137379923
0.798214731	4.478533034
0.932483964	2.047329387
1.160481417	2.751098864
0.667829058	1.548292849

1.188735723	1.998689014
1.110070433	1.550265197
1.043198721	5.688576424
0.949578323	1.962753009
0.911880509	4.315864478
0.831420464	3.044225837
0.992340554	3.532329558
1.045980584	1.32301798
0.82019765	2.247846082
1.116006639	7.899573375
0.93025186	8.773022138
1.024625237	2.884611085
0.889806127	1.674104648
0.810000452	1.72561556
1.566000874	1.341000749
1.134000633	1.665491944
1.054415911	2.249365009
1.122006675	9.436991129
0.988153311	1.190850086
0.893398884	1.656834902
0.690353683	1.633048592
0.786166207	1.920659137
0.746508514	1.351396103
0.78722716	2.728780593
0.706743638	2.433057632
1.100889128	5.282467373
1.168845247	5.685808478
0.952673062	2.592543546
1.173603097	2.892184257
1.21454274	1.680593555
0.887025597	3.934412818
0.778910082	1.719677126
0.957857678	6.013631761
1.026276083	5.231472361
0.807337185	4.041312399
0.98522504	10.37180266
0.916806634	1.689346646
0.971541359	1.586646909
2.027943911	3.44866229
0.945460337	1.521082987
0.795818998	2.129824718
0.905587136	2.037795255
0.823261033	2.274442446

0.783166421	2.116677652
0.797996341	2.83402845
1.129747206	1.52905721
0.854199107	3.124022058
1.005750561	5.649097641
0.799089487	1.359479105
1.005750561	1.821438025
0.869167152	6.660949842
0.869167152	1.493427246
0.6898152	2.312829806
1.558982352	6.17195154
0.649316983	4.176309821
0.803492184	3.171880877
0.872758752	2.06172257
0.98358526	3.753392371
1.094411768	1.892399083
0.998814351	2.289405883
0.861278105	1.364852541
1.694773045	1.578948771
0.83349494	2.720037798
0.708470699	2.016808514
1.001577323	3.954006165
0.987666526	2.550912917
1.126774488	3.937859341
0.809064252	1.583125884
1.033688077	7.766259302
0.853279336	4.241759662
1.286913097	1.600664023
1.22036679	3.201328047
0.883713882	1.960813429
0.660199391	1.482580509
1.025416075	2.911734693
0.828760937	1.551423738
1.026850224	2.156136357
1.040916665	3.321253518
0.661122747	3.481959334
0.692159796	1.955254087
0.848734651	2.91948898
0.862880228	1.91763107
1.104909067	2.856331594
0.849930052	6.149983406
1.062412565	1.208512023
0.780201423	1.517353984

0.836943344	1.694178753
0.823919951	2.514376404
0.967341417	4.86740245
0.669550657	6.651148291
1.709491039	1.615663552
0.812008244	1.642591278
1.02714951	1.723374456
1.02714951	2.679308724
1.271474431	1.429082567
0.957177381	0.754987017
1.057180988	1.550419766
1.060192899	0.984179504
0.730673485	1.404000784
0.717368446	2.241001251
0.760410553	1.770878005
0.688673708	6.813148964
1.119094776	1.662732783
0.991382696	1.612996183
0.91954337	3.076891879
1.048854157	3.176054404
0.747128988	2.78620088
0.820139943	1.603764409
0.920858883	0.910611995
0.86454203	2.259196119
1.066268503	1.796469203
1.109495605	3.679575225
0.979814301	3.0935688
0.880212113	2.207745135
1.387242154	1.989696233
1.011530737	2.521190432
0.852575907	4.257722865
0.867026346	1.951456313
0.823675029	1.776461589
1.083782933	7.242497249
0.984043715	3.858798072
1.05792206	1.559939646
0.869524981	14.02577314
1.028937894	7.779816757
1.047960064	8.424704566
0.802848779	4.308399404
0.966223247	4.088863128
0.995502739	4.706308903
0.995502739	4.870961109

1.128903691	4.822656381
0.777037606	1.607552127
0.998409316	1.609751241
0.823421512	1.334580088
0.76460569	5.434630257
1.030783884	11.1350955
0.722606708	8.703610628
0.974736227	1.284818712
1.064912359	3.453813741
0.873921695	3.444730258
0.592489285	8.367401365
1.066480713	2.077997028
0.935927406	2.403243745
0.846791462	3.02836495
0.818288179	2.778316468
1.043000582	1.139109752
1.028100574	1.544098372
1.268379788	1.724938722
1.074392291	1.913719818
1.016211563	1.662282178
0.9713787	1.145438139
0.853091994	1.327031991
0.86805852	6.21075451
1.079195833	2.493364902
1.03422934	3.263786641
0.87064974	2.15718087
1.11248941	1.849012174
1.28169112	3.025656285
1.115825211	2.005890241
0.877195978	1.571046902
1.317775585	1.318557221
1.075426052	1.879645401
0.97086027	1.318557221
1.086884168	2.528423199
0.87256898	3.174575794
0.674588791	5.795373864
1.474077159	1.533242115
0.76774852	3.938603597
0.739295972	2.183352059
1.251394708	2.732711609
0.915729678	3.540580518
0.729479574	1.692707817
3.699669762	4.372828528

0.886055363	2.200520162
0.93849197	2.327473249
0.927176299	1.819660904
0.899712962	1.935222288
0.064574675	5.904546835
0.759972289	1.370193883
0.806575737	4.209461619
1.611887377	8.221163297
0.941839991	2.005850839
1.819448252	1.754051612
0.626477622	1.626741414
0.759057027	1.867216232
1.710812811	2.192564515
1.093974295	0.878261054
1.617019454	1.671529102
1.362148256	1.614867099
1.804209022	1.884011615
1.740722126	4.9154288
1.8943263	3.683030225
	2.379804145
	4.071232879
	1.390177081
	2.532158415
	6.074335074
	1.963134052
	1.453067383
	2.151109558
	4.743837634
	1.768979711
	7.546695702
	1.854575503
	3.13851239
	1.585771482
	3.62870231
	4.985894118
	2.31436919
	1.430655137
	1.917077884
	3.825290597
	1.692989533
	1.649947426
	6.51370549
	4.137945167

1.508625842
1.666672359
6.910943143
2.801733707
2.273370368
2.93523769
3.338113451
1.784164086
2.618692449
3.242032612
1.642629857
1.54176662
1.356392436
7.849760479
1.240954782
1.51511921
1.587267744
1.314989958
2.629979916
1.329440397
2.817835625
2.398772891
2.037511913
1.765490194
1.302410799
7.004075852
12.27160397
1.562892959
1.652097464
3.98532283
1.639968448
2.481722165
2.075358301
1.785098399
1.668994438
1.889415795
1.831279924
3.182938915
1.308057089
3.052133207
1.761155108
6.666190407
1.909468457

4.737230905
1.617946555
5.247394233
3.396230157
1.836587982
1.428457319
1.690827031
3.459548375
1.634892059
1.593412953
6.022808594
5.174937482
4.575580316
2.090440847
1.595732332
4.39832607
4.500953679
1.735066758
1.690954891
3.69069285
1.813890309
6.252760089
2.174196067
1.641739887
4.407553931
2.207022587
2.058900266
6.472945439
5.013918728
1.616914619
2.837494198
1.723294906
4.87230272
1.758200981
3.00980168
2.044329776
1.344985892
2.884247524
1.80825881
3.756598079
1.5415522
1.16912882
2.757944908

1.62120986
1.801344289
1.981478718
5.899402547
4.630361329
2.089676054
1.716400659
2.619769426
1.59595149
1.326927277
3.603813856
1.117500624
2.05378493
2.929663797
5.021190773
1.588027202
1.300669899
4.249863275
1.542654996
1.242041356
1.729344857
3.286567644
1.658499413
1.551990276
3.53536561
2.945521371
1.96876817
2.919427921
1.421501553
1.757770738
1.729829733
1.637980366
1.990069604
2.836339236
1.47183009
2.610344969
6.08056828
5.988438457
3.111203881
1.432385945
1.663415936
3.758087856
4.427153545

1.110644792
2.26756645
2.847047743
5.709568572
1.645209252
0.761696716
2.105059315
1.325407717
1.764748656
3.441137224
2.250740693
1.639468
0.930082808
1.267087321
1.678890701
6.964108777
3.474122602
11.74972084
3.041389698
10.62344243
1.47380517
0.958631391
1.982254741
0.976456856
5.020602478
9.745772161
5.578388106
4.512556327
1.941215726
2.644938662
1.679077666
2.191584838
9.341547587
0.579585069
0.936388454
1.539235642
3.205719828
6.779503784
7.272062609
9.378199419
0.907444116
2.087406826
4.899810427

1.367820764
