# Supplementary material for: The O-GlcNAc transferase OGT is a conserved and essential regulator of the cellular and organismal response to hypertonic stress
Source: PLoS Genet. 2020 Oct 2;16(10):e1008821. doi: 10.1371/journal.pgen.1008821 (PMC7556452; doi:10.1371/journal.pgen.1008821)
Supplement: S13 Table — (PDF) [file pgen.1008821.s020.pdf]

WT	dr15	dr20	ok430	ok1474
1.032768863	0.219894853	0.299428326	0.226631986	0.343054718
0.866534878	0.181284119	0.247101046	0.465932462	0.249126815
0.796211839	0.21614395	0.218030334	0.405140983	0.278605522
0.995957348	0.254676594	0.273264686	0.407435045	0.285624747
1.289559158	0.181810252	0.305242468	0.462385812	0.28876348
1.003669972	0.226109273	0.276171757	0.318879631	0.354232158
0.818272578	0.228274478	0.247101046	0.471243609	0.260867986
0.884569512	0.176630051	0.255822259	0.522962595	0.42961092
1.151245071	0.161979597	0.290707113	0.545288561	0.295697473
1.079262374	0.252333417	0.235472761	0.543235936	0.328343678
1.141669944	0.259750075	0.177331339	0.474812586	0.332898123
1.058849948	0.218735311	0.247101046	0.393382424	0.317513027
1.03501418	0.242825307	0.223844477	0.128356878	0.369000922
0.827756508	0.216149649	0.247101046	0.459957145	0.234060912
0.612782222	0.223930396	0.238379832	0.472363642	0.315036305
0.598288186	0.237151819	0.287800041	0.371224557	0.279088711
0.96042444	0.255019996	0.28489297	0.333574162	0.520412028
1.134835376	0.340040302	0.273264686	0.58053202	0.236512799
0.915461405	0.215592562	0.287800041	0.310512171	0.330340647
0.856455114	0.242741255	0.212216192	0.534331347	0.318759593
0.858082057	0.247779621	0.270357615	0.289119126	0.327145001
0.886862449	0.215592562	0.296521255	0.48575577	0.351015028
0.997449209	0.181825053	0.209309121	0.38820129	0.353719304
0.98816233	0.25479121	0.244193975	0.536502907	0.308727186
0.850040723	0.226574489	0.215123263	0.410300827	0.280290409
0.85739313	0.305422797	0.235472761	0.50980951	0.314323199
1.301123815	0.283783181	0.267450544	0.384973615	0.293987953
0.869266464	0.240555912	0.28489297	0.493452217	0.34612784
0.880421317	0.217861958	0.209309121	0.318325058	0.406589419
0.880919216	0.244472563	0.28489297	0.452737914	0.353142391
1.162823084	0.276183936	0.276171757	0.236575188	0.258759053
0.783446309	0.193289883	0.270357615	0.430634548	0.265596182
0.882391432	0.213638551	0.287800041	0.719673296	0.276836293
1.132137975	0.321870586	0.209309121	0.349183099	0.315881292
1.008206595	0.208637964	0.25872933	0.743097202	0.253464926
1.030394681	0.2378186	0.279078828	0.329095455	0.398605623
0.817199969	0.318184885	0.215123263	0.378958402	0.301967473
1.070610121	0.262018099	0.28489297	0.635520302	0.291194031
0.949051873	0.223147019	0.261636401	0.15741349	0.21838719
0.986606311	0.266759864	0.218030334	0.418551071	0.259991757
1.006711397	0.282395891	0.337220251	0.498059614	0.28059195
0.915310765	0.360992197	0.281985899	0.50527787	0.326004129

1.03604395	0.212309427	0.209309121	0.558286932	0.341078837
1.659244288	0.167826546	0.218030334	0.587074902	0.382156558
1.037273151	0.255517111	0.247101046	0.508148767	0.367908799
1.012550823	0.214236634	0.238379832	0.341062562	0.41082709
1.080701076	0.21239386	0.20640205	0.201613337	0.56882245
1.085464376	0.242238834	0.197680837	0.415049679	0.306590666
1.129657244	0.166457513	0.168610125	0.573750104	0.373106882
0.972524578	0.255605586	0.218030334	0.11760778	0.337505491
0.810520086	0.206841573	0.270357615	0.337989926	0.305591999
0.826026242	0.283408063	0.261636401	0.588313044	0.279981476
0.96982194	0.275224548	0.270357615	0.532264301	0.736033001
0.757131227	0.197677994	0.281985899	0.969335703	0.253378555
0.938484267	0.183377582	0.261636401	0.306343619	0.310651469
0.72508865	0.222622755	0.270357615	0.378958402	0.298240041
1.266301665	0.249962391	0.273264686	0.250497927	0.327798441
1.090570594	0.227352157	0.209309121	0.378958402	0.285974606
0.915869237	0.229237661	0.235472761	0.440080725	0.365519781
1.054384071	0.20170694	0.287800041	0.430634548	0.287496105
0.939333351	0.191637833	0.273264686	0.414485752	0.212841957
1.027752429	0.229208935	0.212216192	0.346256408	0.248192444
0.930081523	0.22897417	0.226751548	0.400102004	
1.118631343	0.214945137	0.279078828	0.40960945	
0.947534627	0.212223929	0.241286903	0.555218124	
0.871372866	0.170010135	0.270357615	0.843488057	
0.777810057	0.240868932	0.229658619	0.321855081	
1.28168223	0.270140078	0.229658619	0.348813984	
0.935294049	0.200910632	0.197680837	0.709243248	
0.999207198	0.236239511	0.299428326	0.413409166	
1.091493811	0.232787061	0.247101046	0.185360088	
1.347513315	0.220414121	0.209309121	0.364302552	
0.76741131	0.223930396	0.226751548	0.395677155	
0.912732131	0.184793625	0.183145481	0.597520458	
1.11372115	0.161968016	0.247101046	0.646137707	
0.819087874	0.174629975	0.267450544	0.376232083	
0.874907196	0.22212567	0.250008117	0.223794332	
0.931457393	0.178649591	0.261636401	0.409275074	
0.903265532	0.271705695	0.226751548	0.205269135	
1.092804437	0.183253678	0.261636401	0.196983328	
0.971546429	0.170438958	0.250008117	0.580958255	
1.272954146	0.25785157	0.276171757	0.378958402	
0.817459873	0.266857243	0.220937406	0.367812567	
1.102033641	0.196602544	0.267450544	0.319582431	
1.004936631	0.2628176	0.255822259	0.476127223	

1.039852525	0.243232634	0.223844477	0.462798757
0.835940741	0.201429547	0.264543472	0.273287309
0.925051577	0.220124538	0.287800041	0.392738708
0.899108873	0.163593272	0.267450544	0.558963643
0.873325085	0.17247405	0.273264686	0.525804783
0.744512373	0.249490764	0.261636401	0.304520145
0.991969706	0.266857243	0.215123263	0.410300827
1.175470552	0.190428762	0.241286903	0.296576141
1.100398741	0.157701967	0.200587908	0.315798669
0.753195215	0.200697076	0.20640205	0.564722325
1.070134421	0.189777272	0.334313179	0.524711634
0.761013951	0.239396158	0.215123263	0.275243471
0.984970148	0.252401049	0.197680837	0.489603191
0.869127462	0.210157456	0.209309121	0.378958402
1.00137746	0.265344692	0.218030334	0.412750871
1.050440047	0.232082645	0.23256569	0.301619953
1.086661594	0.244067052	0.244193975	0.543481806
1.048338412	0.26331916	0.15407477	0.135342287
1.266474053	0.241702388	0.250008117	0.41725739
0.98940235	0.296815807	0.223844477	0.441029175
1.035129562	0.237772867	0.261636401	0.564226954
0.953356321	0.207073707	0.235472761	0.402643302
1.027768679	0.238663353	0.194773765	0.419992115
1.307004219	0.222859727	0.235472761	0.371670741
2.033982362	0.262304284	0.223844477	0.319122865
1.245835592	0.234372402	0.305242468	0.442496338
1.006547225	0.211192714	0.165703054	0.396833799
0.989720598	0.228920103	0.287800041	0.548280242
0.975128763	0.17423849	0.250008117	0.422804829
0.999207198	0.22935379	0.244193975	0.441175453
0.899489076	0.209471638	0.25872933	0.25538501
1.093415486	0.273355098	0.244193975	0.194465496
1.276954333	0.258178747	0.218030334	0.371454275
1.000517313	0.188759153	0.299428326	0.263422304
0.882622427	0.226939539	0.244193975	0.220587727
0.999893938	0.202284379	0.194773765	0.223125041
0.975446653	0.222372203	0.188959623	0.533635301
1.12488068	0.276881418	0.183145481	0.490416756
0.967069137	0.20429728	0.276171757	0.476754119
1.401903534	0.236125187	0.215123263	0.557291768
1.140613277	0.234940613	0.197680837	0.378958402
1.178446427	0.199391965	0.267450544	0.718026446
1.204495523	0.185609491	0.183145481	0.213698347

1.101766694	0.167961647	0.229658619	0.625281364
1.271824553	0.246029159	0.235472761	0.673703826
0.995040084	0.249962391	0.215123263	0.715131178
0.923455765	0.204111302	0.200587908	0.307263569
0.933453617	0.214018894	0.325591966	0.476754119
1.156023556	0.199459786	0.305242468	0.484843838
0.758999394	0.272901978	0.235472761	0.39461784
0.988233367	0.207553518	0.354662677	0.330308337
1.175875271	0.198783651	0.31105661	0.7171059
0.8022892	0.23845844	0.215123263	0.449462291
0.827357892	0.212963385	0.194773765	0.580794943
0.701175624	0.213170174	0.244193975	0.535955455
0.94459007	0.239986828	0.197680837	0.32742006
1.220209551	0.199480663	0.212216192	0.465577466
1.208609663	0.179086473	0.197680837	0.178517594
1.144615429	0.224490033	0.226751548	0.277286636
1.12488068	0.23973227	0.223844477	0.382403479
1.049401673	0.266676744	0.252915188	0.781601705
1.042221716	0.179555096	0.226751548	0.496090999
0.892188321	0.256380344	0.264543472	0.378958402
0.99295023	0.245556274	0.23256569	0.364772794
1.115671131	0.167895093	0.247101046	0.211342186
0.951241381	0.205440004	0.264543472	1.057558332
0.858399513	0.293989858	0.226751548	0.419926878
1.189632848	0.195429229	0.203494979	0.416479036
1.053390209	0.25017567	0.212216192	0.613020945
0.716104412	0.30472447	0.200587908	0.196012967
1.211409963	0.199008519	0.226751548	0.475901249
1.477116045	0.198606482	0.238379832	0.271299765
0.633833696	0.195023661	0.20640205	0.326851622
1.239664423	0.215592562	0.247101046	0.279232507
0.984022606	0.288562353	0.145353556	0.578410193
0.578464281	0.195358956	0.279078828	0.578072139
0.771622332	0.214846567	0.165703054	0.132635441
0.909567385	0.2303805	0.264543472	0.548836307
1.043023166	0.223798508	0.203494979	0.194337642
0.769298243	0.295397544	0.212216192	0.322114642
0.751781298	0.253638309	0.191866694	0.277902828
0.921182564	0.168026295	0.220937406	0.113687521
1.217870816	0.143728375	0.212216192	0.643964278
1.196361392	0.224966152	0.223844477	0.366430852
1.29629107	0.17709389	0.203494979	0.384788532
1.17088807	0.210287153	0.194773765	0.160123269

0.752165724	0.167748733	0.229658619	0.503975607
0.943320991	0.182084041	0.218030334	0.568437603
1.068636646	0.185155965	0.226751548	0.321540463
0.67207404	0.17247405	0.241286903	0.340232726
1.035101471	0.255394266	0.247101046	0.367298144
0.848347513	0.306002347	0.220937406	0.456409755
1.051268857	0.264317816	0.191866694	0.582301935
1.202078374	0.219331162	0.215123263	0.624656707
0.943448312	0.222925643	0.296521255	
0.859422113	0.227112776	0.188959623	0.467382029
0.920470924	0.207300541	0.273264686	0.655889542
1.098673783	0.246021543	0.18023841	0.485540453
1.348501538	0.166819163	0.218030334	0.55171885
0.943120299	0.192977958	0.165703054	0.169121932
0.820154695	0.185573345	0.200587908	0.251170104
0.975773689	0.258711075	0.226751548	0.468125085
0.744446582	0.286278648	0.209309121	0.258737116
0.982815004	0.228859797	0.197680837	0.332743963
1.324035379	0.254244615	0.20640205	0.241460221
0.927694512	0.203044847	0.229658619	0.474995806
0.951911412	0.203296409	0.203494979	0.327944771
1.108045731	0.181889507	0.218030334	0.660616674
1.115204287	0.181881725	0.255822259	
0.783469526	0.243288332	0.203494979	
1.424218676	0.156304608	0.293614184	
0.637563034	0.188302365	0.174424268	
0.831108423	0.316313412	0.241286903	
0.90108243	0.217511784	0.261636401	
0.809648631	0.179972922	0.287800041	
0.909195594	0.17171924	0.220937406	
1.072671028	0.18402043	0.357569748	
1.003452279	0.21763287	0.247101046	
0.980817014	0.265072823	0.25872933	
0.830719964	0.206101066	0.197680837	
0.952047451	0.17438512	0.209309121	
1.142478092	0.243629263	0.171517196	
0.717537525	0.203509203	0.200587908	
0.963108542	0.19466597	0.255822259	
1.137810343	0.276730752	0.188959623	
0.929653455	0.22739116	0.226751548	
1.126224624	0.25290666	0.264543472	
1.082811972	0.212717995	0.159888912	
1.146680693	0.22482026	0.276171757	

1.020804267	0.257470254	0.220937406
0.82948761	0.220529797	0.212216192
0.96965521	0.269490703	0.168610125
0.854027058	0.230633904	0.244193975
0.881372027	0.202910647	0.215123263
0.82267393	0.235191886	0.252915188
1.151215679	0.170766386	0.20640205
1.071314934	0.177332474	0.244193975
0.936830718	0.184793625	0.209309121
0.794334877	0.245261264	0.197680837
0.97957089	0.250778292	0.191866694
1.107131084	0.168871459	0.218030334
0.893905181	0.209992756	0.194773765
1.111245763	0.201043371	0.244193975
1.067454339	0.201904146	0.255822259
1.042300154	0.116977668	0.18023841
1.820195275	0.154374921	0.212216192
1.173171012	0.302525047	0.191866694
0.694122801	0.239365126	0.247101046
0.93298927	0.21491246	0.220937406
1.054201276	0.226939539	0.133725272
1.177585933	0.257756421	0.226751548
0.761905484	0.248479563	0.235472761
0.876440775	0.208312813	0.177331339
1.206972564	0.306108524	0.23256569
0.96163269	0.161012167	0.267450544
1.12488068	0.259672826	0.188959623
1.22164461	0.21399558	0.168610125
1.062013097	0.213025984	0.267450544
1.07259539	0.212275754	0.188959623
1.072200318	0.195715234	0.220937406
1.282042227	0.207487579	0.203494979
1.138011583	0.204989649	0.191866694
0.757254639	0.236729088	0.215123263
1.232477963	0.244126872	0.223844477
0.910265287	0.266122069	0.25872933
0.9923366	0.355649044	0.165703054
0.941389505	0.230159627	0.203494979
0.976311534	0.223130764	0.215123263
1.229520743	0.248543391	0.171517196
0.999893938	0.284538407	0.215123263
1.04733933	0.189721455	0.165703054
1.114925984	0.188556839	0.156981841

1.118881317	0.216494623	0.177331339
0.759353415	0.235678826	0.130818201
1.039544904	0.22197104	0.197680837
0.911835097	0.235971959	0.273264686
0.804370123	0.211005487	0.229658619
1.034792833	0.192401331	0.244193975
0.803181849	0.30436597	0.203494979
1.060232365	0.181551631	0.20640205
0.849009042	0.236050251	0.226751548
1.190447496	0.184245276	0.183145481
1.000844407	0.297119162	0.252915188
0.993137898	0.196381344	0.293614184
1.163374696	0.268545121	0.191866694
1.036802775	0.236456359	0.20640205
0.986895317	0.341157461	0.212216192
0.979487939	0.243902697	0.165703054
0.98412274	0.205184646	0.203494979
0.904509319	0.198273492	0.212216192
0.966167357	0.229437956	0.203494979
0.958876617	0.204245585	0.203494979
1.015742401	0.252257964	0.25872933
1.014743848	0.233863118	0.200587908
1.171408592	0.158564852	0.174424268
0.813550068	0.227569927	0.244193975
0.861750697	0.231150788	0.188959623
0.909607019	0.185948585	0.177331339
0.799257325	0.234886838	0.162795983
1.022069005	0.225116732	0.18023841
1.28557792	0.254288663	0.194773765
0.917313412	0.236675747	0.165703054
0.902159944	0.195617533	0.194773765
0.832760504	0.266019297	0.264543472
1.202944072	0.192690788	0.191866694
0.886480042	0.214740418	0.241286903
0.795793247	0.183106012	0.177331339
1.262656763	0.209059454	0.142446485
0.708013134	0.177622529	0.191866694
0.953232221	0.209059454	0.168610125
1.112276975	0.264274754	0.223844477
0.94739167	0.216525863	0.145353556
0.710776254	0.209674335	0.165703054
1.027257899	0.200193094	0.191866694
0.741340128	0.249911378	0.200587908

1.279626171	0.183828141	0.252915188
1.034373039	0.216243899	0.148260627
1.057083444	0.171507809	0.162795983
1.302087911	0.197859841	0.194773765
1.013091296	0.214041537	0.212216192
0.985332376	0.167926053	0.220937406
0.835871222	0.199406935	0.156981841
1.345350856	0.206027022	0.151167699
1.18598531	0.214825329	0.162795983
0.837801757	0.188840201	0.148260627
0.985876733	0.258711075	0.12791113
0.830766896	0.222169963	0.159888912
0.998414805	0.230430917	0.156981841
0.952676724	0.16669528	0.098840418
0.872729726	0.207443015	0.20640205
1.070061934	0.19547059	0.119189916
0.903805864	0.333655156	0.133725272
0.702306456	0.224670144	0.281985899
0.868436492	0.183622563	0.165703054
0.885065129	0.195093597	0.159888912
1.275210356	0.175127497	0.090119205
1.139988968	0.2054745	0.191866694
0.966113737	0.236987244	0.171517196
1.471962823	0.213867822	0.145353556
0.744206774	0.217500461	0.162795983
0.841050028	0.232703083	0.159888912
0.953387243	0.142540537	0.156981841
1.057083444	0.274609521	0.122096987
1.131850201	0.240905049	0.125004058
0.877406931	0.230775137	0.305242468
0.988659175	0.203519379	0.122096987
0.933280125	0.246934212	0.267450544
1.297072716	0.176450397	0.156981841
0.944344275	0.234178128	0.075583849
0.932674177	0.276152271	0.183145481
0.913335955	0.225132056	0.197680837
1.00969682	0.215592562	0.040698996
1.162198997	0.180496099	
0.811481088	0.253431257	
1.102985192	0.267632146	
0.909531901	0.186226134	
0.785443001	0.245468685	
0.845043107	0.201996635	

1.120251542	0.182771008
0.851261055	0.217238307
1.001759412	0.179813371
0.831482799	0.171996282
0.977396324	0.25446991
0.974896589	0.150572583
1.003377889	0.211810237
1.22108758	0.245296426
0.916316177	0.168977954
1.025401436	0.181384148
0.963380957	0.210007263
1.163141928	0.267105829
0.856266141	0.249813604
1.096657867	0.164118906
0.95803235	0.199459786
0.960052406	0.234178128
1.042772601	0.260545905
1.169146818	0.207443015
0.968124733	0.228491263
1.020439704	0.238029797
0.910617693	0.200551221
0.921777224	0.243792433
0.91564873	0.220937832
1.079717404	0.231639905
0.887564081	0.199887144
1.184587723	0.261324318
0.955065866	0.181551631
0.874180529	0.180861846
0.760672001	0.244338237
1.08533942	0.267334777
0.752087854	0.186901537
0.699364018	0.160185822
1.094839356	0.28745675
1.134776991	0.219185772
1.038533372	0.295849648
0.96964237	0.257374067
0.54029702	0.244870565
0.782777793	0.301210661
0.963806217	0.194845161
0.86618719	0.243629263
1.061542804	0.212512669
0.786771581	0.161045047
0.99413797	0.275529084

0.909217282	0.198749393
1.370999948	0.222448863
0.687341592	0.086237025
1.264328698	0.20338921
1.180430343	0.210939485
1.269602171	0.261714354
1.304512789	0.266924125
0.700879334	0.273929373
1.059670206	0.226665292
0.8426304	0.222661171
0.766523858	0.208687462
0.859160834	0.306002347
0.961620486	0.20828434
1.300320786	0.244338237
0.986977375	0.190852432
0.968647252	0.206609539
0.935413145	0.261198681
0.849909847	0.228160739
1.363491734	0.186458432
0.931273766	0.17247405
0.744807359	0.212692214
0.708354424	0.232176606
1.065141254	0.274880517
0.884688767	0.291747149
1.15787718	0.210434846
1.179167851	0.197761599
0.742326322	0.251524656
1.231753402	0.148954861
1.27456836	0.233811652
0.911519474	0.288872793
1.098431204	0.259441897
1.130685018	0.191236917
1.101542907	0.211192714
1.000802932	0.202118027
0.895136264	0.218909371
1.163242948	0.174448944
0.847949271	0.257905121
1.063716955	0.214726729
0.955942556	0.332548005
0.889698526	0.306719315
0.821830634	0.232962579
0.658119846	0.190393432
1.386013695	0.216365296

1.104049556	0.198282211
1.079885453	0.213400096
1.033921742	0.17247405
1.008513713	0.27274966
0.838668436	0.37944291
0.688756729	0.193067966
1.127929137	0.223201712
0.867832474	0.322613331
1.0944785	0.176117868
0.803806953	0.215592562
1.185685041	0.184793625
1.109547734	0.228175786
0.694141246	0.405821294
0.912403218	0.17799813
0.892347206	0.1805685
1.237368748	0.253321261
0.770579419	0.220259068
0.693322683	0.13541351
0.950260214	0.189649889
0.985744495	0.256876244
1.060556959	0.247583717
1.129398273	0.216915216
1.177710128	0.258711075
1.160722467	0.194728766
1.05818815	0.217059178
1.022391552	0.311780013
1.164350178	0.4106525
0.766152498	0.236848167
1.295979036	0.204776882
1.017559909	0.164435005
1.077046248	0.183128817
0.964641267	0.239746086
0.769211012	0.215592562
0.917150102	0.203176237
1.004843908	0.175035546
1.086841237	0.165386075
1.210160443	0.20708233
0.991983385	0.258711075
1.108578062	0.230322489
1.051518897	
1.006398824	
1.038351397	
1.16835433	

1.034164496
0.740117572
1.223084549
0.893944911
0.780633919
0.849755065
0.881850904
1.224553652
1.12488068
0.747213159
0.988906092
0.960708905
0.897792092
1.129398273
0.997394203
1.0649912
0.864194046
1.282247407
0.96418344
1.142283897
1.413566872
1.439309211
0.855999575
0.91760453
1.136417918
0.760633603
1.086024698
0.781023145
1.177445198
1.01997491
1.332760184
0.866144288
1.107178695
1.138325469
1.14625147
1.373495613
1.313991055
0.915065391
1.031682474
1.368518431
1.33702923
0.876562649
0.982290171

0.956898499
1.051359067
1.042746535
0.902446647
1.039523881
0.86663959
1.081017408
1.054083994
0.963727561
0.92623404
0.976041506
1.010654386
1.120667644
0.111595306
0.872247903
0.973817567
0.943865398
0.875351988
1.060601784
1.004455498
1.151764621
1.12488068
1.049349124
1.046457626
0.999893938
1.138871733
0.766626602
1.022352493
1.065062123
0.776025279
1.001623858
1.067634844
0.929456494
1.048801794
0.675854236
1.239451861
0.956148578
1.126562116
0.902682027
0.963312452
1.047357258
0.977468876
0.965850856

1.035101471
0.967998211
1.570145949
1.051805364
1.025906407
0.859385966
0.715961791
1.244337036
0.930158846
0.67904885
0.270913672
1.042529602
1.049184484
0.115372377
0.914537138
0.902682027
1.090162141
0.911797556
1.113852438
1.499840907
1.751722586
1.273903334
1.261229854
0.943120299
0.865024523
0.972984611
