# Supplementary material for: The O-GlcNAc transferase OGT is a conserved and essential regulator of the cellular and organismal response to hypertonic stress
Source: PLoS Genet. 2020 Oct 2;16(10):e1008821. doi: 10.1371/journal.pgen.1008821 (PMC7556452; doi:10.1371/journal.pgen.1008821)
Supplement: S14 Table — (PDF) [file pgen.1008821.s021.pdf]

WT	ogt-1(dr20)	ogt-1(dr20 dr36)
1.064181663	0.245240481	1.038553647
0.951951413	0.234603234	0.955371429
0.671241578	0.284670483	0.79838268
1.168109779	0.218707723	0.653084277
0.732921307	0.263596652	0.712700962
1.02634713	0.224586841	1.142427089
0.864198829	0.2552472	0.772301565
0.951119208	0.318628234	0.638249516
0.977463257	0.233116041	0.910900234
1.294617992	0.261364194	0.547255898
0.997371285	0.290199097	0.6606727
1.134072843	0.224969768	1.04024416
1.154640522	0.309460021	0.843306137
0.708505013	0.210617489	0.714065907
0.93136509	0.28662329	0.972102219
0.992869679	0.327705962	0.689990477
0.827753637	0.317415328	1.130627412
1.006478685	0.247574386	1.19243567
1.130372634	0.280274836	0.952940629
1.079898282	0.266299719	0.754528221
1.135867811	0.261497117	0.727099208
1.154640522	0.22038705	0.76061686
0.750247899	0.210805005	0.982387714
0.731881642	0.170114126	0.832604863
0.565084046	0.206972186	0.683273164
1.102222254	0.215098115	0.888354579
1.066682376	0.248597877	0.683265993
0.973720794	0.340276306	0.818603614
0.911961651	0.289869423	0.794779111
1.069842855	0.269803029	0.670491786
0.914487623	0.234800379	0.59508857
1.567411494	0.245182436	0.715731705
0.993756201	0.266686851	0.591835732
1.074374557	0.326591093	0.913005256
1.04617912	0.24552133	0.751370276
0.825739719	0.295674552	0.890478276
1.036046967	0.255872208	0.770896866
0.927101822	0.291144719	0.929462065
1.078024081	0.279947182	0.375943341
0.903245102	0.259338643	0.794470289
1.022268127	0.216425243	0.773733268
1.154039459	0.277194892	0.692723862

0.831029318	0.27944316	0.640524779
1.217882014	0.303559207	0.713459541
1.107671802	0.21620334	0.807419614
1.05013306	0.265968931	0.976459737
1.03045182	0.22965407	0.65581723
1.242422926	0.273016075	1.022413002
0.890706163	0.279122796	0.730218162
0.834299145	0.216658077	0.700031502
1.079950367	0.216524749	0.750196626
1.077948946	0.254474	1.302525289
1.074102917	0.263838307	0.639535267
0.863265504	0.199246309	0.662832673
1.043604294	0.183975277	0.630088188
0.946327436	0.26753507	0.888587966
1.208650255	0.274668183	0.804732197
1.058962345	0.317078602	0.843374349
0.849818661	0.215970977	0.881972877
1.389854258	0.242837403	0.813086457
1.002379806	0.21048019	0.781818724
1.067577512	0.224456138	0.810359695
1.207588639	0.230919381	0.880865566
1.007530544	0.200371707	0.744591372
1.00614907	0.256425718	0.69613304
0.690322324	0.295674552	1.060939004
0.666763582	0.244840914	0.959553824
1.044972218	0.16604112	0.613067703
0.838176908	0.241541417	0.768891747
1.088106146	0.258715233	1.150453509
0.961426339	0.28603299	0.806264014
0.918564496	0.305824574	0.78526194
1.288726463	0.320852079	0.623178019
1.372887754	0.270957279	0.795362501
0.978208725	0.32173561	0.800428816
1.074066066	0.224258802	0.677328314
1.291687296	0.160916713	0.672636592
0.919625194	0.257253565	0.738022501
0.733386633	0.24349669	0.764353408
1.335739289	0.206972186	0.097183358
0.761301443	0.236895876	0.683135988
1.242049917	0.307728431	0.689257614
0.814728854	0.273274965	0.780067677
1.229100339	0.199326776	0.813699887
1.067311291	0.250960825	0.815587822

1.102599716	0.261870297	0.731574724
1.046470049	0.208361261	0.842362169
0.657469596	0.294449381	0.640920849
1.154878151	0.281382126	0.940508373
1.050402383	0.320390381	1.065576426
0.993290261	0.245606994	0.677065235
1.030204175	0.232223695	0.760123982
1.143533937	0.306513062	0.7470043
1.145661016	0.208871014	0.680899792
1.109507319	0.290180599	0.980761322
0.77229506	0.265348957	0.76126491
1.330914854	0.26465296	0.697598234
1.200524651	0.244805812	0.740094329
1.075877983	0.245346969	0.91268161
1.064349357	0.222639404	1.189398907
1.058462481	0.223692647	0.826633999
0.903824074	0.30553037	0.89992036
1.067112254	0.228077903	0.681029561
0.901211027	0.287313575	0.660340914
0.992611321	0.226550636	0.868559441
1.142835193	0.241719342	0.764613583
1.27827902	0.17390225	0.669358692
0.739698526	0.266014409	0.820558099
0.884448274	0.213059604	0.936415647
1.07289606	0.237424453	0.83285295
0.660446134	0.248764647	0.849710495
1.12250547	0.218813879	0.647889055
1.199823495	0.311401922	0.667305543
0.942425819	0.239257541	0.745958227
0.71526404	0.309702907	0.869081412
1.054115835	0.307752761	0.859973553
0.890512292	0.220770332	0.780037935
0.935480801	0.260264426	1.070437961
1.57283192	0.235865739	0.556204329
1.420796596	0.18726055	0.792932604
1.266275189	0.232590893	0.648680657
0.653067167	0.3197604	0.743223098
0.96341687	0.197252498	0.720793623
0.84265216	0.276354908	1.33309172
0.889784849	0.182622517	0.654312012
0.833164706	0.134616056	0.828781665
1.086033563	0.376313066	0.87518847
0.837242104	0.202568523	0.732847028

1.042490099	0.272484391	0.659422469
0.860395097	0.208443553	0.587342554
0.906191094	0.232979163	0.725028018
0.517003219	0.273027139	0.807904059
0.92999134	0.265559551	0.786073685
1.048864043	0.190029773	0.676586031
0.988119867	0.204248868	0.86701316
1.007514781	0.313717684	0.711492281
0.848884795	0.2623085	0.931518076
1.105692009	0.233678275	0.576018836
0.7207469	0.271335488	0.709358341
0.937799605	0.196150111	0.688450319
0.886485548	0.278874861	0.91376043
0.877090696	0.250117446	0.711522381
1.027404707	0.171596838	0.484441306
1.009146231	0.252966006	1.021265536
1.032968355	0.205706301	0.58757764
0.659612118	0.281251403	0.909040593
1.253618424	0.279063622	0.914499536
1.100569966	0.261018336	0.673935504
1.140158814	0.168408972	0.902239893
0.915744919	0.244832952	0.61189919
1.282713459	0.349812146	0.693496107
0.844988947	0.215169105	0.770277387
1.453007807	0.194269922	0.819363691
1.030233035	0.195784501	0.952528062
1.033319076	0.300312584	0.73466756
1.1992773	0.284586756	0.763065346
1.370869855	0.271592462	0.521717289
0.859631772	0.228786736	0.597712115
1.213655018	0.244710705	0.655490953
0.905223905	0.247390997	0.883553473
1.217491216	0.206508123	0.564190427
1.01854902	0.225507009	0.752688526
1.067356149	0.175035825	0.807358362
1.003227485	0.382028101	0.553586897
1.220243414	0.32136707	0.81457718
0.94221248	0.256541155	0.738115863
1.179558241	0.203276254	0.845895468
0.914558389	0.324770186	1.086102907
0.801416291	0.262865745	0.877889669
1.035015428	0.021559603	0.699620886
1.082066112		0.95032281

0.811251855	0.865579777
0.91588688	0.710478151
0.908962873	0.649704115
1.106469678	0.665497007
1.0623013	0.665208474
0.720322484	0.765678208
1.102371226	0.944385142
0.938489301	1.134503572
0.954234744	0.970099663
0.718360421	0.913262513
0.884591853	0.76708806
0.845715923	0.733579039
1.04584826	1.058870001
1.367647725	0.888466653
1.260465301	0.644794826
0.970423538	0.557005859
0.940431194	0.635286134
1.271341496	0.724258977
1.047479283	0.709743605
0.691245498	0.640602336
1.00418843	0.814681613
0.938614505	0.545818359
1.181169974	1.108170276
0.81468298	0.680970771
0.871540217	0.166799037
0.812335588	0.728084643
1.002970642	0.828958114
1.148650626	0.844157948
0.874450623	0.729151674
0.999185644	0.699133854
0.813468719	0.941097725
1.181740588	0.560300171
0.99555755	0.853308758
1.009619097	1.3256134
0.957300714	0.643276836
1.073707017	0.770078657
1.261849032	1.024204614
1.290364147	0.611762109
0.841158881	0.942658418
1.028733268	0.584843224
1.140762264	0.855608205
0.857411928	0.791609104
1.201886392	0.654042887

1.165615472	0.901916123
1.241342259	0.779529256
0.915266781	0.679347864
0.849956619	0.665477204
1.114956437	0.95052602
1.216537342	0.567582545
0.928300904	0.723838532
0.976284566	1.187156385
0.714017935	0.79174469
0.934505861	0.847515427
1.489978597	0.575100553
1.226166926	0.705044466
0.919625194	0.905201792
0.985690222	0.808263937
0.928174167	0.74981108
0.719991255	0.697613998
1.501428888	0.535737798
0.884130888	0.989925113
0.872464928	0.638687676
0.848408428	0.736993796
1.286273147	0.982592107
1.13653484	0.786110295
0.852679299	0.85340669
0.957008332	0.600097351
0.911628453	0.746206219
0.827495166	0.601612626
0.88529252	0.580699314
0.61019156	0.582769312
0.593464792	0.702311735
0.982018644	0.693900816
1.037992793	0.901616417
0.857766997	0.762965931
1.22967026	0.696867458
0.576150724	0.961361146
0.981642307	0.650860326
0.48235243	0.656366108
0.878128279	0.680899792
0.931646439	1.008001141
1.12595136	0.721379022
1.292806432	0.750415279
0.723779088	0.719100064
0.775797557	0.762149955
1.108767965	1.015504806

1.012803077	0.915406322
0.957942911	0.549760416
0.685210929	0.677003204
0.848652478	0.727196797
0.907471557	0.884392556
0.867815324	0.802641983
1.159887632	0.547924242
0.898629185	0.682052743
0.763841691	0.571488961
1.107303805	0.587132611
0.927734764	0.851984728
1.051778741	0.771962486
0.925099154	0.593955068
0.921247108	0.958044813
1.1834839	0.911204457
1.189197068	0.717626944
0.825684986	0.697838412
1.084686126	0.7714788
0.52834835	0.63753541
0.909736751	0.924306824
0.87432865	0.746601665
0.865163465	0.911464133
1.335751892	0.695071408
0.832041842	0.631609212
1.170132415	0.700001499
1.213986459	0.402684174
0.82622576	0.865863783
0.727615538	0.816932345
1.504841227	0.211152548
1.030320819	0.978631107
1.194672912	0.715198189
1.327410066	0.567875826
0.658997202	1.311602049
1.033946038	0.702311735
1.217117723	0.779067116
0.715752942	0.62214712
	0.723529612
	0.755402255
	0.898959021
	0.600363258
	0.729472963
	0.760650729
	0.811992661
