# Supplementary material for: The O-GlcNAc transferase OGT is a conserved and essential regulator of the cellular and organismal response to hypertonic stress
Source: PLoS Genet. 2020 Oct 2;16(10):e1008821. doi: 10.1371/journal.pgen.1008821 (PMC7556452; doi:10.1371/journal.pgen.1008821)
Supplement: S15 Table — (PDF) [file pgen.1008821.s022.pdf]

L1	1.084619631	0.744151643	1.035923847	0.884991851	0.998366126	0.753466353
L4	0.724464529	0.979950034	0.867243085	0.712995869	0.810101675	0.844487996
Adult	0.706375533	1.494855896	0.922081871	1.36830476	0.754814735	0.6640898

0.776144848	1.122829872	0.632432339	1.160196428	0.719218583	0.78012391	0.91093344
0.734215287	1.834433416	0.688557485	0.778152916	1.114609835	0.564026521	0.868960491
0.808031823	1.06858086	0.887123733	0.676612335	0.699666039	1.260602935	0.82532668

0.846089736	0.590807281	1.052105283	0.59684458	0.764851294	0.697152591	0.746049808
0.961636323	1.063029347	0.831962359	0.812995584	0.929898567	0.923065014	1.137525743
1.631274661	0.840416674	1.048639518	2.039495727	0.747137837	0.786939393	0.160930852

1.114124977	0.736947771	0.663067209	0.683622562	0.874731105	0.627103767	1.263613675
0.713373588	0.755719966	0.866580517	1.286371483	1.330650958	0.54946001	0.785498335
0.86697292	0.764768655	0.905325451	1.030368518	1.099694158	1.091865602	1.306929327

1.309152721	1.107100778	0.774947732	1.011879536	0.810472745	1.384575026	0.835790891
1.042573332	0.801685854	1.224194371	0.966092155	1.70377945	0.961579773	1.319043856
1.044882494	1.021671432	0.907097558	1.300031567	1.154581981	0.925867522	0.866198121

1.205440208	0.772014615	0.863605608	0.942606162	1.074031057	0.788666303	0.872292588
1.357579525	0.819632283	1.108459257	0.9939672	0.680082821	0.99182107	0.821328659
1.125983964	1.07600156	1.022547362	1.116073343	1.033735832	1.370745111	1.250635377

0.986536686	1.250649497	0.987070981	0.999301178	0.868354172	0.923937169	1.59711321
0.665961308	0.777178	0.947750347	1.158749534	1.343536434	0.749138788	0.970357931
1.028069568	0.823511712	0.939536137	0.837658725	0.896983934	1.497839838	1.234127224

1.506391221	0.742338408	0.639146058	0.602585365	0.844627178	1.023790519	1.058764729
1.533927208	0.927818012	0.68233558	0.960629111	0.592845394	0.646106529	0.490844428
0.852429176	1.027162997	0.732305962	0.843391144	1.063393574	1.286976948	1.10592374

0.796778387	1.013806268	0.704940506	0.773699452	0.60638974	0.810326713	0.807057999
1.547026745	0.855963	0.674093498	0.968246927	1.272169905	0.760931828	1.113711255
0.897154343	1.048173934	0.964691054	1.217520228	0.995246879	1.339737264	0.708557201

0.609800039	0.888067555	0.761258577	0.745904521	1.048853131	0.674873126	0.813066719
0.779032782	1.08426203	0.756950208	0.738684244	0.932594424	0.808088166	1.035102625
0.81817979	1.042238567	0.734897617	0.674851141	1.020600441	0.758977967	1.300264321

0.68461898	1.141683619	0.776305287	0.611140259	0.985142616	0.834518322	1.668412906
0.817432912	1.127979524	0.733832604	0.788896072	0.559992292	0.815128809	1.164340409
0.644309278	0.995034809	0.683506676	0.760696901	0.919521573	0.864832246	1.111951586

1.783163793	0.664351951	1.26994177	1.024740232	0.793496626	0.748325647	1.980547135
1.239567341	0.911065887	1.158518707	0.872307533	1.027432493	0.710254384	0.898605576
1.007542986	0.956062333	0.861046561	1.158660714	1.088369394	0.783473091	0.993328687

0.644073928	1.040772826	1.091055839	0.754768508	0.605336458	1.328636921	0.93517952
1.03785542	0.518442107	0.807716392	0.917691101	0.707620342	0.608334148	0.935541089
0.751870967	0.933446	0.991082667	0.638460262	0.880007767	0.999181634	0.771028873

1.156639527	1.036674957	1.076974442	0.969032917	1.080114557	1.222280518	1.011995697
1.081502612	1.163116004	0.675127452	1.196855485	0.862153457	1.874510556	0.906714311
0.671512562	0.755562224	1.160522534	0.945003482	0.698810353	0.939536137	1.035131205

			ev RNAi			
1.218256549	1.12696733	0.747914672	0.710689535	0.987541778	0.927092477	0.60842408
0.687705169	0.857665671	1.507913238	1.146904485	0.910063357	0.86837822	0.608594483
1.470470372	1.033597558	1.276240298	0.88238874	0.792293794	1.443198288	1.113105062

0.484232648	1.012947417	0.603854371	0.893829533	0.929768941	0.639536675	1.007353515
0.940894539	0.909355343	0.828739308	1.097482261	0.919459292	0.724266816	1.422097038
0.689924579	0.966539627	0.810194505	0.742070042	1.030472809	0.781305929	1.082185477

0.784093532	1.260005225	0.770900965	1.571343181	0.657541649	0.743046649	0.237665656
0.755555679	0.977766466	0.641818643	1.009630203	0.946590877	0.660201918	1.053450847
0.905706	1.34744297	0.814000757	0.537296283	1.098414336	1.165655087	0.802925995

0.673069138	0.841732532	1.082801059	0.966959698	1.273364617	0.81829188	1.415431019
0.982897968	1.084162434	1.318469928	1.42903914	0.890143198	0.798477806	1.276098047
0.987627765	1.190888308	1.018807079	0.793034852	0.920166852	0.989426722	0.660587841

0.876233522	1.248909723	0.578238091	3.762481656	0.887285116	0.974495393	0.797329943
1.01727411	0.88268092	0.93680758	1.602632955	1.042573332	0.884714747	1.212377943
0.864421102	0.904290004	0.819650091	1.017050311	0.638897478	0.673369564	1.065018924

1.408623025	1.430654048	0.710643843	1.116763529	3.229234948	2.424189693	3.612517974
0.683539493	0.841368228	0.867739706	0.824935439	1.14980804	0.678560957	0.768786608
1.260555343	0.68591339	0.828066944	0.932705276	0.811169681	0.742070042	1.104374827

0.679944129	0.887285116	0.722937357	2.112583611	0.926551616		
1.215556352	0.815520415	2.00730149	1.068654573	1.088833372	1.381719559	1.629065521
0.838968444	0.931331942	4.077479597	0.775198169	0.541676934	1.040623803	0.765746985

0.724464529	0.921729561	1.126218531	1.049529143	0.783801363	0.78928865	0.729820813
1.490106476	0.862102088	0.974890121	0.585052323	0.906748599	1.188637192	1.021134219

0.47219689	0.905909365	0.906863959	0.932023332	0.562492743	0.914073338	0.829226641
0.833401739	1.082703074	0.819599748	1.019604237	1.682707791	1.03411051	0.89048405

1.10335115	2.42737253	3.071268499	0.760931828	1.059797815	0.768626644	1.525220707
0.716306241	1.011070432	1.038898058	0.758871628	1.604134388	0.755562224	0.751964309

0.959658907	1.082374892	0.706144736	1.207648025	0.840343027	0.871066697	0.878594434
1.184898831	0.727671668	1.61824005	1.080684527	1.143765441	0.760675879	3.646744205

1.076870722	0.865259586	0.533373542	0.750681833	0.773778729	0.988895636	0.667507813
0.781508729						

0.914823362	0.655232424	0.956918744	1.288122874	4.28024153	0.605378507	2.809867595

0.921701875	1.332024963	1.171133814	1.024870106	1.349680709	1.114965373
