# Supplementary material for: The O-GlcNAc transferase OGT is a conserved and essential regulator of the cellular and organismal response to hypertonic stress
Source: PLoS Genet. 2020 Oct 2;16(10):e1008821. doi: 10.1371/journal.pgen.1008821 (PMC7556452; doi:10.1371/journal.pgen.1008821)
Supplement: S16 Table — (PDF) [file pgen.1008821.s023.pdf]

gpdh-1 mRNA

			50mM NaCl			250m[illegible]
WT	0.896577376	1.51834538	0.656333056	1.119224928	12.66258458	19.73247478
ogt-1(dr20)	0.91806402	1.170128253	0.930879716		61.67725558	89.67703455

hmit-1.1 mRNA

			50mM NaCl			250m[illegible]
WT	1.464297133	1.324922831	0.501286864	1.028238545	101.7022885	163.7300138
ogt-1(dr20)	0.376218955	1.790601679	1.484432107		115.1771813	234.9149053

nlp-29 mRNA

			50mM NaCl			250m[illegible]
WT	0.868009858	1.114773027	1.264952607	0.816986041	2.483113144	3.021119673
ogt-1(dr20)	0.624997456	1.572059209	1.017777513		1.968534807	4.600388036

M	NaCl	
	16.13923174	22.66666132
	48.39099393	
M	NaCl	
	104.6243609	189.0610462
	159.3451293	
M	NaCl	
	4.659957078	3.059544042
	2.804004936	
