# Supplementary material for: The O-GlcNAc transferase OGT is a conserved and essential regulator of the cellular and organismal response to hypertonic stress
Source: PLoS Genet. 2020 Oct 2;16(10):e1008821. doi: 10.1371/journal.pgen.1008821 (PMC7556452; doi:10.1371/journal.pgen.1008821)
Supplement: S17 Table — (PDF) [file pgen.1008821.s024.pdf]

*gfp* mRNA

| | | | | | | |
|---|---|---|---|---|---|---|
| | | | 50mM NaCl | | | 250mM |
| WT | 1.115352704 | 1.901977263 | 0.701006633 | 0.67245051 | 12.18893355 | 16.88300726 |
| ogt-1(dr20) | 1.021012126 | 1.613283518 | 0.607097442 | | 32.89964245 | 50.91433496 |

| | | |
|---|---|---|
| M | NaCl | |
| | 12.27371429 | 10.39271004 |
| | 18.25221945 | |
