# Supplementary material for: The O-GlcNAc transferase OGT is a conserved and essential regulator of the cellular and organismal response to hypertonic stress
Source: PLoS Genet. 2020 Oct 2;16(10):e1008821. doi: 10.1371/journal.pgen.1008821 (PMC7556452; doi:10.1371/journal.pgen.1008821)
Supplement: S18 Table — (PDF) [file pgen.1008821.s025.pdf]

WT	ogt-1(dr34)
1.104378531	0.451751452
1.601967637	0.263619778
1.008693525	0.278435501
0.803084252	0.288655092
0.766395465	0.273882349
0.633208567	0.44796844
0.901681094	0.620722541
1.24830465	0.274207595
0.833225462	0.303904795
1.334407124	0.51565295
0.772873962	0.503633771
0.817869787	0.280869928
1.534771792	0.337908129
0.848867993	0.309749118
0.679688557	0.444073744
0.793871895	0.375999193
0.977515725	0.487452326
1.036596346	0.631901609
1.005676627	0.408904783
0.836846913	0.510093555
0.76525686	0.497811082
0.637510256	0.349358681
0.5455994	0.42530622
0.763418911	0.555184927
0.780932673	0.422385161
0.648944053	0.318431811
0.723323488	0.267854004
3.111421179	0.383093518
0.848194971	0.338155862
1.981546527	0.346715572
0.846053573	0.378968184
0.873884283	0.420518952
0.668837216	0.2433238
0.909729936	0.503613076
0.873018104	0.316788871
1.025505084	0.390471615
1.032507999	0.521401267
0.876576602	0.464373003
0.702385097	0.522176008
0.806817209	0.407950007
0.878116076	0.539295339
1.10056904	0.359530226

0.715784444	0.515549101
0.978446594	0.458949846
0.848775309	0.368798984
0.905740095	0.45963587
0.770085333	0.396940271
1.169388839	0.381550342
0.633181274	0.472790641
0.754012342	0.373820452
0.646373043	0.549100709
1.092542047	0.325454827
0.749335121	0.385627979
0.926658699	0.502993016
0.716004753	0.32694546
0.836293552	0.545742295
0.710276715	0.357000461
1.092868228	0.451899652
3.483861431	0.461142119
2.328471524	0.248036146
0.754044388	0.395903332
0.667039267	0.413116521
0.778337392	0.44310342
0.807379832	0.412264111
0.92032629	0.484759855
0.85283043	0.387807884
1.247722458	0.317807484
2.079537431	0.451679615
0.8857903	0.372572371
0.657943277	0.283864664
0.894193179	0.319864491
0.817716131	0.400478637
0.723590533	0.384549356
0.724621289	0.376219083
0.613561939	0.548202017
1.329313967	0.36065728
0.874393187	0.306558688
0.815312567	0.387706576
0.638983538	0.49590376
0.766520123	0.343753327
0.790288189	0.334707187
1.499542662	0.389625916
0.744892553	0.326198441
0.524404357	0.338048689
1.999192723	

0.872549052
0.776927238
1.057816317
1.01745208
1.132806685
0.805488696
0.818700887
0.704323558
1.055011085
2.086013707
0.844132805
0.77125803
1.609317937
0.674091664
0.756371369
1.084457617
1.497176562
2.227356934
0.970923723
1.528523959
0.685158841
0.526242056
0.858223754
0.865744639
0.697578414
0.722491929
1.004281442
0.792197162
0.696595483
2.497702723
0.824610795
1.022861434
0.538330312
0.849963532
1.425206359
0.510573266
0.721184738
1.046675195
0.727566904
1.005102202
0.941082953
1.814553639
1.155925189

0.713929119
1.69799358
0.756047319
1.516834746
0.659592257
0.913064139
0.816334189
