# Supplementary material for: The O-GlcNAc transferase OGT is a conserved and essential regulator of the cellular and organismal response to hypertonic stress
Source: PLoS Genet. 2020 Oct 2;16(10):e1008821. doi: 10.1371/journal.pgen.1008821 (PMC7556452; doi:10.1371/journal.pgen.1008821)
Supplement: S19 Table — (PDF) [file pgen.1008821.s026.pdf]

gpdh-1 mRNA

	50mM NaCl				250mM
WT	0.92873141	0.872564288	1.23399225	78.97550398	77.88822487
ogt-1(dr34)	1.101905116	0.952637998	0.952637998	73.00887782	100.4267645

M NaCl
81.1958076
96.33579183
