# Supplementary material for: The O-GlcNAc transferase OGT is a conserved and essential regulator of the cellular and organismal response to hypertonic stress
Source: PLoS Genet. 2020 Oct 2;16(10):e1008821. doi: 10.1371/journal.pgen.1008821 (PMC7556452; doi:10.1371/journal.pgen.1008821)
Supplement: S20 Table — (PDF) [file pgen.1008821.s027.pdf]

| *gpdh-1(dr81)* | | *gpdh-1(dr81);ogt-1(dr83)* | |
|---|---|---|---|
| 50mM | 250mM | 50mM | 250mM |
| 0.007189001 | 1.34254366 | 0.005042003 | 0.667210122 |
| 0.006721055 | 0.82259621 | 0.005901857 | 0.431842437 |
| 0.002096393 | 0.83486013 | 0.003520223 | 0.421250871 |
