# Supplementary material for: The O-GlcNAc transferase OGT is a conserved and essential regulator of the cellular and organismal response to hypertonic stress
Source: PLoS Genet. 2020 Oct 2;16(10):e1008821. doi: 10.1371/journal.pgen.1008821 (PMC7556452; doi:10.1371/journal.pgen.1008821)
Supplement: S21 Table — (PDF) [file pgen.1008821.s028.pdf]

	Unadapted				
WT(drIs4)	0	0	0	0	0
ogt-1(dr15)	0	0	0	0	0
ogt-1(dr20)	0	0	0	0	0
ogt-1(ok430)	0	0	0	0	0
ogt-1(ok1474)	0	0	0	0	0
WT(N2)	0	0	0	0	0
gpdh-1(ok1558)	0	0	0	0	0

		Adapted		
73.68421053	55	60	80	10
5	10	0	10	0
5	5.263157895	0	0	0
0	0	0	0	0
5	0	0	0	0
60	70	60	55	40
10	45	10	25	20
