# Supplementary material for: The O-GlcNAc transferase OGT is a conserved and essential regulator of the cellular and organismal response to hypertonic stress
Source: PLoS Genet. 2020 Oct 2;16(10):e1008821. doi: 10.1371/journal.pgen.1008821 (PMC7556452; doi:10.1371/journal.pgen.1008821)
Supplement: S22 Table — (PDF) [file pgen.1008821.s029.pdf]

osm-8(dr9)	·8(dr9);ogt-1(dr20)
1.781579314	0.532484438
0.835788784	0.29353005
1.099582967	0.235527407
1.006879664	0.263205235
1.454063909	0.355160375
0.955741299	0.216351198
0.502434962	0.332733222
1.323143148	0.322717841
1.994839204	0.331210416
0.862591613	0.372918394
0.69429789	0.335626555
0.888423233	0.295445911
0.8989997	0.440910681
0.535574289	0.209766597
0.804105287	0.278261812
0.614316462	0.29514898
0.517942214	0.263525875
0.686846379	1.194504231
0.915647843	0.375508105
1.136235732	0.34501085
0.937593122	0.370176347
0.788865834	0.234376086
1.198666267	0.328550091
0.971550553	0.215349738
0.677707466	0.239207523
1.273582909	0.349610995
0.747444195	0.553230585
0.977321644	0.415753615
0.985903004	0.223155954
1.165369982	0.786624738
0.715482965	0.288669628
1.242039059	0.215809256
1.064487207	0.291849178
0.885681186	0.367091544
0.65652141	0.770029279
1.92985269	0.398097191
0.738950511	0.5940293
2.200829867	0.384305215
1.369904305	0.450505443
1.398443978	0.466147993
0.815758987	0.425017287
1.242039059	0.192642793

0.973144005	0.200158966
0.76536461	0.414922939
1.221425753	0.675914589
1.040019261	0.585834639
2.20125441	0.444959448
1.02386077	0.346721647
1.25859958	0.572090718
0.942429637	0.235452302
0.845833051	0.508525083
0.922973025	0.240357559
0.561003689	0.252496829
0.882195967	0.408636227
0.705249765	0.37947726
0.60599239	0.279688796
1.214438191	0.161358921
0.760918047	0.394192262
0.603873536	0.559377591
0.729622945	0.466147993
0.722725249	0.232082192
0.86374481	0.464483178
0.997251033	0.240592512
0.786624738	0.265367381
0.859044158	0.364811473
1.275051862	0.30590962
0.68878584	0.514521841
0.83518182	0.670087739
1.885244097	0.404549865
0.557889885	0.82724855
1.314537339	0.362097101
0.788484371	0.520588634
0.471974843	0.351631867
0.750743609	0.308131724
0.846426618	0.922973025
0.939754353	0.38290728
0.744333085	0.30590962
1.482046607	0.200925859
0.951267125	0.354151397
1.514980976	0.264821617
0.974971506	0.450738142
0.770029279	0.572090718
0.786624738	0.360536338
0.902484195	0.243022277
0.865703415	0.556523624

0.864686734	0.355744521
1.56436106	0.30400956
0.599333133	0.386412152
3.43254431	0.529713628
1.685010367	0.8989997
0.624836671	0.471385611
0.44949985	0.361666546
1.176739445	0.494954891
1.34344899	0.299666567
1.000425307	0.401192945
1.610707796	0.437974213
0.772824304	0.416203565
1.830690298	0.402582357
0.578666474	0.43077069
0.632173305	0.328330325
1.002218184	0.343583219
1.357313273	0.204650338
1.066916311	0.462720434
0.672836254	0.44781633
1.012666329	0.524416492
0.545967854	1.207747072
0.699221989	0.248875623
0.889918895	0.287914937
1.265832911	0.360205267
0.620737888	
0.908988586	
0.495811956	
0.762787624	
0.839066387	
1.304645906	
1.445688707	
1.225602587	
0.649277561	
0.610230099	
0.867304198	
1.198666267	
1.007702278	
0.754556103	
1.20537522	
0.553550741	
1.198666267	
1.28656846	
0.653371367	

1.191855663
0.889918895
0.874027486
1.509296245
1.000050054
0.524416492
0.746285007
0.669467862
0.829309801
0.61531535
1.075726137
0.684952152
0.801547158
1.426412858
0.653817964
0.943949685
0.84362653
1.210191904
1.915260231
