# Supplementary material for: The O-GlcNAc transferase OGT is a conserved and essential regulator of the cellular and organismal response to hypertonic stress
Source: PLoS Genet. 2020 Oct 2;16(10):e1008821. doi: 10.1371/journal.pgen.1008821 (PMC7556452; doi:10.1371/journal.pgen.1008821)
Supplement: S23 Table — (PDF) [file pgen.1008821.s030.pdf]

			osm-8(dr9)			
500mM NaCl	80	100	100	100	100	20
700mM NaCl	30	100	100	100	90	0

ogt-1(dr20);osm-8(dr9)

40	30	20	30
0	20	10	10
