# Supplementary material for: The O-GlcNAc transferase OGT is a conserved and essential regulator of the cellular and organismal response to hypertonic stress
Source: PLoS Genet. 2020 Oct 2;16(10):e1008821. doi: 10.1371/journal.pgen.1008821 (PMC7556452; doi:10.1371/journal.pgen.1008821)
Supplement: S24 Table — (PDF) [file pgen.1008821.s031.pdf]

WT	*ogt-1(dr20)*	*ogt-1p*	*dpy-7p*	*nhx-2p*	*myo-3p*	*rab-3p*
1.35735729	-0.0406955	0.9090692	0.87080048	0.35701294	0.13292042	0.0371497
0.6031361	-0.0614692	0.8199028	1.1995701	0.41719123	0.05301068	-0.0436303
1.47184937	-0.0396087	1.33743868	1.15276879	0.30290725	0.09419211	0.09294081
1.13397262	-0.151664	0.96068288	0.77446036	0.22222978	0.16447455	0.24442125
0.9338797	-0.1133232	0.85749219	0.41137734	0.2016192	0.17326728	0.03482622
0.69307164	0.01794558	1.08825022	1.19004483	-0.0829995	0.16264072	0.05753739
1.48705646	0.11579361	0.78076284	1.11547907	0.36620061	0.13939278	0.01408001
0.85769565	-0.0080054	0.84938914	0.70144382	0.19164704	0.30981295	0.09282946
1.10296627	-0.0494189	0.6849885	0.38634908	-0.1851008	0.194387	0.06024826
1.27901823	-0.0072574	0.89991503	0.6742284	0.60318278	0.39466381	-0.0322714
0.96120671	0.04393094	0.81400602	0.5017126	0.4357072	0.37688271	0.16072057
1.26702552	0.0314073	1.21909095	0.91359389	0.24756613	0.09855162	-0.0300609
1.18631256	0.06730583	0.8507362	0.97511727	0.48356627	0.02081834	0.05728876
1.34850207	0.11393066	0.38184948	0.50229944	0.32589376	0.0500063	0.05820316
1.39516061	0.10924809	0.6941588	0.6243594	0.30400095	0.20807118	-0.1294196
1.23743387	0.02906211	0.49076181	1.19335618	0.30869082	0.3434942	0.00817296
0.83474276	-0.0345217	0.74182953	0.84411024	0.24921334	0.02472402	0.09651962
1.40000319	-0.0465453	0.68671285	0.98049443	0.40153816	0.29574238	-0.00008802
0.73936494	0.01658396	0.77000358	0.68610559	0.19200968	0.2826083	0.00952659
1.19431768	-0.0065536	0.59745164	1.3453999	0.08169258	0.45198347	0.07906985
1.19967356	0.02746175	1.19089986	0.76666088	0.30101322	0.02000816	0.03669303
1.75512526	-0.0015907	0.69687779	1.64452949	0.15840633	0.10792138	-0.0366145
1.59826385	0.01806105	1.30258637	0.43071087	0.39666081	0.30240261	0.04687802
1.3752574	0.06069643	0.68075626	1.31685723	0.40004212	0.71576712	0.01643897
0.8470125	-0.0322753	1.27569069	0.69345105	0.14232283	-0.0169418	0.14731046
1.31019545	0.04786423	0.49153882	0.77376169	0.27831677	0.14170517	-0.0650374
0.89478031	-0.1357778	0.9870805	1.46597465	0.40981623	0.22744526	0.14379081
0.88790101	-0.054691	1.12737875	0.85241654	0.19430044	0.00586909	0.06219903
0.91362816	-0.0447254	0.89319059	0.65972332	0.16464716	0.20729459	-0.0330389
0.47843291	0.24604927	0.67932358	0.90023152	0.11915429	0.17588877	0.0359956
1.31138409	0.08944951	1.05397857	1.09358813	-0.0805801	0.16022128	0.04296152
0.67818298	0.08453765	0.85717139	1.29342718	0.1885661	0.03553331	0.02236061
0.38958206	0.04024536	0.59970774	0.40792921	0.10334382	0.22070726	-0.0212973
0.80787703	-0.0125801	0.73366336	0.62219493	0.15933797	0.04649041	-0.0247985
1.01044503	-0.0434982	0.688016	1.56192847	0.34221202	0.03015128	-0.0995832
0.87162841	0.02504586	0.94520427	0.85341827	0.37323464	0.49132487	-0.0172752
0.80419158	-0.0361074	1.03237325	0.2546346	0.37034999	0.19928457	0.05332856
1.10063573	0.08453765	0.87297079	1.06381422	0.47291349	0.28018849	0.01202809
0.62846943	0.09552857	0.68247858	1.03532925	0.46208166	0.23038842	0.13831866
0.56666837	0.19946692	0.91749664	0.95990469	0.51504118	-0.0867104	0.14903336
0.67483166	-0.0748575	0.43495346	0.43195485	0.38618757	0.04063078	0.00100373
1.06296005	0.03663649	0.80152245	0.8834704	0.49069618	-0.0751719	0.43666509

1.2598534	0.15932188	1.27840287	1.29214399	0.27731973	0.23266153	0.00375013
1.29278451	-0.0761505	0.83293045	0.64287431	0.41202672	0.05466033	0.02628541
0.84208881	0.06341587	0.87197357	0.94486227	0.28844804	0.17462271	0.00458441
1.06132568	-0.0767599	0.82933218	0.6765236	-0.1952601	0.30958462	0.0874904
1.14149662	-0.1010279	1.00109473	0.69221537	0.55892457	0.23334914	0.20081002
0.82067324	-0.0935797	1.0658166	-0.0211619	-0.227206	0.31698361	0.13359969
1.46439633	0.02447109	0.54993359	1.407132	0.35280944	0.05309836	0.16351935
1.86678519	-0.0361074	0.65299652	0.48586792	0.36628571	0.13740057	0.10971425
1.10860351	0.02069561	0.62908133	1.69203404	0.10681906	0.32766471	0.06127765
0.70307893	-0.1046594	0.91371332	1.29268568	0.43779038	-0.1204309	-0.1442765
0.80708694	-0.0385589	0.61736237	1.03747283	0.15770454	-0.2352277	0.0330127
0.61094038	0.00473729	1.02909972	0.93772285	0.13507273	0.39898173	0.10847959
0.93327549	-0.0200843	0.75592017	1.22569267	0.19035941	0.14379149	-0.0251342
0.98771449	-0.0343939	0.84461275	0.92201943	0.18461961	-0.0064238	-0.0477689
0.81160285	-0.1083219	0.74907192	1.02745924	0.45299202	0.05739889	0.16398433
1.13617977	0.0097312	0.57750003	0.43833387	0.18042816	0.37437771	0.17566889
0.85943681	0.05937322	0.97818552	1.08736791	0.58698587	0.42112763	0.14681373
0.80043558	-0.068326	1.00305986	1.25950438	0.09578378	-0.0546594	0.10678671
0.85541461	0.17821766	0.82097856	0.80577959	0.4238517	0.09508048	0.13243196
1.16076746	-0.1274391	0.70343073	0.81932645	0.22744209	0.23438477	0.14731046
1.23864675	0.05252217	0.60452051	0.49117338	0.56230692	0.07257833	-0.0010346
0.86614337	-0.0168575	0.44227815	0.43101476	0.43423514	0.08730754	-0.0136304
0.83328877	0.10220214	0.84473496	0.60536323	0.33606618	0.03135985	-0.0105225
2.06467294	0.15348948	0.71556385	1.54799568	0.35081619	0.49451132	0.33884978
0.81825104	-0.0906002	0.91231068	1.03582825	0.08999633	-0.0074856	0.12006138
1.21295192	0.1887091	0.84948964	0.69761881	0.14093151	-0.0064238	0.00528606
1.31472357	-0.0244578	0.61963529	0.66151298	0.15401653	0.18854285	0.01786725
0.62998334	-0.0182509	0.76079716	1.26968451	0.47951128	0.15014775	0.07747966
1.26968351	0.03174524	1.27340718	0.86201713	0.2939233	0.10447823	-0.0559684
1.21985944	-0.037636	0.44152625	0.63456416	0.3490385	0.06965233	0.04283044
0.40182001	0.04283631	1.21850404	1.44600236	0.1252907	0.20120995	0.00142649
1.36833138	0.02808904	0.5202262	0.57157808	0.4446552	0.07322413	0.12971871
1.5177815	0.00156005	0.62772091	0.58464434	0.25803388	0.52614628	0.39778419
0.72714031	0.00241385	0.48978297	0.63950116	-0.0673899	-0.0002837	0.18444356
1.18866719	0.00216954	-0.1012431	0.75536772	0.58038684	0.06947345	0.00721302
0.84163338	-0.1251787	0.78771562	1.30682048	0.32633663	0.24917126	-0.0161069
0.6960041	-0.057184	1.01743776	0.65487786	0.3020277	0.11006726	0.07563768
1.21892552	0.04319935	0.52310154	0.99358663	0.58365561	0.24107243	0.05646961
0.95429834	0.03763523	0.90202712	1.17602299	-0.0769341	0.03674678	0.12602151
-0.1589849	-0.057698	0.95478627	1.34217263	0.29201821	0.0892955	-0.0630268
0.24225555	-0.1079684	0.76249955	0.7390885	0.44910756	-0.1574572	0.02488535
1.3120067	0.07417758	0.56503467	0.9956237	0.2559134	0.39759837	0.08813738
1.17011085	0.08322454	0.95959406	-0.1385732	0.27247134	0.14251461	0.01171628

0.89572788	0.0981891	0.96805706	0.68832005	0.03732657	0.17462271	0.05011531
0.66681691	0.21867223	0.93616903	0.63720334	0.45835258	0.21200919	0.18458836
1.14576806	-0.0356683	0.85087452	0.97376453	0.26932033	0.2898341	0.06875683
1.71460349	-0.1759862	1.22718406	0.96498783	-0.0043186	0.17422949	0.0322675
0.99023415	-0.0063453	0.4484337	1.10940621	0.45106625	0.33520693	0.06580114
1.13767405	0.04445184	0.6186903	0.82476245	0.56516206	0.36664169	0.1632161
1.22208067	0.0390142	1.21532198	1.16502859	0.25658003	0.43016677	0.01838905
1.25698417	-0.1574408	0.63576449	1.00540295	0.23226983	0.10130637	-0.1640288
0.96376232	-0.0290819	0.69819156	0.52283884	0.5951581	0.56086002	-0.0388596
0.77653313	-0.0320228	1.13290458	0.59290036	0.30704326	0.35727491	-0.033766
1.25956855	-0.0331801	1.55563059	0.78691687	0.48816129	0.23665961	-0.0436303
1.39040226	-0.1159584	0.78388891	0.34738974	0.6239381	0.42701187	0.08045659
0.72471462	0.29722529	0.89517922	0.82476245	0.18325078	0.08782807	0.03056002
1.50822804	-0.0282037	-0.0263023	1.09918432	0.41695333	-0.1439578	0.02650403
0.89479994	-0.1342474	0.56748281	1.0597975	0.26884483	0.04817118	-0.0626048
1.41018757	-0.1833378	1.17202586	-0.1467876	-0.043934	0.10723491	0.19695494
1.01086376	-0.1996324	0.04638508	0.23964991	0.4929479	-0.0528956	0.23543362
1.53925002	-0.0243982	0.5995452	0.86091676	-0.2244341	-0.0216165	0.0335431
1.02795461	-0.1139285	1.21351511	0.98765186	0.20101336	-0.2178609	0.08058143
0.67519754	0.03714223	0.56321739	-0.2564151	0.07853388	0.32087064	-0.2558869
2.2694736	0.0042893	0.88136768	0.81631071	0.38419779	0.02287369	0.19268878
0.80120841	0.15243987	0.75023412	1.22963591	0.67641091	0.28143813	0.12218128
0.79579001	-0.088312	0.57349621	1.002019	0.72917727	0.25345155	0.11112237
0.76968081	-0.011825	0.97726878	1.13333657	-0.1185366	0.21569807	0.24198513
-0.2754106	-0.0701745	0.27885994	0.88444113	0.40981623	-0.2387829	0.37287098
0.69662483		-0.2207613	-0.1250735	0.28837154	0.34558155	-0.0211595
1.02581253		-0.0753956	0.16392448	0.12195315	-0.0816639	0.25337036
1.2628615			-0.1250735	0.08345109	-0.1229376	0.05728876
0.88011038			0.90721908	0.6509988		0.11343846
1.1339165				0.2000783		0.06781683
0.71834562				0.19995606		0.2901789
1.39260725				0.44508799		0.05291997
1.74400266				0.47604076		0.3982817
1.48488425						-0.0294057
1.2165907						
-0.2252067						
-0.2857712						
-0.1970442						
0.88936924						
1.38626789						
1.44696952						
-0.2056085						
0.81279879						

1.19143092
-0.0643911
