# Supplementary material for: The O-GlcNAc transferase OGT is a conserved and essential regulator of the cellular and organismal response to hypertonic stress
Source: PLoS Genet. 2020 Oct 2;16(10):e1008821. doi: 10.1371/journal.pgen.1008821 (PMC7556452; doi:10.1371/journal.pgen.1008821)
Supplement: S25 Table — (PDF) [file pgen.1008821.s032.pdf]

WT	*ogt-1(dr20)*	*Ce* OGT	*hs* OGT	*hs* OGT H498A
0.63059554	-0.0170701	0.89971849	-0.1321337	0.31752395
0.85563988	-0.1080803	1.5356763	0.11962123	0.22516553
1.03292592	0.02772431	0.62239014	0.07127747	0.07102873
1.17713058	-0.0525086	1.47239997	0.04770674	0.06882475
0.9099938	-0.0491342	0.44103953	0.50980851	0.23065091
1.26974688	-0.0476583	0.31152187	0.16029209	0.36955988
1.26523219	0.05779264	0.58912502	0.08414383	0.214325
0.61169903	-0.0686067	0.64718424	0.32100345	0.18083865
1.11061804	-0.0485407	0.70716895	0.1197929	0.14353505
0.79223805	0.03764593	0.4516541	0.24931587	0.02744808
0.89462493	0.00825568	0.41855961	0.21534072	0.23765457
1.23790442	-0.0545633	0.62512342	0.1614939	0.00537919
1.14619568	0.05805828	0.59464978	0.19749436	0.10022731
0.97470959	0.01876278	0.91021193	-0.0401393	0.14818774
1.83118999	-0.0236965	0.75395758	0.18564822	0.11376412
0.45854911	-0.0644158	0.50558189	0.07580215	0.25949438
0.89262525	0.11208064	0.66554962	0.19565439	0.2153438
0.90234201	0.04570959	0.51992497	0.09584981	0.24121062
1.31590948	0.05838291	0.23648217	0.22797057	0.1290594
0.89464715	0.01464819	0.64853764	0.03079249	0.12245884
0.71908536	0.02649575	0.61592819	-0.1810946	0.10953286
0.93340853	-0.0125528	0.9750577	0.09166699	0.13176436
1.17977931	0.01030992	0.39758269	0.15676617	0.11944809
1.04038907	-0.072438	0.34540365	0.12451026	-0.0019637
0.78723821	-0.0111154	0.78222821	0.07463148	-0.0029562
2.87589355	-0.0189595	0.24925657	0.19155214	0.1024844
1.35322408	-0.0368637	0.56862077	0.16833632	0.16492854
0.67730674	-0.0489306	1.17991618	0.21601311	0.21771209
1.05724108	0.18502067	0.58366567	0.25617096	0.21746145
1.06104105	0.04102403	1.47977206	0.10605488	0.22758765
0.55873807	0.01499104	0.6940983	0.21314697	0.1185924
0.89171068	-0.0478451	0.35376326	0.19770017	0.2750029
1.01876205	0.06607742	0.34932474	0.12586932	0.30278937
0.98057341		0.63407293	0.49676255	0.20232843
0.95487512		0.44082177	0.13432829	0.32149125
-0.2937839		0.36989813	0.18174045	0.2038509
