# Supplementary material for: The O-GlcNAc transferase OGT is a conserved and essential regulator of the cellular and organismal response to hypertonic stress
Source: PLoS Genet. 2020 Oct 2;16(10):e1008821. doi: 10.1371/journal.pgen.1008821 (PMC7556452; doi:10.1371/journal.pgen.1008821)
Supplement: S26 Table — (PDF) [file pgen.1008821.s033.pdf]

rIs4 50mM NaC	Is4 250mM Na	Ir20 50mM NaC	r20 250mM Na	Ir86 50mM NaC	r86 250mM Na	Ir90 50mM NaC
0.996205094	7.437863669	1.418048806	2.222044516	0.762741066	5.402335326	0.805026255
1.000996967	6.853976377	1.160922744	2.124895169	1.242866495	3.962797583	1.125701721
1.109721588	7.966374508	1.344804124	2.633868177	1.166513784	4.774759072	1.005703496
0.990285023	5.130023103	1.208346058	2.485380367	0.80715228	3.255976481	1.093795765
1.136189086	4.928747553	1.256515981	1.108265974	1.006579228	3.80289366	1.036416315
0.886533188	5.30858094	0.939213524	1.779126048	1.181126974	5.049806379	1.07603841
1.173101326	5.765403658	1.128389751	1.25821798	1.073871433	3.721868844	1.227335109
0.652523753	8.764464546	0.966072296	1.282705816	0.908793678	4.422036373	0.81892999
1.046015349	5.357294828	0.922521741	1.498211409	1.056425488	7.099416776	0.798831839
1.110656747	4.65451806	1.009190221	1.940403602	1.178959769	5.251472238	1.433431871
1.409846775	6.713958984	0.748515857	1.995677392	0.861204003	6.500092182	0.633429102
0.979505703	7.525876667	0.793847513	2.382931077	1.004180486	3.69676563	1.031371712
1.112026026	7.210829215	1.196152657	1.900269222	1.105169167	5.221769341	1.105335781
1.134233511	6.983690713	0.875157145	1.003717475	0.879269809	2.684919836	1.113788068
1.070724373	6.866783115	0.767584593	1.90092613	0.945061326	4.336077995	1.357749262
1.129696577	7.534806943	1.229076417	2.31430445	1.086846118	5.748530657	1.306447522
0.802887457	6.88155039	1.052087948	2.236024063	0.87466689	7.556417067	1.247127329
0.698637339	7.574593907	1.058329571	1.47633971	0.903102708	3.826499695	1.235306217
1.018384755	6.485794455	1.063083971	3.150302319	0.838670041	3.859701428	0.986953092
0.781201337	4.984368442	1.196657256	2.138504328	1.134128982	4.84065361	0.759447981
1.009441386	6.342859031	0.975600017	1.483229295	0.956624924	4.24117857	0.905090514
1.118993164	6.643217258	1.143996268	1.929806255	0.759004053	4.41102959	1.031312387
1.004174735	5.489614388	0.936005317	1.893572753	0.823760351	3.403839941	0.789990943
1.021689411	6.713035215	1.046164863	1.320225458	0.956624924	4.703405876	0.907484142
0.824133305	4.912842582	0.798388975	2.2149995	0.791111313	5.516873901	1.031764915
1.070022259	4.879837898	0.904840838	1.430364366	1.400193139	3.932791354	0.771152698
0.682506469	6.339168397	0.809753942	2.438736823	1.00910643	4.533035532	0.910796127
1.035341723	5.471913163	1.257949457	2.608311491	0.945265003	1.390095592	0.930030331
0.904495625	7.135830737	1.052569954	1.594380385	0.915289279	4.085933512	0.893885147
0.945712507	5.475285091	0.929295996	2.712643951	1.363465409	4.116581377	0.729702161
1.126922614	6.102239128	0.978670356	0.909235639	1.032888366	4.682427259	1.027873561
0.784511512	7.258436944	1.175921745	0.974739953	0.802881633	4.334445826	1.063565889
0.961659272	3.258467679	1.043915046	2.308385513	1.121229367	2.64409791	0.99440791
1.142658071	5.683350063	0.883653816	1.935047537	0.939220023	5.093480034	0.814776441
0.847079915	7.77881837	0.936776397	1.789599771	0.66316487	3.869031853	
1.113239986	5.617489837	1.148628747	1.351727868	0.804931543	4.062130282	
0.972352296	5.187752425	1.054023379	0.922351649	0.794340339	3.906591829	
0.931170934	4.509766774	0.927259783	1.595946294	1.042201594	3.547293378	
0.919948353	5.809275327	0.783395754	2.392707315	1.010978613	4.957630893	
0.999731484	6.898047167	1.000179594	1.822678705	1.030322614	2.757365313	
0.994039431	9.209482964	1.00554672	2.564522161	1.040539391	3.111028132	
0.841914793	4.86573432	1.008890581	1.257131811	0.845101293	3.276276932	

1.148565873	6.406572179	0.91497911	1.49354616	0.840745101	4.521918523
0.78691063	5.306529694	0.939057508	1.072583168	1.080790435	5.01384906
1.169804148	5.68941392	0.942302683	1.560120971	1.153264492	4.186640843
0.863187451	6.802757165	1.267486852	2.347197874	1.045839944	3.578631033
1.042395919	5.626106326	0.942198169	1.439339859	1.258438322	3.786545194
0.995906829	7.406227559	0.730175235	1.551718959	0.994034837	4.297880093
0.945194593	7.500274893	0.860985128	1.8086581	1.445071124	3.825733169
1.12903244	7.410752812	0.910488605	1.582294702	1.116241554	3.355316552
1.093997704	4.613335228	0.844503299	1.818054294		4.757636313
1.011571169	4.885174387	0.713488715	1.528052158		4.322647537
1.182098899	3.848547039	0.798388975	1.606864496		3.836232798
1.125459553	5.423031033		1.280970755		3.366583867
1.014763572	6.868994804		2.057457564		3.831619248
	7.612178927		1.653535459		3.644695079
	7.143519457		1.172513277		3.826499695
	7.215371183		1.10052441		2.958232985
	6.146497272		1.160822361		4.567577961
	6.48935736		1.900132424		3.266830433
	5.413501238		1.625291841		3.70692158
	5.954674979		1.382342541		4.131673011
	7.043340516		2.022585402		2.762020001
	6.862978569		1.448040357		3.823604342
	8.158919722		0.90143082		4.135912505
	6.567646495		1.34704992		3.816195912
	6.748948348		0.797190192		3.377904872
	6.256899152		1.984780067		2.430768249
	8.480725011		2.01673978		4.40682985
	7.108039186		1.756906811		3.373835873
	4.68184362		1.358452882		4.241407947
	2.784756095		3.47523265		4.499596635
	5.858756047		1.116950147		3.338277445
	7.764728739		1.165083757		4.593853737
	8.759983527				3.27547465
	5.824213463				5.56038237
	7.759532044				3.265302764
	5.957253191				4.133253691
	6.36230215				4.335213373
	8.706972835				2.695942967
	6.71651961				5.941977238
	5.245925463				3.778800946
	8.513655363				2.941621641
	6.838325344				7.917045184
	9.090371486				

7.714797591
5.780611139
9.885658014
6.728423056
5.096375535
6.725454297
5.052786008
5.0274455
6.25524129
5.105515376

r90 250mM NaCl
5.106470169
4.838255642
4.75348836
5.31637066
3.619594866
4.783473845
3.651768402
6.759449778
4.420514538
5.675714618
6.541125009
6.906962633
5.659578517
4.472855533
5.373261364
4.067745346
4.026356579
3.171842053
3.449501123
4.087056369
6.599750652
4.00635051
4.680318954
4.583388042
6.016360841
5.509841686
4.364514584
6.250194831
5.065952608
6.18056154
4.522891663
5.564408934
4.280919342
4.151808077
3.875887609
5.770012526
7.744046353
4.590115173
4.008669785
4.845065421
4.808761481
3.817195198

3.640210873
3.140356768
4.797232118
7.449722221
2.834831746
4.676498737
3.468403714
5.565542622
6.092915747
5.651035213
4.495146602
4.244305675
6.006459138
6.797999483
5.508764844
5.488088433
4.603323695
3.715924911
6.305426341
4.833895626
5.414390031
4.07666717
10.12419323
3.639389526
