# Supplementary material for: The O-GlcNAc transferase OGT is a conserved and essential regulator of the cellular and organismal response to hypertonic stress
Source: PLoS Genet. 2020 Oct 2;16(10):e1008821. doi: 10.1371/journal.pgen.1008821 (PMC7556452; doi:10.1371/journal.pgen.1008821)
Supplement: S27 Table — (PDF) [file pgen.1008821.s034.pdf]

	Unadapted							Adapted				
WT(drIs4)	0	0	0	0	0			20.0	25.0	65.0	50.0	70.0
ogt-1(dr20)	0	0	0	0	0			5.0	5.0	25.0	0.0	10.0
ogt-1(dr86)	0	0	0	0	0			40.0	65.0	40.0	35.0	55.0
ogt-1(dr90)	0	0	0	0	0			78.9	80.0	86.7	73.7	71.4
