# Supplementary material for: The O-GlcNAc transferase OGT is a conserved and essential regulator of the cellular and organismal response to hypertonic stress
Source: PLoS Genet. 2020 Oct 2;16(10):e1008821. doi: 10.1371/journal.pgen.1008821 (PMC7556452; doi:10.1371/journal.pgen.1008821)
Supplement: S28 Table — (PDF) [file pgen.1008821.s035.pdf]

ev(RNAi)	*ogt-1(RNAi)*
0.846364794	0.349242811
0.771397344	0.376181128
0.714720603	0.37585543
1.101702575	0.613454595
1.146974969	0.441437982
0.992552413	0.362225243
0.64283112	0.402686005
0.607854422	0.522065745
1.336465379	0.367624395
0.859297853	0.506362273
1.711609544	0.354408755
0.599528303	0.436587015
0.801730068	0.344690466
1.092040102	0.508912567
0.63984676	0.350161744
0.80428917	0.319795694
1.064109541	0.460329339
1.154053334	0.332335328
0.900470733	0.380800896
0.79713487	0.2991149
1.370667618	0.631743422
0.829329508	0.543025493
0.677171155	0.400762234
0.693075392	0.661261168
0.694786689	0.601519887
0.69857599	0.296096635
0.965378788	0.35790464
0.861869872	0.462625882
0.847166478	0.36556886
0.838972874	0.449641333
0.80349481	0.38468662
0.913778412	0.568685039
0.954046272	0.618481993
0.75346494	0.43371061
0.710198994	0.563934152
1.137153401	0.502215675
0.802939692	0.609281434
0.783085183	0.572539052
0.745650878	0.519823446
0.953379972	0.705335697
0.871817607	0.478465126
1.173224396	0.729314435

0.869481905	0.422134943
0.82584412	0.492694999
1.158278862	0.402215128
0.893297172	0.381937615
0.626361713	0.379967634
1.086776283	0.357514511
0.673517709	0.537026905
0.938459227	0.448652692
0.787616926	0.333622024
0.97438036	0.47672784
1.177974292	0.387600912
1.30934689	0.51037211
0.752778515	0.366546317
0.709352367	0.455936499
0.940771278	0.39967441
0.639832031	0.581586823
0.83708141	0.409044441
0.741755687	0.438849813
0.647056301	0.873250223
0.601458868	0.567899603
0.477456971	0.359537902
0.891994989	0.398068717
1.260946688	0.417284814
0.751676888	0.610020853
1.329533746	0.773652454
0.774084204	0.491256329
0.633342968	0.349067488
0.988145327	0.591173419
0.808545968	0.516511854
1.283389605	0.771181689
0.684580162	0.569732961
0.818442386	0.371345344
0.847068282	0.3294873
0.752594687	0.617514967
0.741581879	0.696507622
0.541392225	0.580080122
1.182937667	0.436550903
0.764460154	0.316220627
0.573337729	0.465834106
0.939426728	0.612369009
0.742926968	0.400189944
0.768909484	0.375704135
1.101905842	0.948007826

1.376886618	0.589627194
1.135840423	0.497112467
0.776392147	0.298373511
1.091098688	0.359969337
1.514841041	0.719151857
0.766890992	0.357514511
0.678876173	0.411130171
1.923111519	0.730747989
0.987798413	0.468353457
0.796268435	0.560186442
0.904447899	0.707201665
0.860566234	0.392928313
1.035007936	0.498301657
1.563040529	0.382414459
1.112231079	0.608879533
1.324661205	0.525914281
0.820374954	0.435501578
0.928246702	0.454000205
0.845360993	0.313703256
0.616854824	0.665857567
0.769483207	0.966022226
0.7606459	0.884039138
0.725647562	0.323964942
0.914842372	0.785401849
0.57266062	0.799681882
0.868483361	0.441437982
0.828893904	0.656493853
0.606944071	0.371690827
1.008684574	0.537282025
0.688080254	0.657644278
1.015422284	0.450459597
0.837783441	0.55035458
1.01047423	0.434246636
0.859674352	0.583957702
0.919739688	0.616337145
1.461141189	0.379523041
0.796350192	0.532693389
0.708601502	0.375360883
1.435656169	0.424739461
0.604205389	0.391826312
1.248396668	0.656811374
1.643967064	0.318332435
0.651283863	0.349826661

0.783812281	0.33834226
0.801730068	0.341906743
0.938768785	0.424159414
1.076742126	0.508225568
0.896688903	0.354179557
1.062152125	0.34185535
0.832510678	0.392340317
0.76285789	0.565719566
0.835680456	0.415967206
0.736233933	0.472948052
1.131401027	0.300311897
0.978563846	0.459316545
0.936361918	0.375704135
0.702032356	0.306756278
1.085670336	0.331581733
0.581211738	0.569882173
0.715361321	0.374890461
1.165170224	0.359811871
0.807130534	0.987842325
0.615105471	0.564543986
0.802732481	0.401604746
1.305679067	0.327206696
0.67876261	0.6629771
0.899292455	0.321480154
0.754082028	0.298608228
1.108565033	0.45605799
0.710577296	0.35150852
0.847720785	0.456961076
0.557640491	0.370196314
0.960771178	0.35253833
0.810275641	0.463427506
1.485298498	0.596980027
0.719150051	0.392428405
0.569225077	0.314336674
1.680768197	0.488309329
0.658218969	0.461302748
1.075473879	0.670884682
0.724295004	0.411924046
1.006186375	0.916928474
1.237398888	0.778401832
0.864853114	0.37050898
1.178993301	0.363351799
1.023715754	0.499036721

1.414543578	0.464563016
1.240690516	0.429156566
1.046227167	0.565812652
0.541757045	0.450743918
1.085670336	0.485009837
0.578469519	1.745825648
1.10759218	0.389867584
1.200435666	0.421405026
0.736233933	0.446388711
0.607340853	0.647465284
0.712131036	0.645457519
1.390509748	0.865341572
0.839668025	0.618394618
1.366007747	0.41044408
0.747935664	0.398763203
1.102423585	0.668723525
0.729222181	0.650534864
1.248986927	0.368663816
0.982773571	0.34498299
1.606174717	0.556757172
1.619101124	0.306314567
0.7142568	0.996246731
0.735055958	0.316705696
0.950503394	0.616366102
0.737049704	0.452650122
0.893297172	0.481011658
0.773549899	0.385596877
1.498076002	0.292800689
1.259511601	0.336000791
1.169793915	0.607661005
1.414849558	0.555890793
1.346804484	0.449232347
0.925047994	0.424035933
0.835057279	0.675056134
1.270638218	0.527262779
0.742827072	0.372522616
0.865616219	0.465100331
0.7646139	0.569303666
0.805593423	0.496222648
0.788922879	0.587117171
0.817594021	0.421894518
0.504006953	0.454845515
0.594891779	0.467588077

0.84119185	0.476224072
1.086962459	0.409965079
0.9995373	0.610735566
0.729994662	0.479072095
0.701876349	0.441813195
0.809023854	0.511610364
1.133800256	0.488355348
0.85652707	0.40768096
0.820374954	0.544286098
0.801450502	0.808130519
1.421825184	0.574465352
1.179991371	0.624584648
0.897285105	0.958659599
0.777463033	0.384410383
1.346170825	0.361665173
1.814415379	0.50560119
0.786289497	0.441245483
0.849212376	0.333594174
1.184612467	0.463449367
1.438825388	0.348293503
1.401344889	0.298443529
0.770083769	0.518744078
0.743425055	0.423382466
0.563002419	0.363491765
1.158792719	0.404234798
0.853148984	0.350609521
0.684139402	0.580774551
0.851776866	0.331740658
1.642964144	0.453077554
0.949853324	0.461105847
1.206312353	0.57702993
1.835676605	0.665196502
0.594440731	0.389297468
1.205242216	0.515071733
0.783352904	0.604410725
0.531463217	0.47925186
0.678673825	0.496696821
0.909064138	0.470712219
0.531724507	1.005587996
1.422238821	0.514243945
0.682734267	0.684835564
0.83786892	0.419188083
1.00969225	0.464706179

0.785316195	0.633411392
1.068574069	0.463884728
1.218132507	0.4060789
0.607340853	1.560747335
0.677835774	0.4511026
1.491762272	0.316220627
0.628283068	0.607194854
1.894969951	0.738799344
0.658487629	0.536583081
1.529694782	0.48731863
0.682351828	0.441158387
1.188824055	0.758319026
1.304751795	0.393374511
1.049355111	0.483841139
1.463264941	0.883202079
1.153433161	0.539627401
0.744061004	0.422069959
0.620824289	0.712906026
0.59632655	0.352976196
0.616217716	0.424609849
0.835156484	0.508508514
0.831913279	0.498071761
1.019796522	0.63466816
0.706212743	0.452007568
1.166995365	0.645705868
1.020911053	0.391513965
1.159061861	0.429664728
1.20972267	0.308063646
1.371986153	0.898518969
0.717196749	0.534457398
1.63163463	0.894320604
0.897034817	0.933991387
1.003415602	0.853917161
0.856708576	0.536899436
0.662610539	0.355071106
0.747636882	0.532877932
1.352797332	0.576001356
1.204746435	0.737248247
1.129555304	0.35406185
0.900803871	0.465169957
1.249826676	0.422255171
0.660071802	0.529809943
0.613234371	0.527868829

0.867174867	0.902091444
0.81122579	0.718815175
1.030238314	0.596036185
0.830694135	0.525818224
0.585445127	0.739685194
0.848834416	0.610020853
1.345119293	1.011593221
0.641725967	0.435201024
3.533922877	0.345434938
0.683206151	0.403486907
1.475106697	0.643353093
1.30400211	0.780523576
0.592493022	0.758319026
0.796175102	0.769068477
0.699544534	0.503296289
0.871916835	0.811271475
0.888481553	0.845065003
1.368569432	0.569352796
0.770655259	0.579069132
0.849965592	0.562520473
1.321919581	0.609281434
0.712588463	0.626085459
0.819501285	0.503319446
1.213230565	0.877138907
0.863401612	0.74315469
0.661157446	0.973157846
0.665811556	0.516890397
1.016459406	0.703017039
0.620978263	0.414177867
1.490638745	0.533373441
1.018852765	0.668595122
0.661527842	1.569832266
0.912099217	1.322782061
0.702076371	0.380800896
0.904898434	
1.285254094	
1.023685268	
0.926959101	
1.185287874	
2.412535159	
0.950218471	
0.874645912	
1.512691378	

1.519349129
0.804522297
0.665811556
1.010874006
0.963239395
1.222148328
0.640523521
1.007102088
1.355259423
0.907676201
0.600809881
1.232634084
0.886207512
0.773686948
0.960348533
1.648653327
1.05664294
1.359624534
1.446615376
1.415989646
1.455056928
0.86045842
0.669043651
1.563948848
0.735684504
0.835951693
0.969642594
1.312841924
1.267023539
1.752563975
0.727857618
1.054079419
0.972058864
0.662610539
1.0505887
0.723145329
1.311232634
1.721871513
1.240492481
1.399817468
0.950117951
0.810394723
1.1370109

0.907841401
0.861393701
1.394774837
1.378581153
1.276138817
1.283228477
1.131123042
0.887189706
1.799355731
1.177418644
1.347035418
1.533541817
0.764865252
0.897285105
1.16885281
1.607934909
1.101029543
1.134345615
0.768955441
0.876222075
1.358790294
0.953815095
1.388585475
0.776582696
1.290640394
1.329311267
1.188129243
1.035151229
0.95340873
0.656043194
1.326981001
0.974195257
0.963844282
1.138623858
0.866973232
1.023640762
1.900235902
1.516722364
0.610608193
1.152755229
0.786745775
0.945479366
1.258657463

1.134009612
1.6635381
1.438242937
1.30623643
1.393430987
1.376112825
1.418651781
1.06947666
1.404446252
0.787616926
1.558712412
1.628860415
1.546091259
1.212331876
1.417390823
0.690430066
0.913053296
0.710991626
1.132898745
1.004833714
1.070054287
0.308743262
2.729519135
