# Supplementary material for: The O-GlcNAc transferase OGT is a conserved and essential regulator of the cellular and organismal response to hypertonic stress
Source: PLoS Genet. 2020 Oct 2;16(10):e1008821. doi: 10.1371/journal.pgen.1008821 (PMC7556452; doi:10.1371/journal.pgen.1008821)
Supplement: S29 Table — (PDF) [file pgen.1008821.s036.pdf]

WT	ogt-1(dr20)	t-1(dr20);drEx465
4.240545033	2.29867815	8.02806254
4.680052451	1.55216012	8.8365011
4.498712087	2.42556167	9.82418752
4.582477235	2.85875673	8.88155153
1.937799281	2.71116481	7.59607689
2.598784327	2.09614679	6.05480874
4.670601114	1.87780634	9.08450088
5.093869575	2.78862666	10.2215649
7.566510101	2.73537881	9.16803552
7.32222084	2.32356598	7.57099997
6.076803742	2.01566889	8.24647678
7.816038582	2.32961378	7.30881583
3.188313219	2.16029619	9.68435159
3.271279057	2.39243225	1.95923968
5.519273926	1.3110614	5.19987299
9.727308544	3.20719246	11.8603973
7.121024865	1.35879337	12.2234724
3.292919429	1.88890698	9.65751671
6.591080584	2.22114597	10.5932527
3.338895728	2.2834052	8.30942752
3.508932622	1.58331142	2.68102827
7.557513062	2.53547628	5.70296819
5.95118772	2.49415153	10.0283175
7.656272466	1.70919891	9.67730401
5.827836602	1.95908504	12.8266045
3.576669808	2.58881221	8.74906705
3.515638349	1.93773469	1.97540115
2.894897683	2.12703424	10.8301501
4.326593241	2.23741504	4.56077127
3.624590782	2.71895844	8.99382493
4.887083729	2.09610121	9.09379449
3.536084447	1.77889619	9.52739289
9.070053546	2.76611341	7.87557376
4.365471964	2.09071375	7.46402235
1.521065573	2.12982489	0.37719394
5.699379493	2.60113307	9.99745361
4.534681773	3.12114583	0.98738775
4.139004169	2.25634472	
2.877685613	1.99153549	
6.614509297	2.32791722	
5.734704165	2.45263722	
6.599744768	2.33729642	

3.849059778	2.46996582
5.203108212	2.23554599
2.153607423	2.46240198
6.231977835	1.69155268
7.905309061	2.1570498
7.041672966	2.20844259
3.904610902	2.40053496
5.337383372	1.39678483
3.118107693	2.35363806
6.474236157	2.64531223
6.539853736	1.95249236
4.48900089	2.60038175
5.396306988	2.29949648
5.607182948	1.91591235
6.975339998	1.90427052
2.022483724	1.12787642
