# Supplementary material for: The O-GlcNAc transferase OGT is a conserved and essential regulator of the cellular and organismal response to hypertonic stress
Source: PLoS Genet. 2020 Oct 2;16(10):e1008821. doi: 10.1371/journal.pgen.1008821 (PMC7556452; doi:10.1371/journal.pgen.1008821)
Supplement: S30 Table — (PDF) [file pgen.1008821.s037.pdf]

ev(RNAi)	*hsf-1(RNAi)*	*ogt-1(RNAi)*
1.115874503	0.193101743	0.60513166
0.903531414	0.144504196	1.037508795
1.11279179	0.392176563	1.595264587
0.898626698	0.554974194	1.438394482
1.32826209	0.25378366	1.127667151
0.710630979	0.451615381	1.379231107
0.945155668	0.377843549	0.682318484
0.874678996	0.374391411	1.678727606
0.867201172	0.410882664	1.918100081
1.069433857	0.427678083	1.265082659
0.573721765	0.381720767	1.495097688
0.82510168	0.360447597	2.628134683
1.355273397	0.361457254	1.845476923
0.76283042	0.178631687	1.74069123
1.434153438	0.141223085	1.173606094
0.732240805	0.260004513	1.337714645
0.846541809	0.321263997	1.270818983
1.092620803	0.434657793	1.47059983
0.744100965	0.497631504	1.701800611
1.158070452	0.239406753	1.420052997
0.886912448	0.338109537	0.727082563
0.874575862	0.309614012	1.660877522
1.039871805	0.37515285	1.350519263
0.951017798	0.425214979	1.145253027
1.03393801	0.30015175	1.435591832
0.745762363	0.220111283	2.134863983
0.723389492	0.532653105	1.522129369
0.739259855	0.267127535	1.820952313
0.940876179	0.300531881	1.090899255
1.179146055	0.163497295	1.162730378
1.051873147	0.251891154	1.439347685
1.047102726	0.202034138	1.336202249
1.112940655	0.56121397	0.961745687
1.001068837	0.304699504	1.409966428
0.725302704	0.278869404	1.432958552
0.680862466	0.299916152	1.332984699
0.81703496	0.42093495	1.150615848
0.844619917	0.441981698	1.2608832
0.98226017	0.495376237	1.329878167
0.821340809	0.389977038	1.925841917
0.722475712	0.395246998	1.258394279
0.663383699	0.375347496	1.447769493

1.429706247	0.254962501	1.990077798
1.057473274	0.37833118	1.653490507
0.782606575	0.208146635	1.306229792
0.863918778	0.246290653	1.134660257
0.876547717	0.225956935	1.875878252
1.096181538	0.328175549	1.684819461
0.752228281	0.366423197	1.571843229
0.844337867	0.46880615	2.048308208
0.888918906	0.318437968	1.362595865
1.00436292	0.178396645	1.23469878
1.001850587	0.551407812	1.960646625
0.740817201	0.314052154	1.25619319
0.842262215	0.465663539	1.336950764
0.618174103	0.260747711	1.778604584
1.058623152	0.440011763	0.920283323
0.982666873	0.369392591	1.588230896
1.053717173	0.299258441	2.398206583
1.154440138	0.306691004	1.540996379
1.071089871	0.366933879	1.38151002
1.15209689	0.235494878	1.313858418
0.663920241	0.39979363	1.789268098
1.351272962	0.219423538	1.069720221
0.711748323	0.346158616	1.388012533
0.693134004	0.209136934	1.365759736
0.878378366	0.264172969	1.344310058
0.801040095	0.20363333	1.107402673
1.20548681	0.44101244	1.045484136
1.321157651	0.154608644	1.266941367
0.986936221	0.585304154	0.99033279
1.098529326	0.149827405	0.938752958
0.622106213	0.233451361	1.124440356
1.319022667	0.262549406	1.497474199
1.279648856	0.269032338	1.479002489
0.809371505	0.257822657	1.509559242
0.884386425	0.337418913	1.098334483
0.789630737	0.227233528	1.871596385
1.30683887	0.426786232	1.490379431
0.893505038	0.401057466	1.134780128
0.901412162	0.32713457	1.171385939
0.933040659	0.33344474	1.288414949
1.013500258	0.402433307	1.568416951
0.756166208	0.523163299	1.092759018
0.803674033	0.236115938	1.328024756

0.935603958	0.259597684	0.987008935
0.86027991	0.283160401	1.32686914
0.903888569	0.289438216	1.230712597
0.917041512	0.2261524	1.166779476
1.086766061	0.371121887	2.289306609
0.741456718	0.348529762	0.964248599
0.920841408	0.267206151	2.181679791
1.044417528	0.17487102	1.450576025
0.914131368	0.554722964	1.07683836
0.822319047	0.175788175	2.195259177
0.945999557	0.363931807	1.287624706
0.937982612	0.320338947	1.366202164
0.931259082	0.302542339	1.162276659
0.838935983	0.142372865	2.516438615
0.830907888	0.349999961	0.010982588
1.127068475	0.278813528	1.262997619
1.203548121	0.261944156	0.863528195
1.101965586	0.392916234	1.110928956
1.115635427	0.430164005	0.918977888
0.699293221	0.275317766	1.085058229
0.935047937	0.197510571	1.577763242
0.752895742	0.335769042	1.774413564
0.572349013	0.282311189	1.955044226
0.792661362	0.181172678	1.077495965
1.024362376	0.241999606	1.270487384
1.03540888	0.339412105	1.905731076
0.758212802	0.389307533	1.89458645
0.762289215	0.389307533	1.911999928
0.904963667	0.324752853	1.431204625
0.697066608	0.238969081	0.940775363
0.853175349	0.332704973	2.277292177
0.933377132	0.376525538	1.093773336
0.95110125	0.445243555	1.798531269
1.381556557	0.346940655	1.289512985
0.862135238	0.235859986	0.93475552
0.900364637	0.15545893	1.600249244
1.508830237	0.342645353	0.710775315
0.666023156	0.255426536	0.96049329
1.140441933	0.267886367	1.820540798
0.890568106	0.274116282	0.646350476
0.93446802	0.330800392	0.957087564
1.200816499	0.199728539	1.325770092
0.771285437	0.363361277	1.521116791

0.87909953	0.294447932	2.062908735
1.08799754	0.257339843	0.868857384
0.887069803	0.251534299	1.290130512
1.380490193	0.628835749	1.86268909
0.863327704	0.365410307	1.379955426
0.763231738	0.517587481	1.526009332
0.739276065	0.297225742	1.125057861
1.083333317	0.335169454	1.181509165
0.936936923	0.272959972	1.267052123
1.079150563	0.171718696	0.926472488
1.101660944	0.178757042	2.171995105
0.666023156	0.651186368	1.215290134
0.984373847	0.243061818	1.161877936
1.022073271	0.217476363	1.554056206
0.910296184	0.217476363	1.089217884
0.613348836	0.269160938	1.574127042
0.684766166	0.25041396	1.013438645
1.068614413	0.288939185	1.563969432
1.009714406	0.30236016	1.288882232
0.883155156	0.353825719	1.160672043
0.939463748	0.309387188	1.184247706
0.859059193	0.322899281	1.371343055
0.795581913	0.355189209	1.595368182
1.172213776	0.297067339	2.137517868
0.86032066	0.213937949	1.126487648
1.065310568	0.427875899	1.870828526
0.636639782	0.307675085	1.308891459
0.789433329	0.275481743	1.336804517
0.926612482	0.537845308	1.692811224
1.064196292	0.460036506	1.781134155
0.724722391	0.164298752	
0.726869716	0.467525172	
0.869255742	0.502423639	
0.61661491	0.344442917	
0.740794302	0.172562491	
1.310306684	0.285391812	
0.840528481	0.186205252	
0.934873514	0.106403001	
0.944777243	0.414780562	
0.996545859	0.14049019	
0.789471395	0.241322007	
0.638479598		
0.695602029		

0.998037694
1.33703899
1.117059922
0.822734487
0.921281653
0.810210263
1.188652269
0.874176512
0.80828381
0.834640891
0.773141036
1.033892654
0.947345914
0.887012491
0.534790416
1.221723458
1.009249813
0.935433445
1.378766831
0.969445108
0.797240681
1.130314841
1.099041307
0.680135275
0.715931868
1.293151937
1.856948283
0.993355467
0.800948778
1.169677313
0.747414442
0.877986844
0.775639467
0.739563213
1.017825356
0.909257318
0.829130868
0.679615466
1.148087086
0.877192703
0.819397574
1.136115691
1.04781987

1.056229339
0.677444775
1.264121246
0.796005095
1.067717252
0.940204488
1.038434808
0.958915025
1.049491034
1.285839828
0.838051976
1.059335363
1.122372356
2.053389425
1.367100163
0.860599938
0.893882808
1.062046826
1.232804452
1.739305505
1.249207612
0.933316032
1.483733178
0.710720218
0.758741854
1.322795991
1.113003638
1.169910022
1.163200644
1.095063368
1.03179304
1.184666188
1.090848096
1.124839108
0.835189997
1.10205544
1.024738887
0.789827805
0.756599222
1.586100363
0.992254394
0.748248237
1.471244758

1.181144269
0.84242929
0.97243381
0.998632547
1.286196892
1.343348524
1.08622322
1.308996331
1.018313284
1.213533965
1.543851471
2.08919204
1.291150154
1.779853153
1.097376557
1.627915108
2.559267483
1.246658728
2.356697322
1.229581212
1.500314068
1.858648621
