# Supplementary material for: The O-GlcNAc transferase OGT is a conserved and essential regulator of the cellular and organismal response to hypertonic stress
Source: PLoS Genet. 2020 Oct 2;16(10):e1008821. doi: 10.1371/journal.pgen.1008821 (PMC7556452; doi:10.1371/journal.pgen.1008821)
Supplement: S31 Table — (PDF) [file pgen.1008821.s038.pdf]

zcIs4 no DTT	zcIs4 Dtt	(dr50);zcIs4 no·1	(dr50);zcIs4 Dtt
1.135247819	10.16952401	1.054920557	4.437989055
1.823962338	2.478004606	0.960917216	0.775941456
1.009121984	13.92920838	0.971486425	1.216712398
1.014764791	2.466727686	1.072509447	1.029202735
0.76178355	4.110836748	0.997448262	7.717430191
1.072325605	3.723420157	0.9808968	9.717297426
0.451962663	1.930253656	0.927405228	1.008905072
0.855500755	2.248436028	0.815557361	1.31790211
0.984632944	2.024449953	1.053869577	2.847559685
0.905643815	2.054730187	0.586075094	1.252708596
0.749622254	2.466909063	1.057072828	1.088860441
1.047971724	3.196412996	0.859207721	9.863803925
1.181241995	2.064704605	0.979130319	1.353527548
0.954316852	2.131770191	1.255635006	6.277080316
0.790544013	3.192494323	1.27390828	9.690125464
1.055080059	2.61400206	0.93154543	2.330577235
1.653368897	1.934313441	1.158219324	9.804657261
0.942420271	2.111122782	1.072904988	5.969593453
0.862265721	2.424267921	0.736815594	0.998641667
1.078883694	2.600738744	0.73995098	13.03007708
1.08417234	2.115150361	0.811285571	7.786682981
1.021459767	1.932243224	1.000437139	8.492721868
0.870752588	15.01943556	0.769853761	12.19374535
1.08951309	2.085179114	0.916053351	8.166583838
0.988942651	3.051274328	1.116054428	4.661346207
0.839741656	1.950289755	1.260061451	1.392151956
1.041279653	1.877314863	0.766028547	11.44729511
1.043339554	3.320751642	0.900643257	7.741959285
1.04540762	2.185570437	0.915891168	1.967329986
0.828673093	3.321442376	0.881492593	1.117780978
1.101449987	4.078216535	1.013926999	12.81841873
1.000774468	2.158118302	1.184257527	4.289130037
1.154586112	1.814737701	0.870625316	13.62942381
1.158053338	2.458324438	0.854628835	1.850200121
0.923327599	3.507869268	1.063538106	3.464095544
0.929869394	2.115150361	0.970805965	2.02035731
0.723231751	2.314440236	0.956166723	15.57025466
1.000774468	2.279679805	0.900877268	8.044718703
0.949010271	2.251742552	0.843374463	11.70297073
0.984518233	3.517144884	1.386486221	1.176103422
0.986521322	23.78326299	1.039864666	16.33787369
1.124980454	1.659821068	0.906121024	15.11655116

0.747258566	3.060779855	0.889949943	14.76002079
0.800210259	2.372027945	0.75628981	1.458388149
0.940338753	1.746219379	1.049628653	6.487402994
0.907368851	2.024351391	1.00071231	1.669172312
1.035889361	1.615775537	0.989295558	1.380067304
0.861203648	2.166132535	1.188574711	1.053570268
1.039106409	1.453386036	0.897875113	
1.08104492	1.875467112	0.720035115	
0.835547335	2.328117446	0.866319621	
0.928315456	14.77112082	0.847203314	
0.950155738	2.451359395	1.071700617	
1.004997145	2.005617124	1.09325625	
1.022943522	1.605015656	1.684493915	
0.897318879	2.450649943	0.874833819	
0.900167511	1.522917668	0.77405989	
1.026190962	2.070385274	0.957389864	
0.703619801	2.996637184	1.18437342	
0.847952067	2.482026863	0.736035895	
1.030553091	1.788446016	1.022272077	
0.892553674	2.360465433	0.86190077	
1.168286074	1.896045603	0.964508004	
0.949232435	1.796421249	0.946331865	
0.876214555	2.047038684	0.989936198	
0.844234772	1.885115341	0.977836632	
0.809274921	2.05763954	1.126301779	
0.905152151	16.36592594	0.840650134	
1.108349573	2.149512902	0.945731401	
0.998598871	1.957679369	0.979173717	
0.968986451	22.16597861	0.982964067	
0.913083386	4.975995612	0.769276226	
1.070364745	6.793653297	1.027026831	
0.959813566	1.734351869	0.941441261	
1.09276149	2.378740528	0.646065318	
1.281168643	2.199978355	0.842074965	
0.759516337	2.471186115	0.951273546	
0.836401454	1.947010817	0.952517041	
0.894428187	1.770155995	0.934533765	
0.879332173	1.973176463	0.782400361	
0.86215368	6.561391646	0.935761799	
0.938789562	1.812878339	0.892237994	
0.866054828	2.291171346	0.699136998	
0.771572152	2.820117182	0.814826972	
1.180663394	15.16892032	0.771808612	

0.799014667	2.453309196	0.892765911
0.956015059	3.3578617	0.873980376
0.88100399	2.475460654	1.063330545
1.018049056	2.585039349	0.998185666
1.234826561	2.054221276	1.091921383
1.632477195	2.05763954	0.754908611
0.866411229	1.733825829	0.816039797
1.202552865	2.556891992	0.888043308
0.631579246	1.747928008	0.723179373
0.812983454	13.47966225	1.013926999
0.734524084	9.340561697	0.780479889
1.096980219	3.956550221	0.928186859
1.077035125	2.074776335	0.905095839
0.958488222	2.132198343	7.918214213
0.800619574	3.968218331	1.842933772
0.780604085	9.208156828	0.879792906
0.841640718	3.83996222	0.025957379
1.342617336	3.508965477	0.85793823
1.042031366	4.359919965	0.768421475
0.744074634	6.268008508	0.996341273
0.906026589	14.22827271	1.087325754
1.087231907	1.917668994	0.411083878
0.889049052	8.637453482	0.487979599
0.949666033	23.96220556	
0.929460373	3.79460319	
1.203569294	2.195247219	
0.856778141	1.896372001	
0.958775539	2.873537412	
1.027365094	3.552199484	
0.824880773	3.167968832	
0.824880773	4.627581138	
0.88782298	2.695592527	
1.095623081	1.763559885	
0.895429787	3.351078141	
0.959075531	15.67002127	
1.045034763	2.423527913	
0.945175886	2.549657022	
0.906525571	3.654236348	
0.994550746	2.381843233	
0.854932962	2.249336844	
0.816288265	4.995144654	
0.84739907	44.67871959	
1.434575258	1.986567281	

0.786606087	16.02073127
1.61057971	
1.575716041	
0.867785646	
1.530043112	
1.496421903	
1.335268775	
1.23014706	
0.937364515	
1.127944704	
1.088464025	
1.561943202	
0.608518603	
1.752102165	
2.075401268	
0.662102905	
0.840156343	
0.711577283	
