# Supplementary material for: The O-GlcNAc transferase OGT is a conserved and essential regulator of the cellular and organismal response to hypertonic stress
Source: PLoS Genet. 2020 Oct 2;16(10):e1008821. doi: 10.1371/journal.pgen.1008821 (PMC7556452; doi:10.1371/journal.pgen.1008821)
Supplement: S32 Table — (PDF) [file pgen.1008821.s039.pdf]

drIs4		frIs7	
ev(RNAi)	*ogt-1(RNAi)*	*ev(RNAi)*	*ogt-1(RNAi)*
4.277312621	0.889041153	0.766102825	4.063554451
4.069411746	1.420946116	1.544562148	2.692059286
5.637443913	0.956601224	1.149154238	0.920738729
2.450352731	1.125744306	0.851225362	0.823275166
4.484035848	1.281571018	0.909747105	1.408944381
4.207670002	1.178039266	1.625066599	2.179191271
2.479199546	1.065682919	0.840844565	0.872588622
3.940516351	1.336951566	1.044685671	1.603710222
3.705600953	1.304456215	1.419931961	0.908769126
3.972423366	1.027961978	0.685275463	1.024927586
4.072970682	1.051869203	1.266540961	1.246311944
4.479258821	1.133584954	0.919323391	1.133675771
5.313726595	0.969630636	1.079508527	0.912277887
3.354763921	1.477933121	1.266017381	2.161144713
3.959103693	1.420669031	0.985794077	1.153992541
5.204307452	1.224123134	0.970746731	1.483704695
3.659922308	1.159516636	0.935358101	1.115844027
5.211408075	1.221042008	0.649521961	1.298241608
4.965791766	0.93194795	0.61090096	1.298241608
2.131095578	1.11313597	1.070290712	0.712760099
3.25267474	1.254558983	0.957628532	0.809049118
3.392891722	1.138044105	1.064031702	0.810102764
6.556203929	1.166337322	0.978446543	1.357793976
4.433080895	1.125075547	0.864954803	1.517425257
6.254424209	1.194921724	0.78494142	0.906708425
4.556222031	1.079549971	0.718221399	0.713023889
4.474511558	1.396319329	1.169053013	1.157468422
5.715377022	1.178777709	1.316739231	0.846045093
4.794294894	1.458746735	0.864954803	1.347231858
3.643362659	1.29385088	1.36804076	1.117930274
6.104570413	1.201225867	0.757684113	2.131075848
4.003313425	1.176128622	1.028563979	0.896966929
2.674558609	1.546022181	1.395401575	1.171583891
4.083209144	0.88738518	0.337085243	0.865494406
4.196394036	1.147181352	1.363403333	1.143689036
4.186798623	1.27264021	1.197035665	0.723610077
2.884457969	1.039047375	1.461643549	0.865494406
5.599681131	0.973020953	1.033630796	1.591392939
3.904952124	1.114126305	1.523499937	1.382543012
5.014353746	1.183745341	0.605846622	3.977152864
5.176756775	1.284028489	1.102723764	0.920272533

3.870977827	1.127603334	1.171467913	0.998647391
4.0701386	1.079983004	4.266901813	0.700638328
4.883725052	1.172844413	0.851225362	1.048579761
3.389395183	1.36822963	1.407127639	1.122208848
4.49015919	0.983651645	0.922805676	3.806708445
2.482895883	1.220168275	0.644385554	1.298241608
3.489872194	1.019420795	0.903829176	1.116766975
3.17972359	0.891301044	0.909747105	0.944175715
3.77232358	1.104301558	0.914749642	1.217101508
4.943556877	0.974383727	0.843625135	0.971203646
4.845226738	1.038373107	1.099499425	1.180219644
4.381231996	0.969431941	1.498896832	2.865084929
5.099491749	1.055988365	0.685709319	1.353880534
4.752745232	1.023787264	1.074412499	1.017821421
4.271877954	1.004635352	0.995933673	1.892763466
2.223318859	1.267186038	0.881844979	1.20932095
5.030446406	1.184187202	1.009392236	1.324206441
4.628543016	1.256143022	1.234276774	0.877684468
2.798871427	1.349885039	1.312305766	5.808429122
4.066814952	1.326269864	1.307238948	1.162604425
4.499643671	1.155664421	1.036122674	0.933822911
2.344216526	0.802742286	1.006321508	1.259679976
4.645917426	1.186128493	1.5808471	1.123478315
5.460429801	1.226251406	0.823766479	4.874100074
2.67392181	0.951531589	1.142434038	0.710358616
2.163000823	1.07193985	1.035274088	0.858986929
3.551765859	0.979147381	1.582168879	0.643376372
3.400475707	1.15265559	0.502996805	1.04368443
3.232965073	1.226251406	0.905864827	1.01754072
6.008204876	1.053503686	1.276838042	0.798917913
3.643628133	1.365652926	1.157134476	1.369054787
4.723342144	1.201890998	1.205902596	3.491822257
2.506663543	0.952561344	1.047406207	2.050725598
3.608683395	1.034437582	0.985794077	8.129176426
7.644222826	1.125075547	0.93174668	0.649120804
4.935801409	0.980209939	0.740566065	0.806674397
2.678020821	0.913362266	0.66043347	1.146195294
3.855955915	0.970758114	0.915067264	4.528013415
3.644977625	1.162858182	1.276838042	1.266577179
3.733120754	1.185869286	0.760022644	1.127420344
3.403173212	0.91540787	1.049455925	1.697142661
6.504979374	0.946370195	0.544533871	0.865494406
5.965001816	1.123506802	0.872775371	0.963474904

2.584468214	1.396407131	1.040386553	5.818904352
4.883902681	1.014577056	0.883964799	1.006799615
5.566761195	1.262761268	0.803935064	8.483559025
5.683437045	1.00744888	0.766102825	2.396753739
4.053102533	1.001400731	0.995933673	4.021620622
5.371470282	1.204113429	0.766102825	4.147160694
5.137635243	1.216542044	1.276838042	0.841452894
3.384802507	1.260153929	1.154787347	2.409536425
5.522808631	0.975801013	1.149154238	8.839712525
3.222118966	1.189689931	0.630337768	0.889535917
5.024158348	1.594335374	1.264069662	3.894724825
5.636563338	1.205333402	1.222995474	1.074406848
6.596424372	1.173831656	1.11029395	0.676548444
3.377585444	0.942107266	0.908935556	3.573982781
7.010453509	0.882168276	1.360840545	2.596483217
4.508583489	1.000845938	1.121793423	7.257639835
4.855279076	1.290683676	1.124833514	3.644186971
7.982307655	1.102357365	1.001826772	1.008046425
5.340699826	0.986822669	0.713527141	1.221874455
3.799783624	1.137964282	5.703209923	0.900114182
5.183294585	1.019068142	1.14383408	9.683355056
2.970090683	0.98040431	1.130315644	3.916362185
4.728619622	1.227723497	1.35921469	4.247227514
3.639504686	0.984063461	0.721037953	3.318982895
3.315800344	1.227723497	1.305857089	4.285252789
2.62002417	1.133460082	1.165808647	0.919587806
3.584193064	1.236279061	1.350099242	1.983140725
5.910774527	1.034758517	0.957628532	1.231665116
3.655347405	1.079908031	1.318701585	4.710762408
3.968266466	1.108419292	1.185635325	4.85429471
6.139368017	0.911048785	1.149154238	0.876313086
4.237913812	0.855191671	1.155758573	3.051341591
6.244202834	0.996901498	1.029093348	4.678568815
3.932272593	0.961408266	0.840367895	2.540037929
5.343578991	1.182431984	1.485975308	0.941225166
3.755464478	1.092102878	1.641648912	0.857091547
4.153517235	0.971785371	4.935470126	4.679708123
4.349437859	1.279208675	0.957628532	0.830874629
3.341860983	0.956601224	0.780289915	4.18322296
3.329056917	1.227723497	0.901297442	4.909713719
4.21246748	1.039851218	0.771077519	4.989320691
3.297332897	1.204113429	1.042125167	6.075770727
3.958427789	1.118105327	0.943335569	0.699053174

4.293675836	1.278878642	1.34475496	3.917907711
5.556835932	1.320968319	7.394346891	5.159245872
3.997040151	0.849722115	0.796230465	6.086380014
3.484965328	0.993871402	1.185635325	1.808786061
3.621798117	1.066755305	0.766102825	3.864057701
5.181692206	1.240601715	0.659699655	6.040797688
3.770247126	1.183842159	0.870571393	5.802746583
3.96454389	1.059945955	0.877080338	4.956922505
3.628396244	1.185121104	0.919323391	0.927315435
4.067629782	1.251057423	0.754495207	4.321289925
4.096304207	1.097177032	1.485975308	4.268461046
6.612052861	0.909424812	1.200608906	5.352399614
4.336182952	0.878541615	1.072543956	8.463536639
3.127545745	1.149433882	0.957628532	4.057005026
4.100776586	1.063312199	0.957628532	5.855156784
5.520827596	1.142589385	2.41927208	2.88233458
4.152196982	1.048024651	0.790043539	4.447679584
4.513682366	0.91717393	0.897019131	2.737596435
5.309078916	0.885074828	0.904426947	3.516496955
4.92953923	0.89843342	0.725194422	3.404822331
7.979545611	0.993871402	1.316739231	5.261294939
4.515684887	1.065093457	0.794130978	3.071109181
4.539075797	0.701556284	1.006321508	6.104314847
4.619915722	1.019068142	0.731671238	4.529203807
4.207080693	0.783898571	0.744822191	6.026699577
6.592786972	0.826395834	0.710498588	2.332026593
4.306039361	0.997184307	1.068124132	7.78944965
5.431522538	0.971283416	1.197035665	6.690208581
4.761457258	1.057667201	1.860535433	5.717256314
3.402299484	1.467513242	0.700703804	5.304643131
3.734326542	0.77301109	1.255906271	4.763795654
3.979300961	1.086129507	1.399610931	6.272692128
5.910774527	0.978674592	1.159944419	4.691890725
3.885568912	2.029154112	4.300294162	9.866636224
4.082261544	0.895990128	0.718221399	4.609260904
3.739469599	0.99103986	1.054779252	5.211646169
3.425836665	1.519366626	1.347773489	3.599085647
3.808488742	0.749586512	0.870571393	5.484706121
5.803305899	1.520985384	0.845697924	5.413943729
5.197089677	0.661414419	0.712082754	3.573982781
2.987627852	1.303437904	1.097769293	4.828973459
2.336817836	1.024929883	0.912027173	5.460645116
3.659050898	1.209930402	1.154787347	3.978783634

3.661541742	0.446570596	0.748491266	4.841359331
5.554089857	0.85814447	0.882520412	3.955108156
3.070532222	0.650825466	1.316739231	5.292831173
4.359530533		1.165808647	7.181762089
3.228192242		0.957628532	7.23132709
3.64899856		0.990650205	5.027872125
6.445906377		0.972828985	5.52920167
3.931601156		0.722146106	7.956196279
3.058183342		1.064031702	2.967409391
4.728619622		1.01625885	14.48564321
4.766148349		0.925707581	5.088058021
3.646705922		1.006321508	9.429333787
2.551645834		0.679607345	24.66659056
1.91126841		0.972828985	2.959990867
3.089170226		0.849217755	7.052176638
3.17229642		0.825033812	7.70290021
6.09664794		0.807999074	6.715042802
2.72956033		0.770774184	7.547916328
3.450614318		1.172022979	4.81370484
3.208706172		0.976781102	6.95379988
5.153363721		1.004725017	3.0941425
3.673257356		0.863214451	6.191613825
4.426898077		0.809263548	5.374116426
0.756438407		0.735149176	8.160375824
1.426738679		1.404521847	5.810163264
5.069383135		0.974429032	4.363162519
1.555466981		26.12084634	4.793507477
2.835977677		0.722738515	4.956922505
1.370225004		0.589309866	7.810221516
4.252228839		0.978003607	6.750856364
3.456907526		0.976781102	5.243877869
4.601676813		2.058186695	3.437936111
1.709488711		0.678825288	5.977949732
6.133822622		4.929705138	7.587500956
1.394050596		0.844011587	5.726490382
3.399806401		0.97503996	5.321187827
4.095046404		0.986647578	8.425323091
1.703104525		0.772281074	3.617766615
8.607924068		0.866425815	7.820360165
		0.721330582	
		0.957628532	
		1.266017381	
		1.585040329	

1.231236684
0.736637332
1.044685671
0.618468427
0.585217436
0.845697924
1.828199924
0.688631753
0.780289915
0.700703804
0.766102825
1.276838042
0.994460398
1.120244698
9.786496459
1.20963604
0.595031321
1.354694021
0.957628532
0.646710177
1.42276239
3.412639859
0.859911335
0.732304171
0.655909953
1.340679945
1.077332098
0.844011587
0.940217104
1.021470434
1.330941349
1.197035665
0.739985684
0.745942225
0.985794077
1.00322989
1.362779065
0.743570389
0.874356486
1.249080694
0.661017925
1.735701714
2.340869744

0.753543763
1.824054346
0.734181874
1.56057983
3.075201482
0.980985325
1.993430821
0.820824456
4.015861585
0.90722703
1.17527138
0.831624778
1.444843048
5.453613334
0.97332736
1.723731357
17.23731357
1.454176659
6.824479192
0.696457114
1.915257064
0.868959223
0.810301065
0.940828031
6.478075362
1.144179545
2.698771317
16.16162125
4.305728286
9.132506243
0.616404572
0.446413301
7.531180318
5.366709897
4.259303022
6.36454655
4.259303022
1.297432204
6.396235854
0.74194643
6.027426641
1.031292265
2.944707735

4.095703567
7.49002316
9.659557364
10.10830117
0.749448416
0.993096255
9.427044508
6.52485881
1.053391385
1.154401518
8.485316105
0.658369616
6.303377678
9.547699392
0.702260923
0.68402038
0.876473571
1.033230784
6.296407597
10.33078053
5.918144327
6.874764828
7.011506294
4.964792582
