# Supplementary material for: The O-GlcNAc transferase OGT is a conserved and essential regulator of the cellular and organismal response to hypertonic stress
Source: PLoS Genet. 2020 Oct 2;16(10):e1008821. doi: 10.1371/journal.pgen.1008821 (PMC7556452; doi:10.1371/journal.pgen.1008821)
Supplement: S33 Table — (PDF) [file pgen.1008821.s040.pdf]

gpdh-1 mRNA

	50mM NaCl			250mM NaCl		
WT	0.850667161	1.122462048	1.047294123	17.95939277	15.63455949	20.62992494
ogt-1(dr20)	0.890898718	0.831237896	1.350349946	92.6252312	114.0350359	130.9918582
