# Supplementary material for: The O-GlcNAc transferase OGT is a conserved and essential regulator of the cellular and organismal response to hypertonic stress
Source: PLoS Genet. 2020 Oct 2;16(10):e1008821. doi: 10.1371/journal.pgen.1008821 (PMC7556452; doi:10.1371/journal.pgen.1008821)
Supplement: S34 Table — (PDF) [file pgen.1008821.s041.pdf]

*gpdh-1* mRNA

| | 50mM NaCl | | | 250mM NaCl | | |
|---|---|---|---|---|---|---|
| gpdh-1(dr81) | 1.021012126 | 0.888842681 | 1.101905116 | 38.58585049 | 45.88656794 | 37.53071838 |
| gpdh-1(dr81);o | 1.040059934 | 0.983956654 | 0.977159968 | 71.83757109 | 76.46176865 | 137.8224087 |
