# Supplementary material for: The O-GlcNAc transferase OGT is a conserved and essential regulator of the cellular and organismal response to hypertonic stress
Source: PLoS Genet. 2020 Oct 2;16(10):e1008821. doi: 10.1371/journal.pgen.1008821 (PMC7556452; doi:10.1371/journal.pgen.1008821)
Supplement: S35 Table — (PDF) [file pgen.1008821.s042.pdf]

gfp mRNA

	50mM NaCl				250mM NaCl		
gpdh-1(dr81)	1.023373892	0.848703971	1.15135548		36.08514967	29.92611993	34.855957
gpdh-1(dr81);o	0.810377861	0.766664172	1.609560345		63.41123925	51.50593104	117.5121946
