# Supplementary material for: The O-GlcNAc transferase OGT is a conserved and essential regulator of the cellular and organismal response to hypertonic stress
Source: PLoS Genet. 2020 Oct 2;16(10):e1008821. doi: 10.1371/journal.pgen.1008821 (PMC7556452; doi:10.1371/journal.pgen.1008821)
Supplement: S36 Table — (PDF) [file pgen.1008821.s043.pdf]

Concentration	WT					ogt-1(dr15)					ogt-1(dr20)				
50	100	100	100	100	100	95	100	100	100	100	100	100	100	100	100
200	100	100	100	100	100	95	95	100	100	100	100	100	100	100	100
300	100	100	100	100	100	100	95	100	100	100	100	100	90	95	100
400	100	100	100	100	100	95	100	95	100	90	95	100	100	90	95
500	70	80	88.24	90	85	85	85	95	100	65	90	70	65	65	75
600	0	0	5	0	0	10	5	0	5	0	55	0	5	5	10
