# Supplementary material for: The O-GlcNAc transferase OGT is a conserved and essential regulator of the cellular and organismal response to hypertonic stress
Source: PLoS Genet. 2020 Oct 2;16(10):e1008821. doi: 10.1371/journal.pgen.1008821 (PMC7556452; doi:10.1371/journal.pgen.1008821)
Supplement: S37 Table — (PDF) [file pgen.1008821.s044.pdf]

	50 mM NaCl									
WT (drIs4)	100	100	100	100	100	100	100	100	100	100
ogt-1(dr20)	100	100	100	100	100	100	100	100	100	100

	250 mM Na			
WT (drIs4)	42.8571429	100	100	100
ogt-1(dr20)	0	23.9130435		6.52173913

aCl					
100	16.6666667	100	100	100	0
6.25	0	0	0		0
