# Supplementary material for: The O-GlcNAc transferase OGT is a conserved and essential regulator of the cellular and organismal response to hypertonic stress
Source: PLoS Genet. 2020 Oct 2;16(10):e1008821. doi: 10.1371/journal.pgen.1008821 (PMC7556452; doi:10.1371/journal.pgen.1008821)
Supplement: S38 Table — (PDF) [file pgen.1008821.s045.pdf]

	Unadapted					Adapted				
N2	0	0	0	0	0	35	55	70	30	75
ogt-1(dr20);drIs4	0	0	0	0	0	20	10	0	0	0
ogt-1(dr20 dr36);drIs4	0	0	0	0	0	10	40	60	35	55
gpdh-1(dr81)	0	0	0	0	0	35	50	10	45	75
ogt-1(dr84)	0	0	0	0	0	45	31.57894737	75	80	70
