# Supplementary material for: The O-GlcNAc transferase OGT is a conserved and essential regulator of the cellular and organismal response to hypertonic stress
Source: PLoS Genet. 2020 Oct 2;16(10):e1008821. doi: 10.1371/journal.pgen.1008821 (PMC7556452; doi:10.1371/journal.pgen.1008821)
Supplement: S39 Table — (PDF) [file pgen.1008821.s046.pdf]

osm-11(n1604)	lr52);osm-11(n1604)
0.933582723	0.078637656
1.715458253	0.212315286
0.511832633	0.282078185
0.653459049	0.227430863
0.7182882	0.238562162
0.625047786	0.197688066
2.126330786	0.174292706
1.074848529	0.166004905
0.698651544	0.116524397
1.081134193	0.337632828
0.802455409	0.265692761
0.682674066	0.171539878
1.302007239	0.238501086
1.190601448	0.159934715
0.992167873	0.29987759
1.314622432	0.264632905
0.885528618	0.253040725
2.168890782	0.231953998
1.161101806	0.217723691
1.717053625	0.209745636
1.074848529	0.245220098
0.69142303	0.145409318
0.902872765	0.344135386
0.664232237	0.260708626
0.667984962	0.158668979
1.299443744	0.260244731
0.901718565	0.195894911
1.035332039	0.164605574
1.444327711	0.237074098
0.975325517	0.518733486
1.239543062	0.135767698
0.710280022	0.232064981
0.931535392	0.340460573
1.139339441	0.18672941
0.96869065	0.254323026
0.86313594	0.187030101
0.933251717	0.198851815
1.192640149	0.152662582
0.74841305	0.225646807
0.687903059	0.16839365
0.642748618	0.32033879
0.870997946	0.237965958

0.911992691	0.250344389
0.680479644	0.228813421
0.617636841	0.176479685
0.857068762	0.1878992
1.170197996	0.252333907
0.562714818	0.175886952
1.148976014	0.118452397
1.029248895	0.266193832
1.11917218	0.210408864
1.057512263	0.244200874
1.249511415	0.159860188
0.921298739	0.348585412
1.156139594	0.183504732
1.380766957	0.170825327
0.533768317	0.139744242
1.926615288	0.228633253
0.806136397	0.216165443
1.029686826	0.170775339
0.454466157	0.204564173
0.575811712	0.106360524
1.777082902	0.238003469
0.984114562	0.172598766
0.806136397	0.221779857
0.964418886	0.156832533
0.92659356	0.137220156
0.489152624	0.170702076
1.135832843	0.115481673
0.98159683	0.151746815
1.061412923	0.098235762
0.864236317	0.152903756
0.740994062	0.180671078
0.98465145	0.12104329
0.790329801	0.180147014
1.143875499	0.183405487
0.81739978	0.218654485
0.877427371	0.137874754
1.701843505	0.174969628
0.711053642	0.187262725
0.58220962	0.178050179
1.144757377	0.143846399
0.483681838	0.197067057
1.130684816	0.108056865
1.030524879	0.192039836

1.24503288	0.230148908
0.737819753	0.223093086
1.084035269	0.204481853
1.20492574	0.155936347
0.931152202	0.19010004
1.26816661	0.213705869
0.445000983	0.136321236
0.80851438	0.190581305
1.794303593	0.211906026
0.777198167	0.212124736
1.428223388	0.223688001
1.030063174	0.190018364
1.187332678	0.227679661
1.1797118	0.222664691
0.986099568	0.143635274
1.261778708	0.19244413
0.903633719	0.201641828
0.376196985	0.239665715
0.892327081	0.231629587
1.288237575	0.214040516
0.783103928	0.205790836
1.460392023	0.188011028
0.909956993	0.196648792
1.265014038	0.241761778
0.418397414	0.199152422
1.018277554	0.353662624
0.838618083	0.240555163
0.946508406	0.149364317
2.342618589	0.131149156
0.618037904	0.20983865
0.637974611	0.183608819
0.97161449	0.175209378
1.027528154	0.145985034
0.466294583	0.26327785
1.253989951	0.194019628
0.704211105	0.330619934
1.176010744	0.264842997
1.863833088	0.36754966
0.659031215	0.13101601
1.33360836	0.19026716
1.732402924	0.305084562
1.074848529	0.173693762
1.093595887	0.280215873

1.521324072	0.166201749
0.758716609	0.150447788
0.721913191	0.332244529
1.074848529	0.278344797
0.569037457	0.286201304
1.401141833	0.235248799
1.048844129	0.210233083
1.074848529	0.334967845
2.525894044	0.190800869
1.184323842	0.174116118
0.831216196	0.159041442
0.638011694	0.292002293
1.356117303	0.257942805
0.872809332	0.20983865
0.615924438	0.210140142
0.635952046	0.280215873
2.222568145	0.187657719
1.400131637	0.161592069
1.036461082	0.283006074
0.570823513	0.134427885
0.77896326	0.237299929
0.745907816	0.118796736
0.319993074	0.162328012
0.969471222	0.117318882
0.451747933	0.134955053
0.721684012	0.363318608
1.204870529	0.129792441
1.247355083	0.278813391
0.531246974	0.133385808
1.774514081	0.313665065
0.705369347	0.121418735
0.846157353	0.254839593
1.573578247	0.206538208
0.650964602	0.134427885
1.189381569	0.214488814
0.505811073	
0.286626274	
0.373860358	
0.954304769	
0.759874601	
0.85436678	
0.986849234	
1.142026562	

0.330722624
0.833556002
1.104914222
0.470246232
0.835993301
2.197116847
0.982189173
0.838184633
0.64153263
1.976628227
0.755889413
1.201740369
0.310511797
1.426845708
0.806136397
0.930573559
0.934385369
1.100440161
