# Supplementary material for: The O-GlcNAc transferase OGT is a conserved and essential regulator of the cellular and organismal response to hypertonic stress
Source: PLoS Genet. 2020 Oct 2;16(10):e1008821. doi: 10.1371/journal.pgen.1008821 (PMC7556452; doi:10.1371/journal.pgen.1008821)
Supplement: S40 Table — (PDF) [file pgen.1008821.s047.pdf]

WT	*ogt-1(dr20)*	*ogt-1p*	*dpy-7p*	*nhx-2p*	*myo-3p*	*rab-3p*
8.46276955	1.73576503	6.30574371	6.12160625	3.64941702	2.57115205	2.11033247
4.83368674	1.63580817	5.87670214	7.70354562	3.93897664	2.18665069	1.72164368
9.01367057	1.74099463	8.36692724	7.47835193	3.3890769	2.3848032	2.37878229
7.38791049	1.20181825	6.554093	5.65804692	3.00088148	2.72298091	3.10766008
6.42512428	1.38630248	6.05757081	3.91100195	2.90170965	2.76528883	2.09915257
5.26642917	2.01792808	7.16790824	7.6577129	1.53221117	2.71415707	2.20843182
9.08684244	2.48874303	5.68837256	7.29892514	3.69362529	2.60229504	1.99932815
6.0585498	1.89306011	6.01858143	5.30671356	2.85372663	3.42230505	2.37824653
7.23871738	1.69379063	5.22753559	3.79057357	1.04093076	2.86691052	2.22147572
8.08582581	1.89665909	6.2616966	5.17576125	4.83391135	3.83058152	1.77629904
6.55661351	2.14296174	5.8483286	4.34566774	4.02806984	3.74502428	2.70491789
8.02812053	2.08270177	7.79747399	6.32751514	3.12279229	2.40577984	1.78693556
7.63975435	2.25543459	6.02506307	6.62354692	4.25835312	2.03175092	2.20723549
8.4201609	2.47977909	3.76892288	4.34849143	3.49968101	2.17219454	2.2116353
8.6446676	2.45724798	5.27166027	4.93580682	3.39433944	2.93275459	1.30885192
7.88573452	2.07141746	4.29297587	7.6736461	3.41690569	3.58436895	1.97090523
5.94810748	1.76547142	5.50103729	5.99318095	3.13071816	2.05054386	2.39600243
8.6679686	1.70761749	5.23583261	6.64942015	3.86365882	3.35460171	1.93115588
5.48917844	2.01137637	5.63660227	5.23291068	2.85547155	3.29140453	1.97741846
7.67827256	1.90004557	4.80633487	8.40523423	2.32465927	4.10638628	2.31203948
7.7040434	2.06371702	7.66182703	5.62051823	3.37996342	2.02785261	2.10813512
10.3767079	1.92392522	5.2847432	9.84455474	2.69378248	2.45086423	1.7554014
9.62193857	2.01848371	8.19922853	4.004029	3.84019052	3.38664876	2.1571422
8.54889942	2.22363217	5.20717135	8.26789559	3.85646035	5.37563294	2.01067875
6.00714577	1.77628043	8.06981472	5.26825479	2.61639354	1.85006066	2.6403925
8.23584113	2.16188753	4.29671459	5.65468515	3.27075501	2.61342155	1.61863936
6.23698993	1.27825788	6.68111031	8.98540321	3.90349036	3.02597678	2.62345704
6.20388881	1.6684228	7.35618282	6.03314833	2.866494	1.95981971	2.23086224
6.32768001	1.7163745	6.22934065	5.10596724	2.72381145	2.92901791	1.77260611
4.23365297	3.11549361	5.20027769	6.26321949	2.5049136	2.77790263	2.10477929
8.24156046	2.36198326	7.00300348	7.1935926	1.54385237	2.70251547	2.13829718
5.19478947	2.33834888	6.05602721	8.15515731	2.83890208	2.10255488	2.03917187
3.80612972	2.12522786	4.81719051	3.89441063	2.42883845	2.9935556	1.82910351
5.81883777	1.87104779	5.46174417	4.92539204	2.69826523	2.15527713	1.81225651
6.79353333	1.72227946	5.24210298	9.44710377	3.5781995	2.0766582	1.45241545
6.12559002	2.05209249	6.47961467	6.03796836	3.7274709	4.29568516	1.84845639
5.80110452	1.75784153	6.89904527	3.15680359	3.71359083	2.89047612	2.18818022
7.22750355	2.33834888	6.13204913	7.05032962	4.20709516	3.27976117	1.98945492
4.95558304	2.39123383	5.21545862	6.91326862	4.15497573	3.04013836	2.59712672
4.65821511	2.89135353	6.346294	6.5503486	4.40980082	1.51435513	2.64868259
5.17866395	1.57138779	4.02444307	4.01001466	3.78979647	2.12708235	1.93640905
7.04621963	2.10786306	5.78826148	6.18257005	4.29266009	1.56987511	4.0326789

7.99361051	2.69818781	8.08286489	8.14898299	3.26595755	3.05107588	1.9496239
8.15206497	1.5651666	5.93938719	5.02489491	3.91412657	2.19458829	2.05805682
5.98345444	2.23671729	6.12725081	6.47796906	3.31950361	2.77181074	1.95363822
7.03835555	1.56223407	5.9220734	5.18680503	0.99204727	3.4212064	2.35255659
7.42411371	1.4454637	6.74854253	5.26230906	4.62095432	3.05438444	2.8978161
5.88040924	1.48130245	7.05996447	1.82975484	0.8383335	3.45680807	2.57442046
8.9778088	2.04932687	4.57769245	8.70227026	3.62919104	2.18707258	2.71838476
10.9139815	1.75784153	5.07359991	4.26942795	3.69403478	2.59270912	2.45949098
7.26584206	2.0311604	4.95852729	10.0731322	2.44556022	3.50820225	2.22642884
5.31458124	1.42799015	6.3280898	8.15158945	4.03809347	1.3521028	1.23736473
5.81503612	1.74604587	4.90213923	6.92358287	2.69040566	0.79973556	2.09042646
4.87123857	1.9543738	6.88329402	6.44361633	2.58150829	3.85135808	2.45355018
6.42221699	1.83493985	5.56883719	7.82923944	2.84753096	2.6234603	1.81064131
6.68416092	1.76608657	5.99559886	6.36805627	2.81991277	1.90067022	1.70172982
5.83676531	1.41036749	5.53588549	6.87540054	4.11123915	2.20776541	2.72062212
7.39853061	1.97840299	4.71033378	4.0407086	2.79974481	3.73297101	2.77684465
6.06692776	2.21726529	6.63831037	7.1636628	4.75597676	3.95791739	2.63800242
5.7830318	1.60281555	6.75799812	7.99193111	2.39246183	1.66857518	2.44540454
6.04757416	2.78910857	5.88187834	5.80874551	3.97102479	2.38907774	2.56880169
7.51683913	1.31838115	5.31627399	5.87392888	3.02596152	3.05936757	2.6403925
7.89157053	2.18430013	4.84034813	4.2949562	4.63722915	2.28080426	1.92660126
6.09919768	1.85046626	4.05968724	4.00549123	4.02098672	2.35167671	1.86599418
5.94111135	2.42334502	5.99618692	4.84440299	3.5486276	2.08247346	1.88094851
11.8661571	2.67012409	5.37465485	9.38006342	3.61960015	4.31101737	3.56202141
5.86875436	1.49563884	6.32134071	6.91566967	2.36461435	1.89556089	2.50927824
7.76793485	2.83959017	6.019065	5.28830877	2.60969894	1.90067022	1.95701432
8.25762907	1.81389578	4.9130758	5.11457853	2.67266007	2.83879023	2.01755119
4.96286749	1.84376167	5.59230379	8.04091481	4.23884176	2.65404469	2.30438794
8.04090998	2.08432784	8.05882713	6.07934344	3.34584888	2.43429687	1.66227666
7.80117174	1.75048655	4.05606934	4.98490901	3.61104646	2.26672523	2.13766646
3.86501499	2.13769469	7.79464997	8.88930264	2.53444012	2.89974046	1.93844326
8.51557349	2.06673535	4.43474954	4.68183914	4.07112491	2.28391163	2.55574636
9.23468198	1.93908589	4.95198138	4.74471001	3.1731599	4.46323518	3.84559586
5.43035724	1.94319412	4.28826598	5.00866435	1.60731956	1.93021432	2.81906568
7.6510841	1.94201856	1.44442821	5.56617898	4.72422423	2.2658645	1.96628628
5.98126304	1.32925743	5.72182722	8.2196018	3.50181193	3.13051566	1.85407781
5.2805393	1.6564276	6.82718021	5.08265232	3.38484479	2.46118959	2.29552491
7.79667797	2.13944153	4.4485848	6.71241584	4.73995258	3.09154658	2.203294
6.52337254	2.11266871	6.27185933	7.59024407	1.56139614	2.10839374	2.53795658
1.16659265	1.65395409	6.52572031	8.38970558	3.33668217	2.36124218	1.62831352
3.0972394	1.41206831	5.60049518	5.48784829	4.0925483	1.17394337	2.05132013
8.2445563	2.28849934	4.65035424	6.72221759	3.16295682	3.84470174	2.35566967
7.56179668	2.33203057	6.54885392	1.26480749	3.24262856	2.61731637	1.98795458

6.24154931	2.4040355	6.58957533	5.24356599	2.11118352	2.77181074	2.17271905
5.14009943	2.98376362	6.43613984	4.99760794	4.13703252	2.95170309	2.81976243
7.44466658	1.75995432	6.02572862	6.61703793	3.22746689	3.32617288	2.26241638
10.1817295	1.08478726	7.83641555	6.57480713	1.91079948	2.76991872	2.08684083
6.69628475	1.90104771	4.08930589	7.26970439	4.10197292	3.54449311	2.2481945
7.40572063	2.14546813	4.90852883	5.90008526	4.65096724	3.69574765	2.71692562
7.8118596	2.11930388	7.77933888	7.53734236	3.1661644	4.00141099	2.02006192
7.97980462	1.17402226	4.99068465	6.76927238	3.04919112	2.41903483	1.14232282
6.56891032	1.79164618	5.29106466	4.44732078	4.79529902	4.6302671	1.74459893
5.66802047	1.7774954	7.38277143	4.78443548	3.4089781	3.65067756	1.76910754
7.99223986	1.7719268	9.41680029	5.71798386	4.28046295	3.07031343	1.72164368
8.62177185	1.37362289	5.70341426	3.60311312	4.93377964	3.98623054	2.31871203
5.4186856	3.36173703	6.23890935	5.90008526	2.81332638	2.35418134	2.07862492
9.18871368	1.79587176	1.80502086	7.22051977	3.93783192	1.2388984	2.05910872
6.23708436	1.28562201	4.66213398	7.03100238	3.22517892	2.16336448	1.6303444
8.71697274	1.04941376	7.5710111	1.22528226	1.72018219	2.44756118	2.87926664
6.79554812	0.97100928	2.1547703	3.08470182	4.30349467	1.67706196	3.06441432
9.33798199	1.81418261	4.8164084	6.07404879	0.85167062	1.82756752	2.09297859
6.8777841	1.38339008	7.77064476	6.68385952	2.89879453	0.88329902	2.31931273
5.18042448	2.11029652	4.64161006	0.69778786	2.30946053	3.47551126	0.70032963
12.8515956	1.95221824	6.17245241	5.85941809	3.78022224	2.04164065	2.8587392
5.78675042	2.66507369	5.54147766	7.84821314	5.18626281	3.28577402	2.51947856
5.7606787	1.50664897	4.69106861	6.75298984	5.44015851	3.15111115	2.46626643
5.63504919	1.87468089	6.63389931	7.38485001	1.36121715	2.96945289	3.0959382
0.60638723	1.5939209	3.27336856	6.1872409	3.90349036	0.78262891	3.72572106
5.28352603		0.86934336	1.32976369	3.31913551	3.59441263	1.82976623
6.86747708		1.56879886	2.72033412	2.51838088	1.5386378	3.15072047
8.00808453			1.32976369	2.33312067	1.34004087	2.20723549
6.16640268			6.29684149	5.06398749		2.47741076
7.38764047				2.89429529		2.25789335
5.38803991				2.89370714		3.32783197
8.63238163				4.07320733		2.1862142
10.3231893				4.22214263		3.84798973
9.07639046						1.79008823
7.78544357						
0.84795345						
0.5565352						
0.98346251						
6.21095348						
8.60187851						
8.89395629						
0.94225377						
5.84251978						

7.66438235
1.62174896
