# Supplementary material for: The O-GlcNAc transferase OGT is a conserved and essential regulator of the cellular and organismal response to hypertonic stress
Source: PLoS Genet. 2020 Oct 2;16(10):e1008821. doi: 10.1371/journal.pgen.1008821 (PMC7556452; doi:10.1371/journal.pgen.1008821)
Supplement: S41 Table — (PDF) [file pgen.1008821.s048.pdf]

WT	*ogt-1(dr20)*	*ce* OGT-1	*hs* OGT	*hs* OGTH498A
4.87267624	1.998888921	6.066814141	1.488335015	3.483531773
5.871230929	1.595063178	8.888652059	2.605408397	3.073723931
6.657875043	2.19764833	4.836267686	2.390900075	2.389796374
7.297732219	1.841642993	8.607885737	2.286313314	2.380017006
6.112407228	1.8566157	4.03158846	4.336726476	3.098063374
7.708684285	1.863164697	3.456899632	2.785870976	3.714422796
7.688651953	2.331065925	4.688665454	2.447990003	3.025622895
4.788829633	1.770213332	4.946282734	3.498970785	2.877039078
7.002606354	1.859249522	5.212443674	2.606170115	2.71151751
5.58990771	2.241672003	4.07868687	3.180882532	2.196422648
6.044213278	2.111263183	3.931841751	3.030129806	3.129139627
7.56739463	1.832525948	4.848395634	2.791203592	2.098499755
7.160469552	2.33224461	4.713179608	2.950942876	2.519354762
6.399560634	2.157884719	6.113375087	1.896527613	2.732162204
10.19988955	1.969486878	5.420051672	2.898379838	2.579419576
4.109281039	1.788808972	4.317972382	2.410976736	3.226046049
6.035340433	2.571949697	5.027772617	2.94277868	3.030143463
6.078455092	2.27745164	4.381614723	2.49993114	3.144918317
7.913514498	2.333685014	3.123937504	3.08617028	2.647286957
6.044311896	2.139627668	4.952287968	2.211262267	2.617999285
5.265318518	2.192197021	4.807595056	1.271088248	2.56064482
6.216301777	2.01893279	6.401105247	2.481371342	2.659289244
7.309485014	2.120378148	3.838764113	2.770225941	2.604640178
6.690990128	1.753213371	3.607238047	2.627101733	2.065918103
5.5677227	2.025310892	5.54549257	2.405782312	2.061514287
14.83539222	1.99050559	3.180619413	2.924576375	2.529369769
8.079084869	1.911061908	4.597685108	2.82156439	2.806443589
5.079940674	1.857519225	7.310092342	3.033113285	3.040651905
6.765764953	2.895595308	4.664441504	3.211299549	3.039539791
6.782625972	2.256661132	8.640596749	2.545212536	3.084471217
4.553834118	2.141148947	5.154447264	3.020395828	2.600843326
6.031282347	1.862335611	3.644330889	2.951856077	3.294859643
6.595027872	2.367826705	3.624636519	2.633132104	3.418152281
6.425579256		4.888105905	4.278839669	2.972392373
6.31155219		4.030622244	2.670665785	3.501135247
0.771069511		3.715923661	2.881040481	2.97914781
		4.698462176	5.59029194	3.249655855
		8.503847491	2.922963705	2.968065746
		8.769425653	2.319410093	2.580806012
		4.258589658	2.820572366	3.124089912
		3.919750697	1.728352268	3.043624935
		6.171087509	2.861984145	2.261425093

5.207923313	4.110968697	2.894099228
4.31819875	2.730686934	2.829756503
4.643216134	2.557236557	4.025114671
7.714627393	3.787313726	2.523590554
5.688086455	3.335672206	2.999539939
6.148604824	2.558187537	3.218543616
6.675011439	4.562036349	4.489126837
8.658900423	2.310815513	2.978274416
8.26707787	1.756476343	2.530865479
4.855222992		3.041260731
5.885195852		2.863452749
		1.638974261
		2.804979285
