# Supplementary material for: The O-GlcNAc transferase OGT is a conserved and essential regulator of the cellular and organismal response to hypertonic stress
Source: PLoS Genet. 2020 Oct 2;16(10):e1008821. doi: 10.1371/journal.pgen.1008821 (PMC7556452; doi:10.1371/journal.pgen.1008821)
Supplement: S42 Table — (PDF) [file pgen.1008821.s049.pdf]

rIs4 50mM Na(	Is4 250mM Na	r20 50mM Na(	r20 250mM Na	r93 50mM Na(	r93 250mM NaCl
0.8860054	8.329057615	0.927439758	2.419329506	0.83598681	1.631119966
1.299190611	8.501991438	1.010840358	1.977176974	0.853520767	2.180848689
0.979847575	4.910722158	1.035564918	1.984541371	1.031751633	2.556187633
1.059270394	6.369745966	0.946901311	1.65076752	0.960691016	2.714869486
0.990510736	5.648284426	1.13681328	0.878342655	0.871133856	2.803719346
1.080926588	6.593652188	0.905911906	1.634568155	1.511981864	2.277121946
0.968853773	7.192389926	1.067463281	2.115864718	1.229876381	2.750350088
1.218691742	6.531346287	1.000656274	2.04771825	0.91517305	2.781769836
0.789336426	1.044076818	0.888352258	2.028043204	0.925728575	2.274593088
1.078655734	6.697551597	1.373778831	1.916059949	1.216555339	2.301742492
0.834985426	5.735528836	1.054702115	1.723467988	0.866879234	3.339147558
1.179582586	6.40022322	0.98702966	1.815497191	1.155953418	2.83213709
0.948587117	6.194678953	0.906617445	2.174234304	1.022596429	2.654108757
1.010002003	6.953763079	0.928576127	2.570660871	1.342496466	2.300841966
1.086140061	5.679868878	0.914916578	1.822898834	0.962676736	2.848798866
1.005326068	7.298425006	1.122697335	2.095644644	1.038624587	3.017648253
1.120916025	5.233735457	1.042030383	2.172757845	0.73850733	2.81341715
0.895654133	7.046989072	1.330396342	1.93026255	0.873814268	2.481462847
0.921244251	4.922435207	1.06602271	2.063789289	0.996454867	2.4869149
1.280837732	5.886479812	1.087940934	1.741872681	0.824592442	2.89641172
0.889477208	5.934994118	1.260877681	2.258388852	0.892447793	2.646833239
0.965113025	7.531045902	0.890890408	1.478218158	0.922044276	3.667415842
0.961074895	6.699726519	0.752931001	1.641113909	0.975909405	2.450501596
0.869311365	6.634611066	0.975213368	2.016824242	0.840014853	2.745233157
1.068011105	6.890907	0.953100146	1.893802622	1.13975774	2.445175226
1.027496757	5.634276436	1.03439907	1.738873546	1.191126513	2.284974091
0.913693069	6.384884994	0.900347843	2.534511748	0.976951302	2.799897829
1.095533704	7.763434606	0.993382224	2.307680879	1.101535562	2.876691258
1.161996082	6.988749493	1.159458963	1.92589544	0.860574658	2.25073372
0.911495635	7.824889443	1.05062888	1.795279155	0.821174854	2.575869723
1.069666936	6.080212059	1.009916251	2.385172725	1.212627926	2.724453428
1.085267659	5.897912932	0.992833091	1.774738203	0.97927461	2.246245716
0.913518933	6.4528409	1.21952998	1.878697763	1.272153842	3.953367871
1.095533704	6.613063194	0.953689134	2.027326581	1.143966312	2.127876243
0.950033134	5.066843382	1.121861995	1.54421004	0.966631393	2.765076649
1.082989425	6.022130596	0.837702921	2.652190983	1.192910399	2.274593088
1.595117361	8.00576135	0.901624027	2.367841171	0.980575108	1.423506952
1.139866001	6.216463309	0.950283101	1.292787965	0.851287881	2.994799808
0.871714991	5.275069823	1.002973896	2.16058461	1.147432877	2.653438527
0.858227544	7.008344118	1.033986794	2.25428269	1.00147479	2.315795323
0.881190153	5.673694754	0.917093067	1.906200292	1.075718322	2.849394351
0.804260854	5.744888117	1.007093504	1.952212023	0.952072538	2.78645746

1.033804344	6.285100808	0.742918919	2.50908082	1.229876381	2.971628348
1.108774888	5.104375556	1.03439907	2.45762871	1.017042777	1.416493323
1.143287738	8.239326401	1.662995427	2.41402562	0.83419689	1.230914835
0.982660535	5.499549016	0.970619899	2.183731369	0.887467616	2.33017138
0.853619824	5.249432333	0.951159746	2.293786796	0.992970759	2.282393657
0.997499368	7.822495046	1.11860679	2.321918144	0.889453002	3.155440411
0.961074895	1.164791582	1.071708164	2.28474597	1.059662825	2.732149624
0.858317731	6.072006644	0.912191585	1.818901249	0.857686823	2.860037479
0.712524851	9.099637095	0.916611653	2.43905996	0.784641599	3.029222794
0.988652367	5.341788372	0.858985241	2.032549967	0.894212755	2.662402847
0.900772157	5.634276436	0.875260751	2.257946378	1.040255081	2.366580308
1.14021792	8.227052366	0.78058969	2.105976742	1.038624587	2.541541498
0.835521877	7.413111399	0.948476479	2.410138301	0.850268374	2.569430049
0.904541078	4.43348796	0.978232604	1.787584661	0.937839874	2.366580308
0.914149961	9.007721569	0.972695439	1.752329892	0.991058902	2.199527581
0.895929296	5.732186453	1.157239086	2.169124471	0.857885362	2.828112567
0.981038983	5.919569467	0.908590217	2.375707753	1.392942163	2.883143523
0.9187876	6.221434812	0.779529107	2.633703166	0.985452999	2.491137166
0.999735914	6.182186265	0.957946675	0.997797256	1.202072537	2.019481863
1.007442544	6.675998367	1.014021602	2.47615128	0.877924953	2.909880846
1.028659562	5.103559639	1.014973735	1.937470401	0.93991842	2.895580612
0.78045241	5.248300583	1.172155367	2.247291118	1.000215074	2.46124352
1.220926116	4.650498112	1.03078229	2.161894056	0.863105759	2.729987771
0.844473897	4.726273663	0.976679854	2.186408331	0.892447793	3.786528493
0.883668888	1.060502103	1.116937227	1.950426736	0.873814268	2.826437145
1.033804344	6.333554228	0.982360168	1.927562882	0.97927461	2.755607208
0.769647096	8.729017312	0.948476479	1.486251952	0.883903489	2.58551505
1.02188438	7.562898972	0.877107293	1.973593795	0.986717523	3.142818649
1.023604724	6.623324683	2.096820321	1.828604931	0.964720635	3.155440411
0.912944754	4.439519916	0.846214487	2.217327237	0.927113935	2.410898291
0.853891822	6.076637566	0.95097979	1.710900645	0.9273131	1.613577483
0.888211589	9.995410337	0.847386205	2.1948163	1.155418223	1.991473919
1.083207024	7.308151461	0.685129202	2.267721037	1.127756681	1.939819925
0.969309169	5.182524738	1.168591381	2.754655002	1.133034588	2.262865633
0.932702513	6.81720746	0.957482216	1.204506034	1.032689589	2.022217229
0.902595582	8.617964385	1.092825567	2.303209629	1.055596972	2.494734476
1.042116724	1.033672967	1.138565334	2.463696929	1.019449979	1.954584546
0.878008962	6.182861889	0.761031806	1.254414654	1.227849198	1.240903532
0.939936164	2.980496107	1.125608259	2.256325454	0.846581574	2.357371824
0.870863706	4.997434569	1.094854641	2.40559087	1.110843818	1.472538858
1.110851569	2.056110358	0.999614738	1.873797665	1.221460804	2.15347592
1.006872723	3.97399481	0.863893729	1.881216547	0.93991842	2.868582192
0.872519156	0.959839184	1.151304527	1.949298669	1.040970032	2.45532707

0.928921287	5.169021719	0.929964548	2.222105959	1.073910392	2.137556407
1.333379837	5.683443371	0.863893729	1.561179381	0.913989636	2.943309963
0.934595617	5.730774584	0.855254791	1.22497431	0.840014853	2.422358295
0.916039482	4.417248077	1.129909785	1.448602289	0.851641357	2.890160022
1.041780882	7.154570517	0.951059476	2.20507681	0.818516498	2.875284487
0.991338923	6.519574702	1.04309749	2.115864718	0.943263326	4.049481861
1.198608112	9.369345427	0.83989668	2.014642038	0.831012933	2.408099261
0.960953055		0.84520372	1.304310139	1.02449364	2.76880542
0.896786439		0.774066941	2.1253529	1.147432877	2.255513362
1.166662733		1.088506098	2.474088289	0.766638691	1.274312474
1.024574965		0.920360065	1.374085205	1.16508569	2.694533385
1.164791582		0.984668345	1.334502503	1.308708005	2.863477953
0.821292261		0.791902584	1.241602921	0.949907667	2.517261451
1.10049087		0.815193837	1.429136695	1.034135933	0.625134421
0.743756827			2.580510146	1.397979929	2.510633023
0.965113025			1.11860679	0.848704662	2.151436644
0.875102484			1.054011186	0.996454867	2.515037375
1.249184029			2.05265126	0.893673683	2.948526286
1.238951323			2.146775915	0.887467616	2.6918449
1.091845039			2.461233233	1.118069437	2.595014701
0.93541724			2.416049961	0.661980506	1.113684851
0.633355423				0.860574658	1.522555163
0.97811118				1.183290154	1.04627761
1.042560367				0.866635042	1.325901671
0.776003041				1.090222389	1.396670346
1.219795629				1.034135933	0.97927461
1.055592371				1.030014227	0.929209446
0.657643389				0.743782383	1.001727115
1.328061513				1.239637304	1.326490873
1.846582922				1.002316366	1.302704757
1.045539111				1.042899797	3.625399621
0.797267282				0.974474245	
1.106552003				0.95002507	
0.974392958				1.064601476	
1.006181665					
1.098114467					
