# Supplementary material for: The O-GlcNAc transferase OGT is a conserved and essential regulator of the cellular and organismal response to hypertonic stress
Source: PLoS Genet. 2020 Oct 2;16(10):e1008821. doi: 10.1371/journal.pgen.1008821 (PMC7556452; doi:10.1371/journal.pgen.1008821)
Supplement: S44 Table — (PDF) [file pgen.1008821.s051.pdf]

50mM NaCl	0.72867042	1.166365255	0.959750246	0.972549354	0.961427864	0.700150683
250mM NaCl	2.945028013	4.502543305	3.374998792	2.15370756	5.62669425	3.588959951
50mM NaCl	1.240439975	0.91710628	0.749767362	1.099957807	1.265895661	1.136337735
250mM NaCl	1.304932375	1.15409093	0.821667578	1.150029723	1.048303899	1.359596892

0.759031687	0.954099908	4.35670621	0.816799978	0.98813941	0.842056206	0.95375271
2.766756106	5.503379225	1.596792207	3.267457804	3.03027506	4.760297861	2.052492703
0.957905192	1.103859175	0.935831948	0.723678069	0.922510497	1.010801986	1.194124899
1.118302445	0.769814617	1.045300562	0.93752423	1.187866722	0.753247588	0.969067102

0.650943163	0.870730251	0.569263894	0.781131796	1.039709592	0.8782221	0.95213188
2.68087339	2.301064484	1.923840798	2.338806812	2.469576782	1.465558927	1.433142098
1.03340739	0.660150198	1.571343428	1.06119764	1.076277816	0.960390675	0.860223078
1.031077604	1.109183597	1.207853303	1.01565125	1.306972338	1.012757656	1.117889613

0.814960338	0.848535483	0.867149916	0.746476276	0.821245379		
2.658024487	1.003028108	0.724409189	1.276944224	1.431892931	0.986102009	1.312010452
0.873405198	1.044061075	1.073140943	0.922875414	1.037866281	0.790432473	1.099888762
0.929711528	0.933918368	0.957055985	1.033318163	1.186210235	1.102033199	0.952645589

1.093906494	5.998108089	0.587840244	2.330182893	3.871921269	3.209977623	2.560084832
0.872784706	0.804338337					
1.083743606	1.031103359	0.877642443	1.010736535	0.881099161	1.011744899	1.134288575

6.229919029	3.069546045	2.94544041	4.145034045	3.078739055	1.952716703	3.075561822
0.964591859	1.454004645	1.197813734	0.637715245	1.201125208	1.376783835	0.94620501

1.349649278	0.751450719	1.079719422

ev RNAi

0.819974479	0.897648054
4.632659727	2.414568583
1.240439975	0.91710628
1.304932375	1.15409093

0.823885327	1.156749711	0.89652256	1.31193626	1.016328881	1.117504034	1.188671396
2.426289305	4.727042063	5.419476023	3.448559707	4.033809098	3.349620539	5.420751785
0.749767362	1.099957807	1.265895661	1.136337735	0.957905192	1.103859175	0.935831948
0.821667578	1.150029723	1.048303899	1.359596892	1.118302445	0.769814617	1.045300562

0.733772869	1.101343455	0.934198036	0.840689515	1.160775423		
2.695725212	4.329755052	2.022612891	4.927468013	5.340015696	2.631439802	3.02493625
0.723678069	0.922510497	1.010801986	1.194124899	1.03340739	0.660150198	1.571343428
0.93752423	1.187866722	0.753247588	0.969067102	1.031077604	1.109183597	1.207853303

2.2943897	2.838068139	3.381656461	2.311583553	2.659506667	3.271033326	2.764751845
1.06119764	1.076277816	0.960390675	0.860223078	0.873405198	1.044061075	1.073140943
1.01565125	1.306972338	1.012757656	1.117889613	0.929711528	0.933918368	0.957055985

3.672516745	3.188256041	2.184195991	3.209042906	5.07548249	2.276440332	3.788950244
0.922875414	1.037866281	0.790432473	1.099888762	0.872784706	0.804338337	
1.033318163	1.186210235	1.102033199	0.952645589	1.083743606	1.031103359	0.877642443

3.214322328

1.010736535	0.881099161	1.011744899	1.134288575	0.964591859	1.454004645	1.197813734

0.637715245	1.201125208	1.376783835	0.94620501	1.349649278	0.751450719	1.079719422

rpn-8

3 rnai
