# Supplementary material for: The O-GlcNAc transferase OGT is a conserved and essential regulator of the cellular and organismal response to hypertonic stress
Source: PLoS Genet. 2020 Oct 2;16(10):e1008821. doi: 10.1371/journal.pgen.1008821 (PMC7556452; doi:10.1371/journal.pgen.1008821)
Supplement: S45 Table — (PDF) [file pgen.1008821.s052.pdf]

ev RNAi 50mM	ev RNAi 250mM	ev RNAi 50mM	ev RNAi 250mM	g-1 RNAi 50mM	g-1 RNAi 250m
1.389237843	1.022048229	7.510693309	5.799139029	0.941170284	0.950421416
0.792619915	0.889245705	4.614302764	3.654812846	1.267279888	1.235936753
0.679202569	0.902597742	4.039942261	3.330097083	1.025945266	0.960819631
0.86819103	1.073527317	3.124415577	3.5149239	1.134445788	1.009576276
0.685077158	1.09353188	3.551519324	4.387532437	0.842453108	0.899474612
1.433244505	1.026994226	3.93205744	2.083941917	1.040110785	0.925934781
0.888220385	0.957174196	4.020959585	4.054320112	0.713901004	0.737229593
0.924494761	0.701468092	4.943157924	5.286250037	1.091304713	0.810787808
0.817623542	1.018339866	4.882964696	4.145089539	1.11543883	1.180871292
0.941475467	0.951456309	5.649930209	3.964870449	0.804816073	0.909452506
0.972433619	0.899700489	4.725449705	3.80832907	0.985666012	1.014311131
1.086379429	0.759784586	5.072784407	3.260567583	0.906757725	0.919744831
0.828071707	0.709836483	3.361247909	4.175450757	1.117489269	1.158429084
1.057780187	0.741608555	2.893870007	3.873467835	0.798320325	1.165065609
0.788284478	0.95855673	3.461683604	3.163031949	0.764992654	1.141795709
0.976397819	1.142820642	2.859011213	2.669020297	1.217399162	0.948568743
1.460499647	1.120357337	4.531944643	4.992029501	1.031370358	1.071531358
0.686367322	0.944460307	4.291532389	3.086861881	0.87878748	1.069869094
0.793605107	0.950242717	4.662373233	5.386470397	1.694927962	1.005742749
1.130690522	0.60901406	4.537622552	2.999999996	0.957482215	1.044841391
1.010507404	1.060431247	4.977248668	4.708641013	0.887913717	0.907832662
1.698471246	0.831668645	3.453420114	2.787404261	0.845734784	0.851684049
0.786897467	0.87776721	2.79806779	3.861885714	0.833901457	0.853711869
0.7954507	1.25066428	3.725528597	2.040356467	0.981773518	0.870378695
1.282338661	0.94799467	3.977253502	3.772102736	1.135227194	0.911154198
0.818286606	0.976998761	4.209326644	6.508884402	1.03425139	1.069926102
1.074933379	0.986502114	4.753536716	2.919240949	0.921680558	1.06418377
1.227266795	1.07738435	3.091017667	4.245873781	0.948680029	1.052521482
1.100091394	1.180880049	2.751448099	5.20667475	1.047096244	1.073237778
0.767041747	0.93529079	4.348189062	3.708669488	1.246077941	1.168237294
1.140535931	0.985019952	5.311641485	4.733589906	1.069733317	0.903650446
1.342532828	1.046111499	3.491144741	4.136415989	1.068766108	1.184180979
0.787574951	0.956862311	4.863489337	2.468699138	0.888763395	1.115646192
0.809267462	1.288094453	2.44936987	3.67861312	1.001741793	0.818373033
0.883834112	0.648036319	3.525292877	4.645940915	0.946726225	1.322325449
1.082566042	1.408769181	5.050480638	3.104126209	0.979160433	0.785414919
1.727945342	0.737933798	4.830380386	2.659070002	0.98960812	1.127543977
1.18282632	0.724507601	3.879652189	4.038283313	0.933903748	0.908606867
1.140646287	0.945509707	4.740965521	5.762327787	0.979417262	1.128875198
0.780442197	0.955219982	5.393396795	2.079171611	1.28532449	0.885927411
1.188010786	1.080253678	3.675292824	3.263318537	0.939971443	1.04884601
0.700259214	0.755568246	4.38171996	3.498196557	1.062651886	1.069814372

0.728775496	0.979405339	3.826218559	2.750268414	0.962282426	0.873565633
1.048945979	0.841761711	4.691810911	2.581681078	1.159675611	0.876500892
1.46320363	1.231223903	3.571411308	3.488452277	0.798416289	1.090201797
0.867466813	1.309937015	3.608989289	3.055638532	1.105917172	1.041817873
1.179239583	0.752962838	5.352915302	5.180443055	1.206178894	0.835436691
0.942756386	0.74642093	6.497004168	5.24426954	0.764610513	
0.951369608	0.956517247	4.003079824	3.930723767	1.383194909	
1.129026801	1.094307641	4.843805941	0.932766989	1.13156989	
0.738047042	0.990873785	2.963443786	3.978434568	0.889759767	
1.045921336	1.097962755	4.160537074	2.891646134	0.696038716	
0.924942675	1.012115057	3.52970693	3.480488564	0.727866574	
0.776381677	1.340926062	4.550717961	4.959384738	1.088241402	
1.099168241	1.0952811	4.50311011	4.094235431	0.812530552	
1.205228334	1.027572814	3.262696093	3.119818993	1.05301332	
1.151310224	0.886622561	3.516888296	4.995145624	0.940514175	
0.902944038	0.888194587	6.237343587	3.533980578	1.15592144	
1.034085911	1.04733383	5.732577673	3.464327434	0.954274211	
1.010096128	0.941941746	3.031207766	5.388491586	1.108709892	
0.845166369	0.752616807	3.292282066	4.050319673	1.429688082	
1.378256164	0.779665587	3.03374446	3.386992525	0.788036877	
0.935824353	1.10532684	4.926507359	2.870226533	0.783394539	
1.082245851	0.533009708	5.042589262	6.100266338	0.893562334	
1.026388001	1.022330096	3.926908521	3.849642305	0.904057603	
0.669188684	1.538991542	4.759250491	3.916054932	0.817627195	
0.917827731	1.174511728	3.049227685	4.003530445	0.980573306	
0.845301834	0.656217982	3.369889667	3.289835012	0.959956329	
0.830203401	1.043628639	2.695940921	3.545661403	1.104294573	
1.160622018	1.296433092	3.094287597	5.971367443	1.081288436	
1.196916414	1.384122864	5.194780084	4.23193274	0.768995094	
0.779853628	1.345631066	4.599890632	3.250809057	1.325394249	
1.025581092	1.111242865	3.015589222	7.296116495	1.083760737	
0.890429889	0.817657766	4.119689578	4.696927975	0.761276843	
0.89001477	0.983600104	3.198787514	4.786165042	1.021214096	
0.870340461	1.34137274	4.570225842	4.503013051		
0.70564175	0.680246801	4.401387175	7.055923191		
0.784984244	0.887000135	4.843938594	4.259874641		
0.920039364	1.341133636	5.574674991	4.999099044		
0.991928931	1.04280102	5.061959003	3.105534615		
0.841166258	0.686192196	5.223531847	4.898974165		
1.216285475	0.632808206	5.138994216	4.170676512		
0.870024204	0.887751891	4.538148227	4.893203877		
1.185198751	0.847556573	2.492958896	5.643150335		
1.300255953	0.807276128	3.152250454	3.064759343		

0.691696261	1.007051759	3.754040295	5.429651433
1.578520845	0.64772218	3.491718659	2.888758915
1.131987535	1.005907122	4.404190319	4.487397305
1.968539612	0.816171115	4.706578716	4.688694973
0.9031318	1.089709437	5.858964836	5.033346493
0.913206242	1.121646382	3.977253502	3.929747432
1.178445482	1.417341813	4.587261708	5.371948999
0.74729189	0.880575423	3.422602504	3.425242714
1.001466709	1.248452952	6.16591963	3.599048347
0.8211104	0.922355917	3.838834007	3.398536848
0.832262302	1.078436381	4.627131525	2.992483554
1.211462274	0.813889758	4.078492682	4.526850722
1.202743327	1.487658382	6.587753223	3.809106121
0.94696512	1.398410584	4.439200793	3.553565928
1.171215755	0.854411985	3.899268139	2.968812965
0.994313376	0.830921413	4.863377339	4.175311373
0.924442706	1.33172062	7.552550533	5.410939523
0.956727647	0.868803275	3.310674703	4.177967423
0.851660279	1.319064429	5.721662933	3.780110202
1.070331218	1.490898056	4.154809462	3.599327852
0.84732792	1.270351007	3.697770824	3.400057106
1.136358143	1.315417006	4.133838286	4.590022657
0.994313376	1.284466018	5.264688162	2.502722447
0.959591321	0.707354538	6.562468278	4.642227727
0.909086515	0.891531683	4.292908542	3.501205758
0.985078267	1.099542895	2.818168196	2.511905003
0.968008789	1.001331035	4.693379479	4.429193165
1.131987535	1.019761872	4.357103556	5.160800964
0.667324413	0.920333907	4.218299169	4.084601936
0.631077124	1.061393488	6.600548365	2.451677876
0.815846872	1.037952338	4.876632435	4.182748314
1.155109928	0.85286476	6.140879407	3.069235098
1.00167866	1.263117552	4.560788765	3.436036546
0.913542821	1.284466018	4.583060327	3.821738679
1.011166145	0.965448314	4.263615754	4.385981524
0.88657044	0.713592232	5.148636383	3.597056123
1.054898778	1.122348948	4.488297248	4.288459124
1.198168419	0.820117691	5.20102381	3.100435215
1.084705501	0.690715956	2.601473989	4.05774492
1.033306057	0.652536212	3.606043175	4.712788986
1.123517938	0.903312294	4.931794343	3.609937174
1.159085306	1.588681653	3.729490169	4.228989679
0.818846309	0.846055461	5.985376594	2.854368928

0.979383745	0.985436892	3.048183786	4.165387215
0.919270857		3.463586076	6.07950108
0.808211942		7.193641117	4.424271839
0.972433619		4.290071193	4.43515172
0.939869704		5.809100551	3.518747903
0.760446041		4.22254667	2.568932035
1.120862351		4.370068663	4.318784291
0.976869281		4.151342039	3.747778832
0.846599415		5.016166686	2.810590264
1.002142615		4.450998379	3.097159185
0.867428838		4.811718133	4.713161597
1.473056853		4.519141511	3.374871498
1.035497953		3.705808901	4.393052179
1.330792046		4.311221353	3.47561393
1.114505102		4.941714513	3.696453652
1.204267993		4.360941487	4.200070542
1.144141418		3.65517836	2.999999996
0.961533814		2.927079825	2.305451827
0.906209659		3.010559943	4.618304783
0.873508947		4.641833136	3.193124791
1.103515423		4.31138591	6.695620731
0.723137		6.091477732	3.368155335
0.870024204		4.123016719	3.45114535
1.038732873		4.362149002	4.470608997
1.130690522		3.437132656	2.768908781
1.124459105		3.932363508	2.163857847
0.898968257		3.880718223	5.041419523
1.016741497		3.952550064	4.269693134
0.986442821		3.704304732	4.186708855
1.25597479		3.939374897	3.225249101
0.922898603		5.113611646	4.07416565
1.223770308		4.961722191	2.729490288
1.16149881		4.990470174	2.387789392
1.418245435		3.989379275	4.232899377
0.936864158		3.902613356	4.755331842
0.849840492		3.771855434	3.6320459
0.747859633		4.385875437	3.705835377
0.688370798		3.56037424	2.048501839
1.029154545		6.004369803	3.32063658
0.92673868		4.271291699	3.885779549
0.839772		3.240725076	3.832341233
0.8677644		3.902210983	3.850587405
0.892852827		4.517851065	5.56601941

0.896282479	4.624713375	2.602294789
1.047301863	4.889235197	2.714722699
0.975552746	4.368458765	5.754407759
0.984843724	5.798416948	3.426438679
1.075698456	3.403216914	3.059364515
0.917827731	3.457430096	2.812946688
1.021992335	5.040768175	4.650652823
1.07079902	5.819822921	3.947670788
0.792343471	3.090604632	4.746147484
0.832448407	4.552425547	5.742834414
0.924175088	4.224921303	4.737784492
0.640017805	4.37899628	4.320476605
1.434419296	3.370856107	3.819596316
1.161012857	3.386572289	2.970873782
1.144533382	5.878330648	5.28897772
0.750425189	3.82266061	4.42751544
0.96045131	3.61323369	4.587378635
1.113630981	3.30712545	5.676666295
1.208557694	6.395317924	3.330903402
0.832932671	3.810763821	2.554817024
0.87479233	2.646432541	2.774271841
1.226070628	5.199024186	5.376085839
1.000566919	5.856078776	5.987484822
0.921444074	3.123379869	3.068157694
1.424080538	3.752125945	3.646226115
0.908385059	5.409920086	7.146653031
0.986442821	5.655516541	7.109669764
1.299609595	6.572449803	2.80865132
0.923850223	4.292168002	4.103155334
1.740048407		3.292903791
0.831403274	6.106806558	2.921124331
0.974483764	4.458927802	5.375995411
0.971402929	4.545432574	4.43029222
0.883834112	4.619732914	3.95404651
0.916497546	4.925391334	5.004065527
1.081679823	4.108950638	6.117823058
1.199489151	4.852249273	3.863632842
0.883834112	5.285731039	3.380950782
0.873287165	4.541593526	3.809106121
0.805271079	5.489730186	3.091046651
0.859351172	4.456515661	3.679233169
0.908300108	4.011247122	5.905848998
0.896282479	3.502694846	3.935069303

0.783977854	3.903600659	4.891528122
0.735290563	4.637569721	3.177598859
0.943391666	2.871153416	4.975859348
	4.640129086	3.673378195
	3.991307401	5.399570262
	4.062393485	4.478899541
	5.614946121	4.932614346
	4.36323169	4.558377965
	5.016605463	3.099984177
	4.129969413	4.29827821
	3.467904961	5.205467545
	5.686486412	2.704138985
	3.688683059	8.175407068
	4.034894857	3.640565021
	5.479771492	4.87122841
	4.218965041	6.327605456
	3.583111263	7.096821376
	6.227947344	5.120055183
	5.865074289	5.957556201
	3.449527309	
	5.593942582	
	7.030950011	
	3.595535674	
	3.135741068	
	6.511979827	
	3.224800137	
	5.681790717	
	3.988715328	
	4.113460814	
	5.246260456	
	5.002834594	
	4.982823255	
	4.325626072	
	4.453637626	
	7.753358551	
	3.549421452	
	3.522433759	
	3.791785722	
	4.62344343	
	3.084400675	
	5.785096003	
	3.834186829	
	4.652636172	

3.961019814
3.745395675
4.791871689
4.385728186
8.442513569
4.579867669
4.766391102
2.050130671
3.412659156
4.675960198
9.143111499
4.624021829
3.370553815
5.185160121
6.274673227
4.415001529
5.716225813
9.178277313
4.151122508
2.278858763

dr20 lgg-1 RNAi 50mM NaCl	dr20 lgg-1 RNAi 250mM NaCl
0.948270232	1.265457858
1.273465733	1.313402875
2.083399907	1.058692818
1.427311691	1.219588384
1.328405022	1.176225241
1.002020209	0.960207623
1.265743284	1.177533612
0.905386152	1.580947905
0.886123504	1.552203397
0.869910838	1.275362243
1.068500906	1.254040033
2.062288626	1.093892645
1.028541638	1.141690523
1.20887728	1.359697754
1.208920209	1.876955172
1.169965671	1.1212932
1.101293773	1.185152947
1.399802348	1.413960282
0.957941549	1.198560998
1.342135474	1.475831688
1.191164219	1.465116078
1.122887768	1.426816493
1.052923223	1.338251037
1.31212704	1.172831001
1.528519805	1.58287003
1.175849444	1.320585547
0.902737663	0.910356258
1.298961886	1.053744775
1.063545914	1.330145971
1.356951255	1.352152338
1.142973013	1.308826558
0.972022269	1.184237996
1.442040608	1.42761182
1.083516633	1.037079559
1.206178894	1.105711155
1.428858074	1.15131626
1.223410021	1.17356618
1.263615984	1.638118714
1.242729769	1.167632825
1.057504756	1.563134407
1.688650451	1.437225368
1.296029692	1.07439929

1.14762652	1.20922361
0.9688978	1.044841391
1.087667867	1.148267425
1.181180367	1.024454242
1.068500906	1.414992047
1.298961886	1.15679115
0.984316973	1.105844389
1.361001856	1.185710928
0.892885675	1.103743577
1.258281654	1.091066738
1.251695078	1.052521482
1.137432946	1.085645151
1.063381896	1.178362861
1.443827523	1.588301151
0.975664705	1.03116883
1.380147003	1.273332956
0.955258467	1.37252712
0.962827012	1.201039511
1.449851397	1.211599813
1.100373728	1.098135598
1.215316613	1.208330645
0.976725839	1.627840427
1.683907051	1.075910848
1.331956907	1.385078253
1.184639985	1.485626046
1.044526052	1.573393887
1.173312984	1.128347357
1.220960498	1.213049036
1.306940767	1.4806073
1.075702811	1.2790805
1.50580295	1.076107397
1.069042275	1.146471574
1.174520655	1.108410437
1.055406532	1.399527653
1.108471665	1.432803136
1.486012397	
1.058282299	
1.120234943	
1.2217626	
1.233284037	
0.852853763	
1.24700341	
1.057855271	

1.326796783
1.027159642
1.488302093
0.921680558
1.104919431
0.983350664
1.122994142
1.434647319
1.174950961
1.168698759
1.007865697
1.143610536
1.100253835
1.252451741
1.153975251
0.983499713
1.076335273
1.079070356
1.034712286
1.373901994
1.154439316
1.018374724
1.333145093
0.971347605
1.116688202
2.088116149
1.033418896
1.283169036
1.067864956
0.837347331
