# Supplementary material for: The O-GlcNAc transferase OGT is a conserved and essential regulator of the cellular and organismal response to hypertonic stress
Source: PLoS Genet. 2020 Oct 2;16(10):e1008821. doi: 10.1371/journal.pgen.1008821 (PMC7556452; doi:10.1371/journal.pgen.1008821)
Supplement: S43 Table — (PDF) [file pgen.1008821.s050.pdf]

	Unadapted					Adapted				
WT	0	0	0	0	0	15	20	25	35	20
ogt-1(dr20)	0	0	0	0	0	0	0	0	5	0
ogt-1(dr93)	0	0	0	0	0	0	0	10	0	0
