# Supplementary material for: The O-GlcNAc transferase OGT is a conserved and essential regulator of the cellular and organismal response to hypertonic stress
Source: PLoS Genet. 2020 Oct 2;16(10):e1008821. doi: 10.1371/journal.pgen.1008821 (PMC7556452; doi:10.1371/journal.pgen.1008821)
Supplement: Attachment — Submitted filename: Urso et al reviewer response 200708.docx [file pgen.1008821.s053.docx]

We thank the editor and reviewers for their careful and complete evaluation of our manuscript. All comments have been addressed through either the inclusion of new data or text modifications. Briefly, the following three new pieces of data were added:

1. Characterization of an *ogt-1∆TPR* mutant that exhibits a Nio phenotype (Figure S6).
2. *ogt-1* mutants fail to develop post-embryonically under hypertonic conditions (Figure S4C and text-based data on page 10, paragraph 2).
3. *gpdh-1* mRNA expression levels continue to be upregulated in the *gpdh-1::gfp* CRISPR knock-in allele in an ogt-1 mutant background (Figure S3C,D).

All other comments were addressed by text changes as detailed in our responses below.

**Reviewer #1**

The authors state, “OGT-1 is required for the accumulation of GPDH-1 protein during hypertonic stress”. Formally, this has not been demonstrated as they are relying on a reporter (either just GFP, or GPDH-1::GFP). In the absence so far of direct proof, it would seem prudent to … (perform) a Western blot against endogenous GPDH-1.

- We apologize for the confusion. The experiment suggested by the reviewer was performed in Fig 3E using a CRISPR tagged allele of the endogenous *gpdh-1* gene. This tagged protein was functional since it did not exhibit a defective hypertonic adaptation phenotype like the *gpdh-1(ok1558)* LOF allele (Fig S4D). We also show in new data that *gpdh-1* mRNA continues to be upregulated by hypertonicity in the *ogt-1(dr20)* mutant (Figure S3C,D).

It is not clear what mechanism they favor to explain their observations. Is it the inducible nature of gene expression that renders any translated transcript subject to this control?

- This is one possible mechanism. If this is the case, it must be specific to hypertonic stress since heat shock and UPR-ER inducible reporter expression is not inhibited by loss of *ogt-1*. Such mechanistic studies will be the focus of future work.

In the absence of detailed molecular insights, which are obviously beyond the scope of the current report, the authors might be expected to test the effect of *ogt-1* on the salt-induced expression of another protein by Western blot, or as a minimum, *nlp-29*, for which robust transcriptional reporters exist.

- In an exhaustive genome-wide RNAi screen for regulators of the *nlp-29p::GFP* induction by infection (Zugasti et al, 2016), *ogt-1* was not identified. We also showed that *nlp-29* mRNA, as well as another osmotically induced mRNA (*hmit-1.1*) continue to be induced by high salt in *ogt-1(dr20)* (Fig 3A). Unfortunately, methods for detecting the NLP-29 protein are not available.

Love et al (ref 27) reported that the expression of genes such as Y65B4BR.1 and C55A6.6 was strongly affected in ogt-1 mutants. Alternatively, Bond et al (ref 29) found that the induction of *ilys-2* and *clec-60* expression by *S. aureus* is *ogt-1* dependent. What about in the new alleles?

- This is an excellent suggestion that our collaborator Dr. Hanover will be examining in future studies.

The authors state, “expression of ogt-1 in intestine, muscle, or neurons did not rescue (Figure 5A - B)”, and “Overexpression of a human OGT cDNA from the native *C. elegans ogt-1* promoter partially rescued *gpdh-1p::GFP* induction by hypertonic stress in an *ogt-1* LOF mutant (Figure 5C - D)”. But the results appear to be quantitatively equivalent in the two sets of experiments, so how do they justify coming to opposite conclusions? If they consider that expression of *ogt-1* in intestine has no effect, they cannot claim even partial rescue of this phenotype with human OGT. This is especially important as the authors use it to argue for an ancient osmoregulatory function for OGT independent of O-GlcNAcylation catalytic activity.

- When analyzed quantitatively, statistical analysis (1-way ANOVA with post-hoc testing) shows that human OGT rescue is significantly different from the control *ogt-1(dr20)* allele, whereas the intestine, muscle, and neuron expression of worm *ogt-1* are not statistically different from the *ogt-1(dr20)* allele. All of the underlying raw data used to draw these and all other conclusions are now included in a supplemental Excel spreadsheet.

In Figure S5, “Images are not exposure matched in order to highlight the weak RL2 staining in OGT-1H612A”. OK, but this does not allow comparison; the authors should include a set of images taken with the same high exposure for all conditions.

- The images are now exposure matched

**Reviewer #2**
Is the ability of OGT to interact with one or more of its many protein partners, via the different TPRs, necessary?

- This is an excellent question. We generated a deletion allele of the entire TPR domain at the endogenous *ogt-1* locus using CRISPR/Cas9 editing and found that the TPR domain is required for the Nio phenotype. Interestingly, this deletion did not alter OGT-1 protein abundance or localization, based on knock-in of this allele into the *ogt-1::gfp* CRISPR strain. Loss of the TPR domain also eliminated O-GlcNAcylation activity based on RL2 antibody staining. This new data is now included in Fig. S6.

In the results text describing Fig. 3C, the authors should add some description of the *dr34* allele, that it is the CRISPR allele analogous to the *dr20* mutant allele. The description is currently only in the figure legend.

- Additional description has been added.

Additional description of the results for Fig. 3A,B, D would be helpful. It is a bit confusing to compare levels of gpdh-1 mRNA with the transcriptional reporter in the *dr20* allele (where they are higher than in WT), to levels with the translational reporter and the *dr34* allele (where they are not different from WT).

- Additional description has been added to reflect these differences.

The authors refer to Fig. S4B to support the claim that *ogt-1* worms cannot reproduce under mild hypertonic stress. Fig. S4B shows images of plates after growth for 5 days on 50mM NaCl and 10 days on 250mM NaCl. There are clearly fewer worms in the *ogt-1* strain, consistent with an inability to grow, but it’s not as clear that they can’t reproduce. It seems a failure in growth alone could be consistent with the data shown. Additional analysis or description of the results seem to be needed in order to conclude there are reproduction defects.

- We more closely analyzed this phenotype and found that *ogt-1(dr20)* mutants have apparently normal germlines under hypertonic conditions (not shown) and lay the same number of eggs as WT under both isotonic and hypertonic conditions. However, the eggs laid by *ogt-1* mutants under hypertonic conditions do not develop to reach the L4 stage. Therefore, the chronic adaptation phenotype is a developmental defect. These new data are presented in Fig S4C.

After seeing that the catalytically dead human OGT rescues the *ogt-1* LOF stress response, the authors use CRISPR to engineer two mutations analogous to the human alleles. They state “only the K957M mutation suppressed O-GlcNAcylation activity completely.”, but no figure is referenced to show this result. It is not clear how activity was assayed?

- We assayed O-GlcNAcylation activity by staining WT or mutant embryos with the O-GlcNAc-specific monoclonal antibody RL2. As shown in Fig. S5A, the K957M allele exhibits no detectable RL2 staining.

*C. elegans* is singular; some sentences need to be edited to reflect this point.

- Corrected

**Reviewer #3**

The authors should address whether the discrepancy in hypertonic stress transcripts and proteins is due to the multicopy array by testing the gpdh-1::gfp transcript levels in this knockin.

- We performed qPCR in the *dr84 gpdh-1::gfp* knock-in allele and found that *gpdh-1::gfp* mRNA was induced to levels equal to or slightly elevated as compared to WT while GPDH-1::gfp protein levels were reduced by ~50%. These new data are now shown in Fig. S4C and D.

The authors then go on the show that while ogt-1 is not needed for survival after hypertonic stress, it is required for adaptation to the stress, although I would ask the authors to be more clear on the “adapted” phenotype as it’s not obvious to a reader naïve to hypertonic stress.

- We apologize for the lack of clarity. We now more precisely describe these phenotypes as acute survival, acute adaptation, chronic adaptation, or genetic adaptation and define each in the text. We think these descriptors better clarify phenotypic differences for the reader.

The authors should indicate which 3’-UTRs are used in all of their reporter strains/genes so that readers can see if there’s a common thread (including the constitutive RFP transgene).

- This is clearly not a transgene issue since all of the molecular phenotypes are observed with the endogenously tagged *gpdh-1::gfp* allele. Our *gpdh-1* and *col-12* transgenes utilize the *unc-54* 3’UTR, which is now indicated in the methods.

Also, the authors use a GFP knock-in into the endogenous *gpdh-1* gene to suggest that the discrepancy is not due to a transgene effect, but the decrease in GFP protein levels is low in the *ogt-1* mutant and they do not show the *gpdh-1::gfp* transcript levels. They need further data to rule out a transgene or multi-copy array effect.

- We now measure the transcript levels in this knock-in mutant and show that mRNA levels are equivalent to or slightly higher than WT in the *ogt-1(dr20)* mutant (Fig. S3 C,D).

The second possibility is that *ogt-1* is required for a global increase in protein translation, independent of osmosensitive genes/transcripts. This can be tested by increasing the number of osmosensitive gene transcripts they test (via qPCR) as well as testing some endogenous protein levels, and by testing the transcripts and protein levels from control genes, not expected to increase under hypertonic stress. These types of data are required to make claims like this, “These data unexpectedly suggest that OGT-1 regulates osmosensitive gene expression at a post-transcriptional level” (pg. 8).

- Unfortunately, testing this possibility would either require us to generate antibodies against other hypertonicity-induced proteins or generate tagged CRISPR alleles for protein detection. Unlike *gpdh-1*, we do not know if these other proteins have mutant phenotypes and would be unable to determine if such tagged alleles are functional. Non-functional tagged alleles would be uninformative. Therefore, we have restricted our conclusion to observations related to *gpdh-1* only.

A final possibility that is not addressed is that there may be transcriptional regulation by ogt-1, but only at the tissue-specific level, such that total transcripts may be unaffected, but hypodermal transcript levels decrease. This should at least be addressed in the discussion. Although it could be easily tested with smFISH.

- We have attempted smFISH against *gpdh-1* and *gfp* mRNA but have so far been unsuccessful in detecting a specific signal. This possibility is now mentioned in the discussion.

The sentence in the Abstract “mutations that ablate O-GlcNAcylation activity in either human or *C. elegans* OGT rescue the hypertonic stress response phenotype” is unintentionally misleading as its suggesting that the mutation itself is rescuing hypertonic stress. In reality you rescue with a putative catalytically dead OGT. Please rephrase.

- Corrected

Results on pg 9. Please explain what you mean by adapted and unadapted phenotypes in the main text. This is not obvious to a reader naïve to hypertonic stress

- Corrected

In the Introduction the author write, “Hypertonicity contributes to several pathophysiological conditions and is also a feature of normal physiological states such as those that exist in the kidney and thymus (3, 4).” This sentence can use more context, especially since the meaning of the latter part of this sentence is not clear.

- Additional context is now provided

In the intro, please clarify this sentence. “These organic osmolytes track extracellular osmolarity to maintain intracellular water content and cell volume.”

- Clarified

In the intro please clarify what does “through phylogeny” mean in the statement “there is a significant chemical diversity among osmolytes through phylogeny”.

- Clarified

Please provide more context to these sentences “OGT proteolytically cleaves and activates the mammalian host cell factor C1 (HCF-1) (15, 16). OGT also has non- catalytic scaffolding functions in cell adhesion and neuronal synaptic transmission (17, 18).” What is HCF-1? What do you mean by scaffolding functions? What about the serine/threonine phosphatase activity indicated in WormBase? Given your phenotype these might be related to these functions, it would be useful to know more about them.

- Clarification provided. OGT does not have phosphatase activity in any known system.

In Methods: Define RFLP.

- Corrected

Methods: Please provide a protocol or reference for “freeze-cracked”.

- Reference provided

Methods: Please describe the methods used for osmotic stress resistance, given its importance to this manuscript. Also please paraphrase the survival assay. Also please note any changes between the published method and the method is this manuscript.

- Further description now provided.

Results, pg. 7, the nested double brackets are confusing. “( Nio (no induction of osmolyte biosynthesis gene expression) mutants; Figure 2A) “

- Clarified

Methods: Please add RNAi to methods, especially concerning timing of when each animal stage (L1, L4, adult) is put on RNAi, when they were put on hypertonic plates, and when they were ran into the worm sorter.

- A RNAi methods section has been added

Results: It is noteworthy enough to state in the results that the *dr20* rescue with *drEx465* overexpression leads to increased GFP expression on hypertonic media.

- We now state this in the results

Results: This statement is not exactly true. “The function of *ogt-1* in the response to hypertonic stress is specific because inhibition of *ogt-1* did not affect either heat shock or endoplasmic reticulum stress inducible reporter expression (Figure S2)” RNAi of ogt-1 resulted in a significant increase in GFP expression from *hsp-16.2p* reporter after heat shock, suggesting *ogt-1* suppresses the heat shock response.

- We now state that *ogt-1(RNAi)* is specific because it does not decrease either heat shock or ER stress inducible reporter expression.

For Results, pg. 9 why is *gpdh-1(ok1558)* a presumptive null? Reference or explain the mutation.

- *ok1558* creates a frameshift in exon 2 resulting in an premature stop codon. This is now described in the manuscript.

In the Discussion (pg. 14) the authors state, “OGT-1 is required for the accumulation of GPDH-1 protein during hypertonic stress, but not for the upregulation of gpdh-1 mRNA.“ This should be restated as they don’t show any data on actual GPDH-1 protein levels.

- We apologize for the confusion. The Western blot in Figure 3E reflects endogenous GPDH1 protein levels, since this is a functional CRISPR tagged allele of the endogenous locus.

Figure 2E: What is the statistical significance between WT and *ogt-1(dr20 dr36)*? There seems to be a significant reduction in the *ogt-1(dr20 dr36)* compared to WT?

- The statistical significance is now indicated.

Results section, pg 9: “Importantly the requirement for ogt-1 … confirmed to be functional (Figure 3E and S4C).” Please write out in the text how it was confirmed to be functional so as to prep the reader when they go to look at the figures.

- Description now included

**All Reviewers:**

Have all data underlying the figures and results presented in the manuscript been provided?
Large-scale datasets should be made available via a public repository as described in the *PLOS Genetics* [data availability policy](https://nam05.safelinks.protection.outlook.com/?url=http%3A%2F%2Fjournals.plos.org%2Fplosgenetics%2Fs%2Fdata-availability&data=02%7C01%7Cstl52%40pitt.edu%7Cfe5e9899464f4e3bd8ed08d80caed5ec%7C9ef9f489e0a04eeb87cc3a526112fd0d%7C1%7C0%7C637273292348204713&sdata=w8unISfq1ytwoIjx15QCARaVjObRbofzagT2UkLGw2k%3D&reserved=0), and numerical data that underlies graphs or summary statistics should be provided in spreadsheet form as supporting information.

Reviewer #1: None Excel file with all underlying data now included.

Reviewer #2: Yes

Reviewer #3: Yes
