# Supplementary material for: The O-GlcNAc transferase OGT is a conserved and essential regulator of the cellular and organismal response to hypertonic stress
Source: PLoS Genet. 2020 Oct 2;16(10):e1008821. doi: 10.1371/journal.pgen.1008821 (PMC7556452; doi:10.1371/journal.pgen.1008821)
Supplement: Attachment — Submitted filename: Urso et al reviewer response 200821 FINAL.docx [file pgen.1008821.s054.docx]

We thank Reviewer 1 for their suggestions. We have tried to more fully address their concerns as detailed below.

**Reviewer #1**

I am not convinced by the authors’ minimalist answers to my previous comments. I will try to explain my concerns more clearly. “The function of ogt-1 in the response to hypertonic stress is specific because inhibition of ogt-1 did not decrease either heat shock or endoplasmic reticulum stress inducible reporter expression (Figure S2)”. There is a very marked increase in hsp-16.2p::GFP expression upon ogt-1(RNAi). There is no comment on this, but it clearly indicates that the influence of ogt-1 is far from being specific to the upregulation of gpdh-1 expression as stated. Here, ogt-1 is influencing the level of an exogenous protein, GFP.

- We have changed the wording of this statement to reflect that *ogt-1(RNAi)* leads to a significant increase in *hsp-16.2p::GFP* expression following heat shock. We have removed reference to the specificity of *ogt-1* in the response to hypertonic stress.

I had previously commented, ““OGT-1 is required for the accumulation of GPDH-1 protein during hypertonic stress”. Formally, this has not been demonstrated as they are relying on a reporter (either just GFP, or GPDH-1::GFP)”. The authors apparently missed my point; I had understood that the GPDH-1::GFP was produced from the CRISPR tagged allele of the endogenous gpdh-1 gene. The point is that many studies have shown that the stability of chimeric proteins can be influenced by the in vivo tolerance of the fluorescence protein to proteasomal-based degradation. So, no, they have not “measured GPDH-1 protein levels in an ogt-1 mutant”, rather the levels of a GPDH-1 translational reporter (GPDH-1::GFP), and in the absence of other evidence, they cannot claim that there is a specific effect on GPDH-1.

- We have changed the wording to indicate that we measured GPDH-1::GFP protein levels in an *ogt-1* mutant.

This is tied to a second point: “In the absence of detailed molecular insights, which are obviously beyond the scope of the current report, the authors might be expected to test the effect of ogt-1 on the salt-induced expression of another protein by Western blot, or as a minimum, nlp-29, for which robust transcriptional reporters exist”.

The authors answered: “In an exhaustive genome-wide RNAi screen for regulators of the nlp-29p::GFP induction by infection (Zugasti et al, 2016), ogt-1 was not identified. We also showed that nlp-29 mRNA, as well as another osmotically induced mRNA (hmit-1.1) continue to be induced by high salt in ogt-1(dr20) (Fig 3A). Unfortunately, methods for detecting the NLP-29 protein are not available”.

(i) Genome-wide RNAi screens are known to suffer from a significant level of false-negatives. (ii) The screen was for regulators of induction by infection. (iii) More importantly, there was no suggestion that they needed to detect the NLP-29 protein. What was proposed was a simple experiment using a nlp-29p::gfp *transcriptional* reporter. Yes, they show that dsRed levels don’t change in the ogt-1 mutant, but this is not an adequate control since the expression of the col-12p:dsRed reporter is not affected by hypertonic stress. By testing the nlp-29p::gfp reporter (or any other GFP reporter of a hypertonic stress-induced gene) with and without hypertonic stress in wild-type and ogt-1 mutants, they will go some way to providing the essential supporting evidence that the effect of ogt-1 is likely to be linked to GPDH-1 and not GFP.

- We obtained the *nlp-29p::GFP* transcriptional reporter from the CGC (IG274 *frIs7*) and performed the requested experiment +/- hypertonic stress +/- *ogt-1(RNAi)*. As previously reported (Pujol et al, *Current Biology* 2008), the induction of GFP in this reporter is non-normally distributed. We found that only a minority of animals strongly induce GFP in response to hypertonic stress in contrast to our *gpdh-1p::GFP* reporter in which virtually all animals induce. Nevertheless, *ogt-1(RNAi)* did not inhibit the overall mean hypertonic induction of *nlp-29p::GFP* reporter. Hypertonic induction of the *gpdh-1p::GFP* reporter was completely blocked by *ogt-1(RNAi)* in these experiments. In one replicate, mean *nlp-29p::GFP* hypertonic induction in *ogt-1(RNAi)* exhibited a small but statistically significant increase relative to the *empty vector(RNAi)* control (now in Figure S3) while in another replicate, induction levels were statistically equivalent. Regardless of whether or not there is slightly elevated hypertonicity induced *nlp-29p::GFP* reporter expression, these data, in conjunction with our *hsp-16p::GFP* and *hsp-4p::GFP* data, strongly suggest that the effect of *ogt-1* is linked to *gpdh-1* and not GFP.

I previously commented, “If they consider that expression of ogt-1 in intestine has no effect, they cannot claim even partial rescue of this phenotype with human OGT”.

They replied saying that their statistical analysis proved their point. But the measure that they used is as much affected by the distribution of the data as the relative values, and I question the validity of the comparisons that they made. I had based my previous comment on the simple inspection of the graphs, from which I maintain that any right-minded person would not try to draw such opposite conclusions for the tissue-specific rescue and the rescue with the human protein. Indeed I suggest that the most parsimonious explanation for the results in Figure 5D is that neither the human protein, nor its catalytically dead version are capable of rescuing to any real degree the mutant phenotype. The authors have now provided data for a single set of experiments (it is not stated how many times each experiment was performed). This allows one to calculate a “degree of rescue” for sample N:

= (fold change N – fold change ogt-1 mutant)/ (fold change WT – fold change ogt-1 mutant)

So first, the effect of high salt can be expressed as a fold-change relative to the control:

For Figure 5B

dr20 = 1.95
ogt-1 promoter* = 4.41
dpy-7 promoter = 8.65
nhx-2 promoter* = 2.65
myo-3 promoter = 2.52
rab-3 promoter = 2.12

* [the average value for the 50 mM samples for ogt-1 promoter and nhx-2 promoter were 0.87 and 0.95, respectively. As there were values missing in the table, this is assumed to be a data-entry error, and the expected value of “1” was used to calculate the fold-change]

For Figure 5D

WT = 6.51
ogt-1(dr20) = 2.07
ceOGT-1 = 5.35
hsOGT = 2.84
"hsOGT H498A" = 2.87


For Fig 5B, there is no WT control, but as the values of ogt-1(dr20) are very close in the 2 experiments, one can reasonably take the WT from Figure 5D

So the “degree of rescue” is:

For Figure 5B

dr20 = 0
ogt-1 promoter = 0.54
dpy-7 promoter = 1.47
nhx-2 promoter = 0.15
myo-3 promoter = 0.12
rab-3 promoter = 0.04


For Figure 5D

WT = 1
ogt-1(dr20) = 0
ceOGT-1 = 0.74
hsOGT = 0.17
"hsOGT H498A" = 0.18

This is the numerical demonstration of what one can see from the graphs: that rescue with hsOGT is no stronger, in any meaningful way, than that with the intestinal promoter nhx-2. I consider that the authors have not adequately addressed this point in their rebuttal.

- We thank the reviewer for the excellent suggestion. We have re-analyzed all of the data in Figure 5 to calculate a ‘Degree of rescue’ for each tissue specific expression transgene, using the formula suggested by the reviewer. Since the original experiment in 5B did not contain WT data, we thawed all of the strains and performed the entire experiment again to include this genotype. Using the ‘Degree of Rescue’ analysis suggested by the reviewer (calculation now outlined in the ‘Methods’), we found that both the muscle-specific and intestine-specific promoters, in addition to the native *ogt-1p* and hypodermis-specific promoter, conferred a statistically significant ‘Degree of Rescue’. Neuron specific expression did not provide significant rescue. Likewise, both of the human cDNA transgenes provided a statistically significant ‘Degree of Rescue’. We now include the ‘Degree of Rescue’ data in Figure 5 and have moved the GFP::RFP fold changes into Figure S5. All of these raw numerical data are also included in the supplemental Excel data file.

If the results are valid, it might be appropriate to cite PMID: 32628333

- We have cited the manuscript as requested.

**Reviewer #2:** This revised manuscript from the Lamitina lab identifies the O-GcNAc transferase OGT as an essential and conserved regulator of hypertonic stress responses. Their study characterizes a novel role for OGT in an essential aspect of cell physiology. The results show a role for OGT-1 in regulating osmosensitive gene expression, unexpectedly, at a post-transcriptional level. The authors use tissue-specific promoters to demonstrate that OGT-1 function occurs in the hypodermis to regulate GDPH-1 levels, and show that expression of a catalytically dead human OGT cDNA can rescue ogt-1 LOF mutants. Taken together, these results underscore the importance of OGT function outside of its catalytic O-GlcNAcylation activity, and uncover a novel role in regulation of gene expression post-transcriptionally that is independent of O-GlcNAcylation.

The researchers have included additional experiments to address the role of the OGT TPR domain, and added experiments to clarify the developmental phenotype of ogt-1 mutants. These experiments are a significant contribution to the manuscript. The authors have also clarified the descriptions of several results, increasing the readability. It appears they have addressed all reviewer concerns, with the exception of experiments for which there are not reagents.

The research is very well-designed and executed, with an elegant combination of classic and cutting-edge genetic experiments to support its claims and includes all relevant controls. The manuscript is also very clearly written and accessible to readers. This study should be of broad interest to researchers studying the molecular mechanisms of adaptations to stress.

- Thank you

**Reviewer #3**: Looks good to me. I have no further comments for the authors.

- Thank you
